# MeCP2 requires interactions with nucleosome linker DNA to read chromatin DNA methylation

**DOI:** 10.1038/s41467-026-71741-0

**Published:** 2026-04-17

**Authors:** James A. Watson, Beatrice K. Alexander-Howden, Theo S. Hall, Martin A. Wear, Finlay McGhie, Gillian Clifford, Hannah Wapenaar, Juan Zou, Adrian Bird, Marcus D. Wilson

**Affiliations:** 1 Centre for Cell Biology, School of Biological Sciences, University of Edinburgh, Edinburgh, UK; 2 Institute of Quantitative Biology, Biochemistry and Biotechnology, School of Biological Sciences, University of Edinburgh, Edinburgh, UK

**Keywords:** Autism spectrum disorders, Chromatin structure, Nucleosomes, DNA methylation

## Abstract

Methyl-CpG-binding protein 2 (MeCP2) is a clinically important epigenetic reader that is essential for neuronal function, but how it binds methylated DNA within the protein-DNA complexes that comprise chromatin is unclear. Using designer nucleosomes, we observe that MeCP2 is able to engage methylated DNA at multiple sites on the nucleosome surface. Surprisingly, even methyl-cytosine placed in bent, histone-contacting, core nucleosomal DNA can be bound. However, we find that this ability requires interactions with inter-nucleosomal linker DNA. Nucleosome core DNA methylation reading involves regions of MeCP2 beyond its canonical methyl-CpG binding domain and we define a novel DNA-binding region in MeCP2 that is required for this function. We further demonstrate that histone H1 antagonises the MeCP2-nucleosome interactions by competing for linker DNA. Overall, our study reveals that MeCP2 gains access to methylated chromatinised DNA, independent of nucleosome structure, via essential nonspecific interactions with linker DNA.

## Abstract (web summary)

Watson et al., explored how Rett syndrome protein MeCP2 interacts on chromatin. They showed MeCP2 can unexpectedly interact with nucleosome-core buried DNA methylation, requiring cooperative binding of known and newly identified DNA binding regions.

## Introduction

Methyl-CpG-binding protein 2 (MeCP2) is an epigenetic reader of cytosine methylation (meC), which regulates gene expression^1–10^. MeCP2 is highly abundant in neurons^11^ and essential for normal neuronal maturation and function^12,13^. Indeed, heterozygous mutations cause Rett syndrome^4,14–16^, a severe neurological disorder affecting around 1 in 10,000 live female births^17^. Mouse and cellular models have helped to explain the pathophysiology of Rett syndrome^12,18–20^, but a complete molecular explanation of how this leads to the observed disease phenotypes is lacking.

MeCP2 modulates gene expression and genome architecture via its recruitment to chromatin and its association with additional factors^18^. MeCP2 binds chromatin pervasively in the genome through a multifaceted, context-dependent mechanism that is not fully understood^21^. There is evidence that this is guided by the epigenome through DNA methylation recognition^7,11,22–24^, although additional DNA binding specificities have been proposed. MeCP2 contains only a single folded domain, the methyl-binding domain (MBD), that binds DNA cytosine methylation (meC). Upwards of 80% of meCpG sites in the genome are methylated, a number that is amplified further in neurons due to abundant asymmetric meCpA modifications, both of which can be engaged by MeCP2’s MBD^1,5,6,22,24–31^. In vitro, the highly basic and disordered protein also robustly binds unmethylated double-stranded DNA, with a weaker preference for methylated DNA than the MBD alone^28,32–34^. Outside of the MBD, DNA binding activities have also been identified throughout MeCP2, which have been proposed to contribute to the cooperative DNA binding of the protein^1,35–42^.

MeCP2 binding occurs within chromatin rather than naked DNA. The nucleosome is the fundamental unit of chromatin and comprises an octameric core of histones that wrap and compact ~145–147 bp of DNA, punctuated by linear linker DNA between consecutive nucleosome core particle units^43^. Previous in vitro studies of MeCP2 interacting with nucleosomes have proposed that MeCP2 can bind meCpG on solvent-facing nucleosomal major grooves^44,45^. MeCP2 also reportedly binds both unmethylated and methylated nucleosomes^45–48^, with different behaviours on chromatin versus linear DNA^49^. Histone proteins and histone tail modifications have additionally been suggested as potential targets for MeCP2^46,49–53^. Indeed, in cells MeCP2 binds in a meC-specific and non-specific manner^9,11,23,34^, but the molecular basis for full-length MeCP2 interaction with chromatin remains unclear.

We sought to better understand the binding mode of MeCP2 to nucleosomal DNA methylation. Single sites of DNA methylation were engineered throughout the nucleosome, using both meCpG and meCpA sequences. We found that MeCP2 can bind DNA methylation on the nucleosome surface, even when meC sites are facing the histone octamer, which would be predicted to block binding. This unanticipated binding was dependent on the presence of accessible nucleosomal linker DNA and required a central region of MeCP2 outside of the previously characterised domains. Accordingly, MeCP2 achieved minimal binding to nucleosome core particles lacking linker DNA and H1-bound chromatosomes with short linker DNA. Additionally, mutation of this novel DNA-binding region disrupted meC specificity in vitro and altered MeCP2 binding dynamics to heterochromatin in vivo. Our findings suggest that the absence of the central domain in truncated forms of MeCP2^54^ hinders access to sites of DNA methylation on the nucleosome surface, and may therefore limit functionality.

## Results

### Full-length MeCP2 is required to bind nucleosomal DNA methylation

We created site specifically-modified designer nucleosomes to probe the interaction of MeCP2 on methylated DNA at specific nucleosome locations. Use of strong positioning DNA sequences^55^ allowed us to precisely situate the histone octamer core with defined lengths of DNA linker regions. We utilised methyltransferase recognition sequences to install site-specific cytosine CpG DNA methylation (meC) in either the linkers or core-binding DNA regions (Supplementary Fig. 1A), which could then be wrapped into nucleosomes (Supplementary Fig. 1B, C). Nucleosomes were well wrapped with characteristic periodicity observed by hydroxyl radical footprinting (Supplementary Fig. 1D).

The methylated DNA binding domain (MBD) of MeCP2 is critical to promote interaction with methylated cytosines (meC) in linear DNA^1,5,6,22,24–31^. We first sought to determine if the MBD in isolation was sufficient to allow meC reading in the context of nucleosomes. Using purified recombinant MeCP2 MBD (residues 77–167, Supplementary Fig. 2A), we tested the interaction by electrophoretic mobility shift assay (EMSA) with nucleosomes containing unequal length 37 bp and 27 bp DNA linker arms and a core sequence of Widom 601 DNA (termed 37-N_601_-27). Due to linker DNA sequences also containing methyltransferase recognition sequences, nucleosomes were either triple methylated (+83, −80 and −61 bp from the central dyad axis of the nucleosome core), or single methylated (−1 bp from the dyad) (Supplementary Fig. 1A). An unlabelled double stranded DNA competitor was included in all assays to limit non-specific binding for methylation specificity (Supplementary Fig. 2B), as described previously^45,46^.

**Fig. 1:**
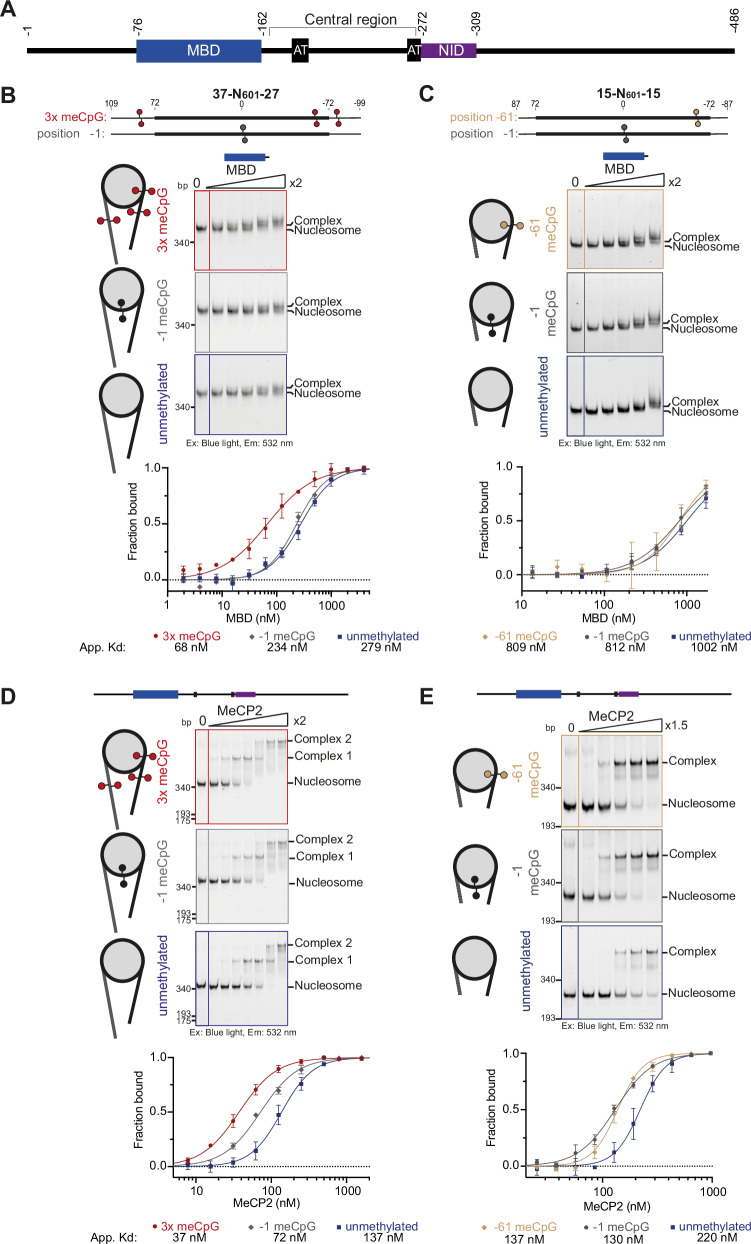
Full-length MeCP2 is required to bind nucleosomal DNA methylation. **A** Diagram of full-length human e2 MeCP2 highlighting the key methyl binding domain (MBD) (blue box) and NCoR/SMRT interaction domain (NID) (purple box). Domain boundaries are indicated with residue number. AT hook motif (AT) 1 and 2 are also shown (black boxes). **B** Representative EMSA native-PAGE (3 repeats) showing a 2-fold dilution series of MBD (residues 77–167) with limiting amounts (2.5 ng) of H2B T115C-OregonGreen488 labelled 37-N_601_-27 nucleosomes. Concentrations 15.6–250 nM on the gel are shown for clarity. Nucleosomes were either methylated with meCpG at three positions (+83, −61, −80 bp from the dyad) (red), a single meCpG (−1 bp from the dyad) (grey), or unmethylated (blue). Free nucleosome and complex bands are indicated, size markers in bp are shown. Quantification of the free nucleosome bands at each concentration of the full concentration series (1.95–4000 nM), was fitted with a binding isotherm, and an apparent dissociation constant (*K*_D app_) calculated. Error bars show standard error of the mean. Calculated K_D app_ and hill slope statistics are summarised in Table 1. **C** Representative EMSA native-PAGE (3 repeats) showing a 2-fold dilution series of MBD (residues 77–167) with limiting amounts (2.5 ng) of H2B T115C-OregonGreen488 labelled 15-N_601_-15 nucleosomes. Concentrations 107–1713 nM on the gel are shown for clarity. Nucleosomes were methylated with a single meCpG either −61 bp (brown) or −1 bp (grey) from the dyad, or unmethylated (blue). Free nucleosome and complex bands are indicated. Quantification of the full concentration series (6.7–13,707 nM) was performed as described in (**B**). **D** Representative EMSA native-PAGE (3 repeats) showing a 2-fold dilution series of full-length MeCP2 with 37-N_601_-27 nucleosomes described in (**B**). Concentrations 15.6–1000 nM on the gel are shown for clarity. Quantification of the full concentration series (3.9–2000 nM) was performed as described in (**B**). **E** Representative EMSA native-PAGE (3 repeats) showing a 1.5-fold dilution series of full-length MeCP2 on 15-N_601_-15 nucleosomes described in C. Concentrations 85–430 nM on the gel are shown for clarity. Quantification of the full concentration series (11–968 nM) was performed as described in (**B**). Source data are provided as Source Data Files 1 and 2.

As expected, the MBD robustly bound to nucleosomes containing multiple meC sites in the linear linker DNA projecting away from the nucleosome (−80, −61 and 83 bp from the centre point of the nucleosome or dyad; Fig. 1B), with reduced binding to non-methylated nucleosomes. However, placing a single meC site in the extensively bent, histone-contacting nucleosome core (−1 bp from the dyad), no longer allowed methyl-specific binding of the MBD (Fig. 1B) Similarly, shorter-linker nucleosome substrates (15-N_601_-15), with no additional linker meCpG sites, lacked methylation preference when a meC was placed near the dyad (Fig. 1C). This phenomenon was not due to the exact positioning of meC, as moving the meC closer to the entry/exit DNA site of the nucleosome core (position −61, Fig. 1C) also blocked binding. We conclude that methylated DNA sites associated with the nucleosome core are refractory to meC binding by the MBD, likely due to steric occlusion from the histones contacting the bent core of nucleosome DNA (Supplementary Fig. 2C, D).

**Fig. 2:**
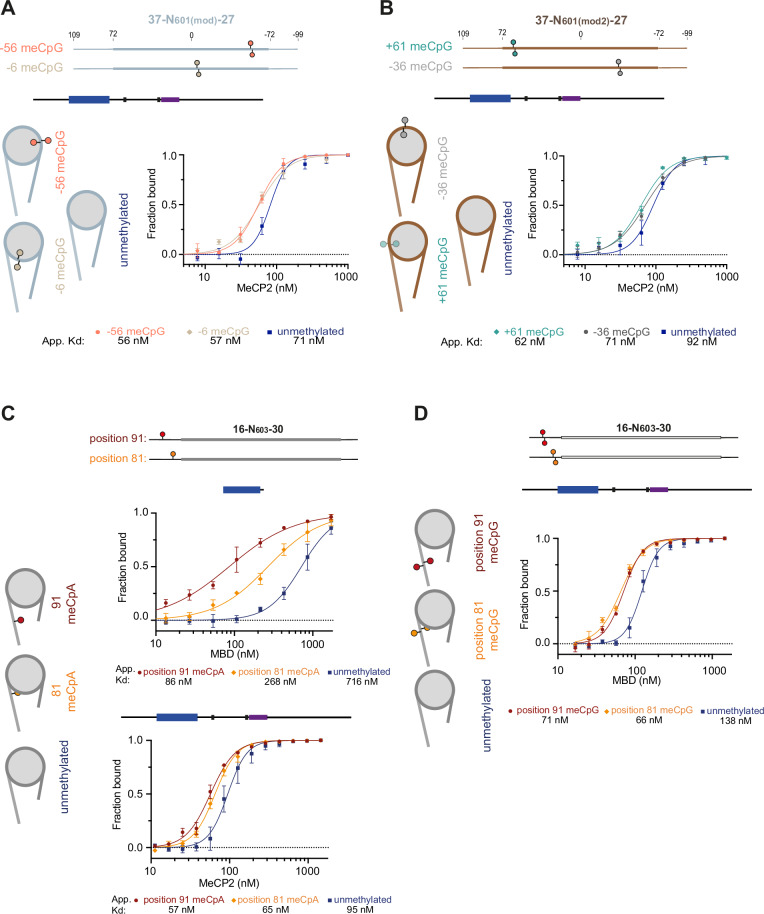
MeCP2 binds preferentially to DNA methylation around the nucleosome. **A** Quantification of EMSA native-PAGE experiments (3 repeats) (Supplementary Fig. 4B) showing a 2-fold dilution series of MeCP2 with limiting amounts (2.5 ng) of 5’ 6-FAM labelled 37-N_601(mod)_−27 nucleosomes. The core Widom 601 sequence was altered to accommodate specific single meCpG positioning. Nucleosomes were methylated with meCpG either at position −6 (light brown) or −56 (orange), or unmethylated (blue). The full concentration series (1–2000 nM), was fitted with a binding isotherm and an apparent dissociation constant (*K*_D app_) calculated. Error bars show standard error of the mean. Calculated *K*_D app_ and hill slope statistics are summarised in Table 1. **B** Quantification of EMSA native-PAGE experiments (3 repeats) (Supplementary Fig. 4C) showing a 2-fold dilution series of MeCP2 with limiting amounts (2.5 ng) of 5’ 6-FAM labelled 37-N_601(mod2)_−27 nucleosomes. The core Widom 601 sequence was altered to accommodate specific single meCpG positioning. Nucleosomes were methylated with meCpG either at position −36 (light grey) or +61 (teal), or unmethylated (blue). The full concentration series (1–2000 nM), was fitted with a binding isotherm as described in (**A**). **C** Quantification of EMSA native-PAGE experiments (Supplementary Fig. 5B) showing a 2-fold dilution series of MBD (3 repeats), and a 1.5-fold dilution series of MeCP2 (4 repeats), with limiting amounts (2.5 ng) of H2B T155C-OregonGreen488 labelled 16-N_603_-30 nucleosomes. Nucleosomes were either methylated with meCpA at position 91 (red), position 81 (orange), or unmethylated (blue). The full concentration series (MBD: 6.7–27413 nM, MeCP2: 11–1253 nM), was fitted with a binding isotherm as described in (**A**). **B** Quantification of EMSA native-PAGE experiments (3 repeats) (Supplementary Fig. 5C) showing a 1.5-fold dilution series of MeCP2 with limiting amounts (2.5 ng) of H2B T155C-OregonGreen488 labelled 16-N_603_-30 nucleosomes. Nucleosomes were either methylated with meCpG at position 91 (red), position 81 (orange), or unmethylated (blue). The full concentration series (11–1253 nM) was fitted with a binding isotherm as described in (**A**). Source data are provided as Source Data File 1.

Surprisingly, in contrast to the MBD alone, full-length MeCP2 was indifferent to meC positioning on a nucleosome. We first determined that purified MeCP2 (Supplementary Fig. 2A) preferentially recognised meC on histone-free methylated DNA (Supplementary Fig. 2E)^1,33,56,57^. When this DNA was wrapped into nucleosomes, methylation could be engaged when it was present on the linkers, but also when meC was within the DNA associated with the nucleosome core (Fig. 1D). Shorter DNA linker lengths of 15-N601-15 retained the preference of MeCP2 for nucleosomes containing single DNA methylation sites near the dyad or closer to the entry exit site (Fig. 1E). Similarly, moving the DNA methylation to other sites with altered local sequence and histone contacts within the core of the nucleosome preserved the preference of MeCP2 for methylation (Fig. 2A, B and Supplementary Fig. 4). Intriguingly, meC at these sites is predicted to be more accessible than previous sites, which were octamer facing, yet no improvement in the methylation preference of MeCP2 was observed (Fig. 2A, B, Supplementary Figs. 2C and 4A). It is possible that the dynamic, flexible nature of the DNA-bound nucleosome interaction could allow DNA breathing, DNA structure perturbation and/or DNA translocation for meC engagement^58–62^.

Using nucleosomes containing only a single site of DNA methylation on one linker, wrapped with a different core sequence (based on the Widom-603 sequence termed 16-N_603_-30; Fig. 2C and Supplementary Fig. 5), allowed preferential binding by the MBD. However, the affinity was reduced when meC was positioned close to the nucleosome-core (Fig. 2C and Supplementary Fig. 5B), suggesting a similar block as seen for nucleosome core DNA methylation. Unlike the MBD, full-length MeCP2 again displayed similar binding to a single linker meC irrespective of its proximity to the core of the nucleosome (Fig. 2C and Supplementary Fig. 5B). Swapping asymmetric meCpA sites for meCpG also yielded comparable DNA methylation specificity (Fig. 2D and Supplementary Fig. 5C). In combination (Table 1), these data indicate that meC nucleosome recognition at multiple sites and multiple sequence contexts by the MBD is limited when in close proximity to the nucleosome core, but not so when in the context of the full-length protein.

**Table 1:** Summary of affinity measurements assessed by EMSA assays

Protein construct	DNA length	Methylation state	*K*_D app_			*h*		
MeCP2	37-N_601_-27	3x meCpG	37.5	±	1.5	1.7	±	0.1
	37-N_601_-27	−1 meCpG	72.1	±	3.2	1.8	±	0.1
	37-N_601_-27	Unmethylated	137.4	±	6.4	2.1	±	0.2
	37-N_601(mod)_−27	−56 meCpG	55.8	±	2.7	2.6	±	0.3
	37-N_601(mod)_−27	−6 meCpG	57.4	±	2.5	2.1	±	0.2
	37-N_601(mod)_−27	Unmethylated	82.1	±	3.4	3.6	±	0.4
	37-N_601(mod2)_−27	61 meCpG	62.0	±	3.0	2.2	±	0.2
	37-N_601(mod2)_−27	−36 meCpG	70.8	±	5.0	2.0	±	0.2
	37-N_601(mod2)_−27	Unmethylated	91.9	±	5.4	2.8	±	0.4
	15-N_601_-15	−1 meCpG	130.1	±	3.3	2.6	±	0.1
	15-N_601_-15	−61 meCpG	137.4	±	3.8	3.7	±	0.3
	15-N_601_-15	Unmethylated	220.5	±	7.5	3.7	±	0.4
	16-N_603_-30	91 meCpA	57.1	±	1.4	2.8	±	0.2
	16-N_603_-30	81 meCpA	65.3	±	3.3	3.1	±	0.4
	16-N_603_-30	Unmethylated	94.9	±	2.7	3.6	±	0.3
	16-N_603_-30	91 meCpG	71.3	±	1.4	3.5	±	0.2
	16-N_603_-30	81 meCpG	65.5	±	1.6	2.9	±	0.2
	16-N_603_-30	Unmethylated	137.7	±	4.0	3.4	±	0.3
MBD	37-N_601_-27	3x meCpG	67.9	±	4.6	1.0	±	0.1
	37-N_601_-27	−1 meCpG	233.7	±	9.9	1.6	±	0.1
	37-N_601_-27	Unmethylated	278.6	±	17.9	1.5	±	0.1
	15-N_601_-15	−1 meCpG	812.5	±	55.7	1.6	±	0.1
	15-N_601_-15	−61 meCpG	808.9	±	56.2	1.9	±	0.2
	15-N_601_-15	Unmethylated	1001.9	±	62.9	1.6	±	0.1
	16-N_603_-30	91 meCpA	85.7	±	6.1	1.0	±	0.1
	16-N_603_-30	81 meCpA	267.6	±	17.3	1.3	±	0.1
	16-N_603_-30	Unmethylated	715.9	±	38.3	2.0	±	0.2

Using full-length MeCP2 increased overall affinity for meC nucleosomes, implying additional binding capability. However, MeCP2 also reduced the selectivity for meC compared to the isolated MBD, suggesting this added affinity is not DNA methylation sensitive. MeCP2 contains multiple regions previously attributed to DNA binding outside of the MBD^1,35–42^. Indeed, the binding is likely multivalent as evidenced by Hill coefficients indicative of multiple sites of cooperative binding. Interestingly, MeCP2 displayed a generally stronger binding affinity to nucleosomes containing CpA sequences over CpG, independent of methylation, suggesting a potential sequence preference^34^. This was not observed for binding to the unwrapped DNA (Supplementary Fig. 5A). Similarly, MeCP2 has been shown to have a degree of sequence specificity^34,63^. However, methylation association within the nucleosome core, at multiple sites and sequence contexts, was observed, suggesting this is a generalisable feature of MeCP2 interaction on chromatin.

In all cases, MeCP2-nucleosome complexes migrated as distinct bands. On longer nucleosome substrates, a second slower migrating band was also observed at high MeCP2 concentrations, suggesting a second binding event could be accommodated by increased DNA linker length (Fig. 1D). Mass photometry analysis of complexes confirmed the presence of one and two MeCP2 binding events to the multiply methylated 37-N_601_-27 nucleosomes. A single binding event was observed in 15-N_601_-15 nucleosomes, in agreement with the number of meC available (Supplementary Fig. 3). Taken together, our results suggest that meC recognition within nucleosome cores by MeCP2 requires regions in addition to the MBD.

### MeCP2 requires linker DNA to bind meC sites in core nucleosomal DNA

Full-length MeCP2 displayed higher binding affinity to both meC- and non-methylated nucleosomes compared to the MBD alone. We first tested if this was due to histone protein interactions^46,49–53^. Crosslinking mass spectrometry shows links between the MBD and N-terminal tail of H3 (Supplementary Fig. 6A). However, the H3 tail is adjacent to the engineered sites of meC, and shifting DNA methylation into the core of the nucleosome also varied the protein-protein crosslinking pattern, reflecting the location of meC rather than a consistent feature of MeCP2 binding. Removing the tail of H3 or addition of H3K_c_27me3 - which has been previously suggested to be a binding target of the MBD^51,52,64^, as well as antagonise MeCP2-histone binding^53^ - did not affect MBD binding to nucleosomes in these assays (Supplementary Fig. 6B). Indeed, removal of the H3 tail globally increased MeCP2 binding to nucleosomes (Supplementary Fig. 6C). The H3 tail is known to bind linker DNA^65,66^, raising the possibility that the histone tail competes with a MeCP2-linker binding that is important for MeCP2 interaction with the nucleosome.

**Fig. 3:**
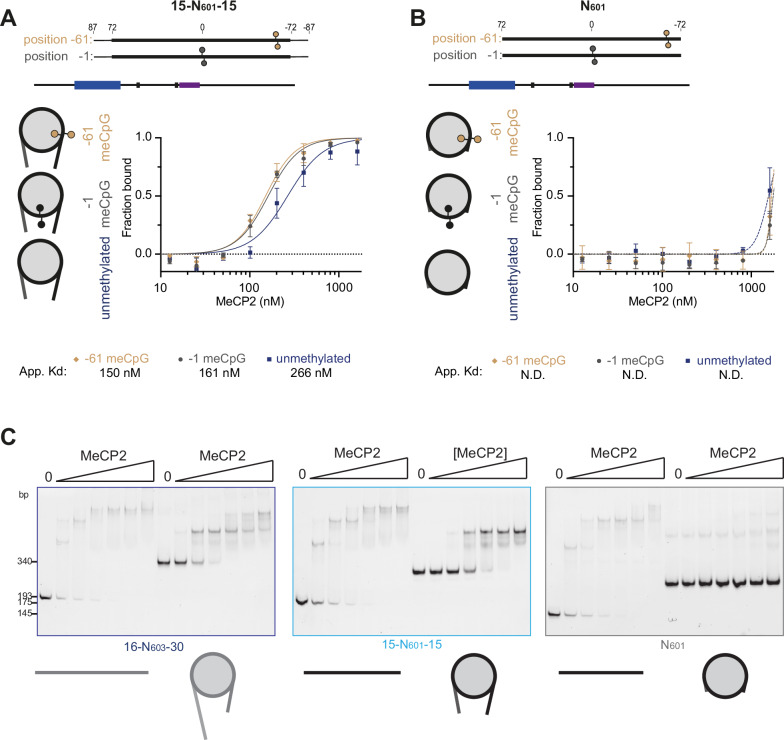
Linker DNA is essential for MeCP2 to bind nucleosomal DNA methylation. **A** Quantification of EMSA native-PAGE experiments (3 repeats) (Supplementary Fig. 7A) showing a 2-fold dilution series of MeCP2 with limiting amounts (2.5 ng) of H2B T155C-OregonGreen488 labelled 15-N_601_-15 nucleosomes. Nucleosomes were methylated with meCpG either at position −1 (grey) or −61 (brown), or unmethylated (blue). The full concentration series (3.2–12912 nM), was fitted with a binding isotherm and an apparent dissociation constant (*K*_D app_) calculated. Error bars show standard error of the mean. Calculated *K*_D app_ and hill slope statistics are summarised in Supplementary Table 3. **B** Quantification of EMSA native-PAGE experiments (3 repeats) (Supplementary Fig. 7A) showing a 2-fold dilution series of MeCP2 with limiting amounts (2.5 ng) of H2B T155C-OregonGreen488 labelled N_601_ nucleosome core particles. Nucleosomes were methylated with meCpG either at position −1 (grey) or −61 (brown), or unmethylated (blue). The full concentration series (6.3–12912 nM), was fitted with a binding isotherm as described in (**A**). **C** Summary EMSA native-PAGE showing a 2-fold dilution series (31.3–1000 nM) of MeCP2 with limiting amounts (2.5 ng) of H2B T115C-OregonGreen488 labelled 16-N_603_-30 (dark blue), 15-N_601_-15 (light blue) and N_601_ (grey) nucleosomes, alongside the corresponding 5’ 6-FAM labelled bare DNA. Binding to DNA is shown on the left of each gel, nucleosomes to the right. Size markers in bp are shown. Binding to each component was at least repeated in duplicate. Source data are provided as Source Data Files 1 and 2.

To test the hypothesis that MeCP2 binding to DNA on the nucleosome surface requires linker DNA, we assayed the MeCP2 interaction with linker-less nucleosomes. Strikingly, we found that removal of linker DNA to form nucleosome core particles (N_601_) disrupted binding of MeCP2, with or without meC (Fig. 3A, B and Supplementary Fig. 7A). This linker requirement was independent of DNA methylation. As longer linker lengths enhanced binding of full-length MeCP2 (Fig. 1D, E), we therefore hypothesised that additional DNA binding aids MBD-meC chromatin interaction. MeCP2 contains appreciable non-specific DNA binding ability, which drives dynamic association with the genome^39,67^. Indeed, direct comparison of MeCP2 binding to DNA of various lengths, either as wrapped nucleosomes or unwrapped free DNA, highlights its binding preference for linear double-stranded DNA (Fig. 3C).

To examine the minimal amount of linker DNA required for MeCP2-nucleosome binding, asymmetric nucleosomes with only a single linker were tested for MeCP2 interaction. The presence of a single 15 bp linker (N_601_-15) led to overall poorer binding, reduced Hill slope indicating less cooperativity and a loss of meC specificity compared to 15-N_601_-15 (Supplementary Fig. 7B and Table 1). This result suggests that a binding interface has been removed. Doubling the length of the single linker (N_603_-30) restored both DNA binding and meC specificity to the levels seen with 16-N_603_-30 (Supplementary Fig. 7C), suggesting either the single 30 bp linker, or two adjacent 15 bp linkers, was sufficient to maintain optimal MeCP2 binding to nucleosomes accommodating both the MBD and any additional DNA interacting region of MeCP2 (Supplementary Fig. 7D). In the absence of competitor DNA the MBD-alone and MeCP2 can interact with nucleosome core particles, but in a non-methylation sensitive manner (Supplementary Fig. 8A, B). This suggests that the MBD of full-length MeCP2 can no longer engage robustly without the stability conferred by additional DNA-binding elements, and MeCP2 combines meC-MBD and DNA linker interaction.

### A central region of MeCP2 binds nucleosome linker DNA

**Fig. 4:**
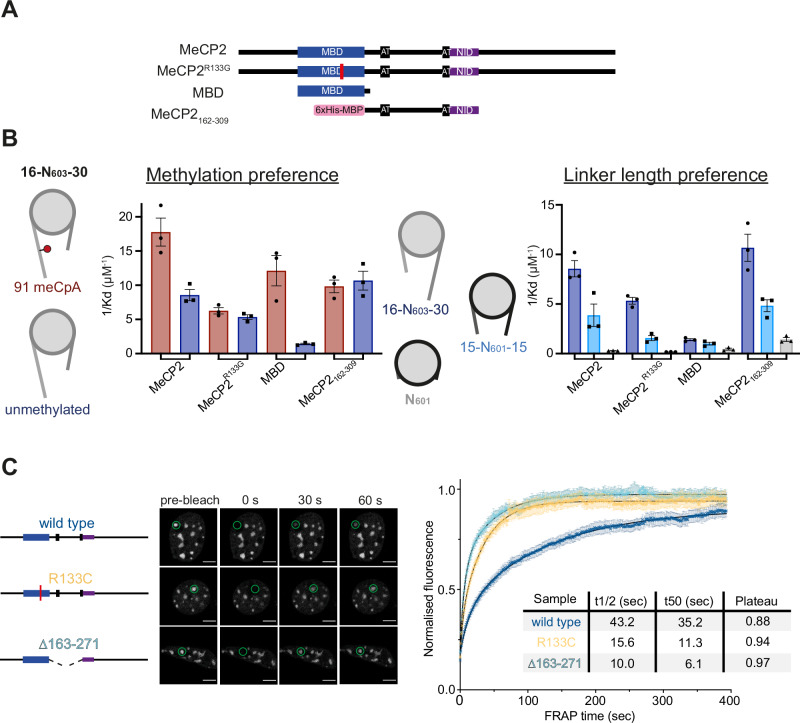
A central region of MeCP2 binds nucleosome linker DNA. **A** Diagram of MeCP2 constructs used in part (**B**). The domains of MeCP2 and tags present in each construct is highlighted. MBD mutation R133G location is marked by a red line. **B** Summary of MeCP2 variant affinity measurements determined by EMSA (3 repeats). (Left) Methylation preference was assayed by comparison of binding affinities for each construct on 16-N_603_-30 nucleosomes, with either 91 meCpA (red, circles) or unmethylated (blue, squares). (Right) Linker length preference by comparison of affinities on unmethylated 16-N_603_-30 (dark blue, circles), 15-N_601_-15 (light blue, squares) and N_601_ (grey, triangles) nucleosomes. Each data set shows the inverse apparent dissociation constants (1/Kd) calculated for individual repeats. Error bars represent the standard error of the mean between 1/Kd values. Examples of EMSA native-PAGE gels used are shown in Supplementary Fig. 9A, B. **C** FRAP quantification of wild-type (blue), ∆163–271 (cyan) and R133C (yellow) eGFP-MeCP2 recovery in mouse fibroblasts. The number of analysed cells, from 3 independent experiments, are: WT *n* = *37* cells, ∆163–271 *n* = 33 cells, R133C *n* = 28. Error bars show SEM. An example time-course from the live cell imaging of each construct is also shown, with the bleached foci circled. Source data are provided as Source Data Files 1.

The data above suggest that DNA binding capability outside of the MBD is required for MeCP2 to gain access to meC in a nucleosome core. We therefore set out to identify which regions of MeCP2 are involved in nucleosome linker binding. Crosslinking mass spectrometry suggested a central region of MeCP2 is adjacent to the nucleosome (Supplementary Fig. 6A). However, the histone acidic patch, a common site of chromatin-protein engagement^68^, was not required for the interaction in this context (Supplementary Fig. 8A–E). The central region is sometimes referred to as the Intervening Domain (ID) and Transcriptional Repression Domain (TRD), and contains reported DNA binding activity. This is in part ascribed to AT-hooks 1 and 2^1,35–37,39–42,69^. A construct covering this central region (residues 162–309) bound poorly to nucleosome core particles (N_601_), but better to nucleosomes with linkers (15-N_601_-15, Fig. 4A, B and Supplementary Fig. 9A, B). Comparing the binding of this central region construct with that of MeCP2 or the MBD alone revealed that the full-length MeCP2 interaction could be explained by a combination of both meC-specific and linker DNA-binding modalities. While full-length MeCP2 preferentially binds methylated DNA, it shows appreciable affinity for unmethylated nucleosomes (Fig. 4A, B). Methylation preference is driven by the MBD: incorporation of a mutation that ablates meC recognition (R133G; Supplemental Fig. 9D) removes discrimination between meC and unmethylated nucleosomes, and the MBD alone has very low affinity to unmethylated nucleosomes. Increasing the length of nucleosome linker DNA improves MeCP2 interaction, as seen previously (Supplementary Fig. 7). Notably, the central region shows no methylation-dependent binding preference, but has a clear increased affinity for longer linkers (Fig. 4A, B). The data are compatible with the hypothesis that this region engages linker DNA and combines with the MBD to promote meC-nucleosome recognition.

To test the importance of the central region for MeCP2 function, we performed fluorescence recovery after photobleaching (FRAP) experiments in NIH3T3 mouse fibroblast cells (Fig. 4C, Supplementary Fig. 14). As seen in previous studies, wild-type eGFP-tagged MeCP2 localised to highly methylated pericentric heterochromatic foci and, after bleaching undergoes slow and incomplete recovery, suggesting a stably bound fraction^22,63,69–71^. Deletion of MeCP2 163–271, covering the central region, resulted in a more complete and rapid recovery, similar to a known Rett syndrome mutation in the MBD (R133C). This suggests that this region is needed for MeCP2 to stably associate with chromatin in vivo.

### A novel DNA-interacting motif contributes to linker DNA binding activity in central MeCP2

**Fig. 5:**
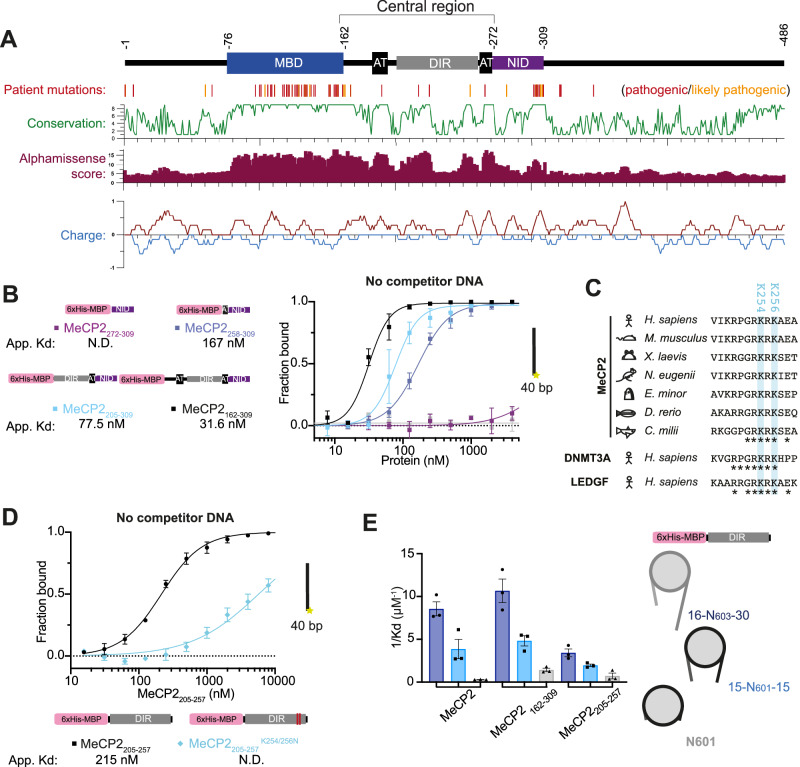
Linker DNA binding occurs through multiple motifs in the central region of MeCP2, including a novel DNA-interacting region. **A** Schematic summarising bioinformatic analysis of MeCP2. Pathogenic (red) and likely pathogenic (orange) point mutations (Clinvar) are plotted along the length of MeCP2. Sequence conservation scores (green), summed AlphaMissense pathogenicity scores (purple), and charge (red-blue) were also plotted. **B** Quantification of EMSA native-PAGE experiments (3 repeats) (Supplementary Fig. 10B) showing a 2-fold dilution series of each HisMBP tagged MeCP2 constructs (272–309 purple, 258–309 blue, 205–309 light blue, 162–309 black, HisMBP control grey) with limiting amounts (2.5 ng) of 5’ 6-FAM labelled 40 bp dsDNA. The full concentration series (272–309, 258–309, HisMBP control: 15.6-32000 nM) (205–309: 7.8–16000 nM) (162–309: 3.9–8000 nM) was fitted with a binding isotherm and an apparent dissociation constant (*K*_D app_) calculated. Error bars show the standard error of the mean. Calculated *K*_D app_ and hill slope statistics are summarised in Supplementary Table 3. No competitor DNA was used in this assay. **C** Multiple sequence alignments of MeCP2_250–256_, from the DNA-interacting region of *Homo sapiens* MeCP2, compared to a variety of animal species. The two lysine residues mutated in the experiments are highlighted in blue. A similar motif was also identified in *H. sapiens* DNMT3A and LEDGF, which are also shown in the alignment. **D** Quantification of EMSA native-PAGE experiments (3 repeats) (Supplementary Fig. 10F) showing a 2-fold dilution series of HisMBP-MeCP2_205–257_ wild-type (black) or mutant (K254/256 N) (light blue) with limiting amounts (2.5 ng) of 5’ 6-FAM labelled 40 bp dsDNA. The full concentration series (15.6–32000) was fitted with a binding isotherm as described in (**B**). Error bars show the standard error of the mean. No competitor DNA was used in this assay. **E** Summary of HisMBP-MeCP2_205–257_ affinity measurements determined by EMSA on unmethylated 16-N_603_-30 (dark blue, circles), 15-N_601_-15 (light blue, squares) and N_601_ (grey, triangles) nucleosomes, as in Fig. 4B (3 repeats). Each data set shows the inverse apparent dissociation constants (1/Kd) calculated for individual repeats. Error bars represent the standard error of the mean between 1/Kd values. Examples of EMSA native-PAGE gels used are shown in Supplementary Fig. 9C. Source data are provided as Source Data Files 1.

Primary sequence analysis of MeCP2 highlighting pathogenic and likely-pathogenic point mutations^72^, sequence conservation^73^, and predicted pathogenicity using AlphaMissense^74,75^, all reveal the crucial role of the MBD and NCoR/SMRT interacting domain (NID, Fig. 5A). In addition, the central region between the MBD and NID domains contains several neurodevelopmental-implicated patient mutations corresponding to the AT-hooks 1 and 2, as well as a highly-conserved and invariant uncharacterised region between residues 205–257^16,72,76,77^.

Using this information, we tested the DNA-binding capacity of the central region of MeCP2 using a series of additive purified central region constructs (Supplementary Fig. 10A) in EMSA assays (Fig. 5B and Supplementary Fig. 10B). MeCP2_272–309_, covering only the NID of MeCP2, did not bind to DNA at the concentrations used, similar to a His-MBP tag control (Fig. 5B and Supplementary Fig. 10B, C). The addition of the region containing AT-hook 2 (MeCP2_258–309_) induced DNA binding activity. Extending a further 53 amino acids N-terminal to include the newly identified region (MeCP2_205–309_) further improved binding. This suggests that this region - hereafter termed the DNA Interacting Region, DIR—also contributes to MeCP2 DNA binding activity. Finally, extension to include AT-hook 1 (MeCP2_162–309_) showed the most robust DNA binding activity, indicating that three central MeCP2 DNA-binding regions work together to engage DNA. Indeed, addition of AT-hooks and the DIR increased not only binding affinity but also the Hill slope (Fig. 4 and Supplementary Table 3).

A small construct containing only the DIR (MeCP2_205–257_, Supplementary Fig. 10D) was sufficient to bind double-stranded DNA (Fig. 5D and Supplementary Fig. 10E), with a preference for longer lengths of DNA (Supplementary Fig. 10F) and no observable DNA sequence preference (Supplementary Fig. 11A). The DIR fragment appears as a monomer in isolation and likely exists as an extended non-globular structure (Supplementary Fig. 11B). The DIR is not overall strongly positively charged (Fig. 5A) but includes a conserved motif ‘R-P-G-R-K-R-K’ (residues 250–256, Fig. 5C). Mutation of two lysine residues (K254N, K256N) significantly reduced DNA binding (Fig. 5D and Supplementary Fig. 10E), suggesting that this motif is principally responsible for DNA binding activity of the DIR. Interestingly an identical short amino acid sequence is found in the de novo methyltransferase DNMT3A, and a similar motif is also present in Lens Epithelium-Derived Growth Factor (LEDGF/p75) (Fig. 5C). In both cases the regions have been implicated in DNA binding^78,79,80^. Indeed, we observed that mutation of the motif in a DNMT3A construct also reduced DNA binding (Supplementary Fig. 11C). Both DNMT3A^80^ and LEDGF^81^ also engage with nucleosomes, raising the possibility that this motif could be a generalisable DNA binder that promotes multivalent chromatin interactions. Compatible with this notion, we found that the DIR also bound to nucleosomes with a preference for longer linker DNA, albeit weaker than the entire central region (Fig. 5E and Supplementary Fig. 9C). Interestingly, the DIR bound nucleosome core particles poorly, suggesting it does not engage with core nucleosomal DNA or the histone acidic patch. Therefore, the behaviour of the DIR engaging linear accessible linker DNA mimics the specificity of intact MeCP2.

### Motifs in central MeCP2 aid MBD binding to nucleosomal DNA methylation

**Fig. 6:**
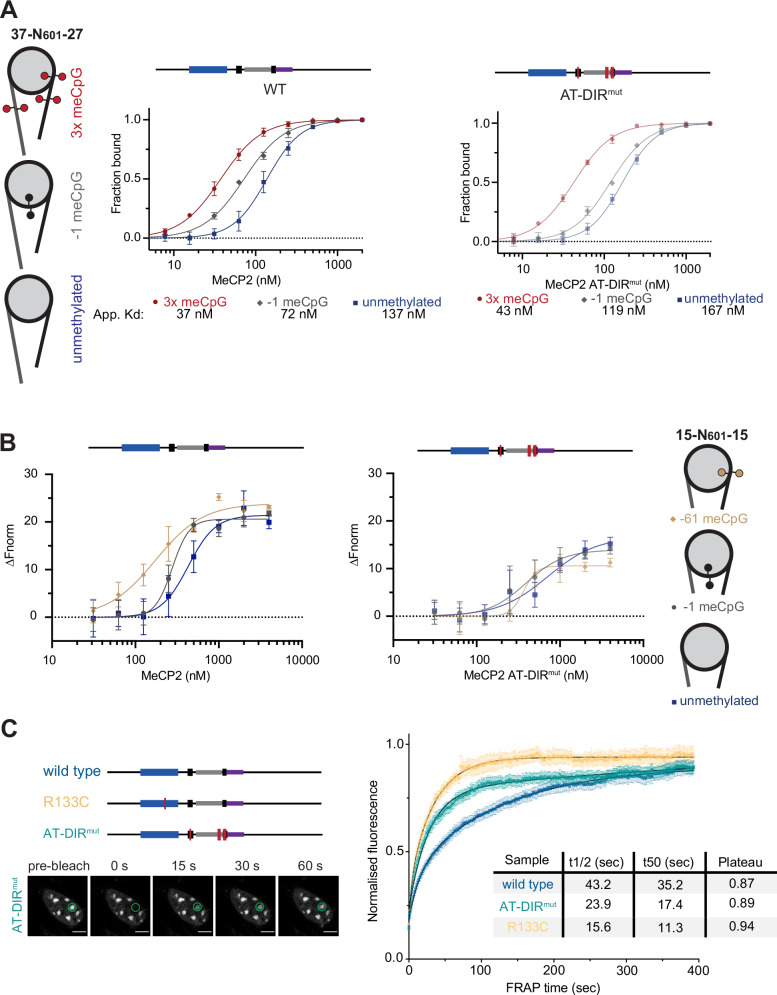
Disruption of DNA-interacting motifs in central MeCP2 limits binding to nucleosomal DNA methylation. **A** Quantification of EMSA native-PAGE experiments (3 repeats) (Supplementary Fig. 12B) showing a 2-fold dilution series of MeCP2 wild-type (left) or AT-DIR^mut^ (R188G, R190G, K254N, K256N and R268Q) (right) with limiting amounts (2.5 ng) of H2B T115C-OregonGreen488 labelled 37-N_601_-27 nucleosomes. WT data were shown previously (Fig. 1D). Nucleosomes were methylated with meCpG at three positions (+83, −61, −80 bp from the dyad) (red), a single meCpG (−1 bp from the dyad) (grey), or unmethylated (blue). The full concentration series (3.9–2000 nM) was fitted with a binding isotherm, and an apparent dissociation constant (*K*_D app_) was calculated. Error bars show the standard error of the mean. Calculated *K*_D app_ and hill slope statistics are summarised in Supplementary Table 3. **B** Amplitude normalised and baseline subtracted MST data (3 repeats) showing a 2-fold dilution series (31.3–4000 nM) of MeCP2 wild-type (left) or AT-DIR^mut^ (R188G, R190G, K254N, K256N and R268Q) (right) on 5 nM of H2A K119C-alexa647 labelled 15-N_601_-15 nucleosomes. Nucleosomes were methylated with a single meCpG either at position −61 bp (brown) or −1 bp (grey), or unmethylated (blue). Error bars show the standard error of the mean. **C** Graph showing the FRAP quantification of wild-type (blue), AT-DIR^mut^ (teal) and R133C (yellow) eGFP-MeCP2 in mouse fibroblasts. Wild-type and R133C are shown as before (Fig. 3B) for comparison. The number of analysed cells, from 3 independent experiments, is: AT-DIR^mut^ *n* = 31 cells. Error bars show SEM. Source data are provided as Source Data Files 1.

If the DNA binding activity of central MeCP2 is necessary to allow binding to meC nucleosomes, we hypothesised that disruption of this region would produce a protein that, like MBD alone, would not be able to access nucleosome core DNA meC. To test this, we purified full-length MeCP2 containing mutations in the DIR region (K254N, K256N) in addition to both AT-hook 1 (R188G, R190G) and 2 (R268Q)^39,63^, to abrogate DNA binding activity in the region (MeCP2 AT-DIR^mut^; Supplementary Fig. 12A). Binding to methylated nucleosomes was assayed in vitro as before by EMSA, as well as by both surface plasmon resonance (SPR) and microscale thermophoresis (MST). Reduced DNA binding capabilities of MeCP2 AT-DIR^mut^ ablated overall binding affinity to all 37-N_601_-27 nucleosomes, as expected (Fig. 6A; Supplementary Figs. 12B and 13A). Additionally, the mutations had an even greater effect when a single meC was positioned in the nucleosome core: meC preference was weaker compared to the retained activity when meC was in distal linker regions. A similar loss of nucleosomal methylation preference for MeCP2 AT-DIR^mut^ was also seen for binding to 15-N_601_-15 nucleosomes with meC confined to the nucleosome core (Fig. 6B; Supplementary Fig. 12C and 13B), as well as to 16-N_603_-30 nucleosomes (Supplementary Fig. 13C). Thus the specificity of full-length MeCP2 with this combination of central region mutations resembles that of the MBD alone, supporting the hypothesis that the central region is responsible for providing access to nucleosomal DNA.

FRAP analysis of the AT-DIR^mut^ construct in live cells confirmed disrupted chromatin binding dynamics. The magnitude of the effect was similar to, although somewhat milder than, the previously observed MeCP2 central deletion (∆163–271) and the Rett syndrome-causing disease mutation control (R133C; Fig. 6C and Supplementary Fig. 14). In all mutant cases, the recovery was more rapid and complete than the wild-type, indicating reduced stability of the MeCP2-chromatin interaction in vivo.

### The C-terminal tail of H1 blocks MeCP2 access to nucleosome linker DNA

**Fig. 7:**
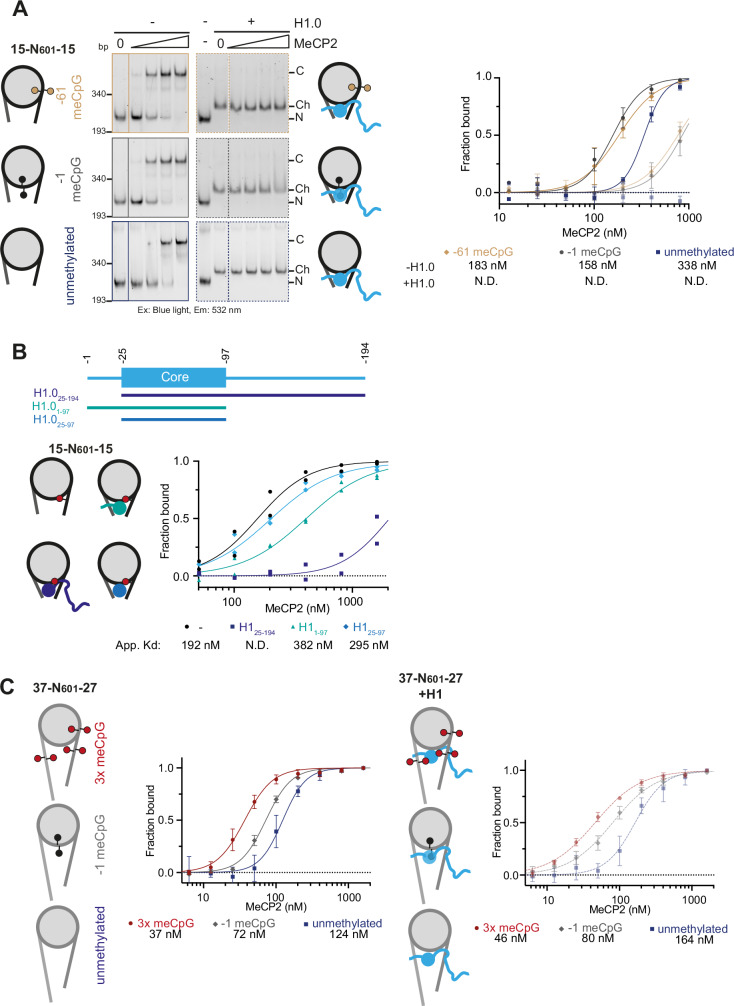
MeCP2 competes with H1.0 for nucleosome linker DNA binding. **A** Representative EMSA native-PAGE (3 repeats) showing a two-fold dilution series of MeCP2 with limiting amounts (2.5 ng) of H2B T115C-OregonGreen488 labelled 15-N_601_-15 nucleosomes (left) or chromatosomes (right). Chromatosomes were formed by pre-incubation with H1.0. Concentrations 50.4–404 nM on the gel are shown for clarity. Nucleosomes were methylated with a single meCpG either −61 bp (brown) or −1 bp (grey) from the dyad, or unmethylated (blue). Free nucleosome (N), chromatosome (Ch), and complex (C) bands are indicated, size markers in bp are shown. Binding isotherms and *K*_D app_ are also shown. Quantification of the free nucleosome bands at each concentration of the full concentration series (6.3–3228 nM) was fitted with a binding isotherm, and an apparent dissociation constant (*K*_D app_) was calculated. Error bars show the standard error of the mean. Calculated *K*_D app_ and hill slope statistics are summarised in Supplementary Table 3. **B** Quantification of EMSA native-PAGE experiments (2 repeats) (Supplementary Fig. 15D) showing a 2-fold dilution series of MeCP2 on limiting amounts (2.5 ng) of H2B T115C-OregonGreen488 labelled 15-N_601_-15 nucleosomes, or chromatosomes assembled with each H1.0 tail deletion construct as indicated. Nucleosomes were methylated with linker meCpA 81 bp from the dyad. The full concentration series (50–6456 nM) was fitted with a binding isotherm as described in A. Individual datapoints at each concentration represent a repeat. **C** Quantification of EMSA native-PAGE experiments (5 repeats) (Supplementary Fig. 15F) showing a 2-fold dilution series of MeCP2 with limiting amounts (2.5 ng) of H2B T115C-OregonGreen488 labelled 37-N_601_-27 nucleosomes. Chromatosomes were formed by pre-incubation with H1.0. Nucleosomes were either methylated with meCpG at three positions (+83, −61, −80 bp from the dyad) (red), a single meCpG (−1 bp from the dyad) (grey), or unmethylated (blue). The full concentration series (3.2–3228 nM) was fitted with a binding isotherm as described in (**A**). Source data are provided as Source Data Files 1 and 2.

Linker histone H1 also binds nucleosome linker DNA and is found at a high concentration in neurons, approximately equivalent to that of MeCP2. The two proteins have been reported to condense chromatin and compete with one another for linker DNA binding^11,45^, although this has been contested^49,82^. We decided to test whether H1 affects MeCP2 binding to nucleosomes using our designed system. A set concentration of linker H1.0, which is the predominant variant in neurons^83,84^, was first incubated with 15-N_601_-15 nucleosomes to form chromatosomes (Supplementary Fig. 15A, B). No binding of MeCP2 to 15-N_601_-15 chromatosomes was observed even in the presence of nucleosome core meC (Fig. 7A), reminiscent of the absence of binding seen to N_601_ nucleosome core particles. Removal of the C-terminal tail of H1.0, which binds linker DNA, mostly restored MeCP2 binding to chromatosomes (Fig. 7B and Supplementary Fig. 15C, D). This suggests that H1 can block meC reading in a nucleosome by masking short DNA linkers from MeCP2.

Interestingly, further lengthening linkers in 37-N_601_-27 nucleosomes allowed for concurrent MeCP2 and H1.0 binding, with MeCP2 binding affinity only slightly lowered on chromatosomes versus nucleosomes (Fig. 7C and Supplementary Fig. 15E–G)^49^. Using intermediate 16-N_603_-30 chromatosomes, with both recombinant purified H1.0 and an ex vivo isolated mixture of H1 isoforms, allowed for some MeCP2 binding, but this was greatly reduced compared to unbound nucleosomes (Supplementary Fig. 16A-D). Overall, this suggests that MeCP2 and H1 compete for proximal, but not distal, nucleosome linker DNA and require long DNA linkers in order to be co-incident.

## Discussion

**Fig. 8:**
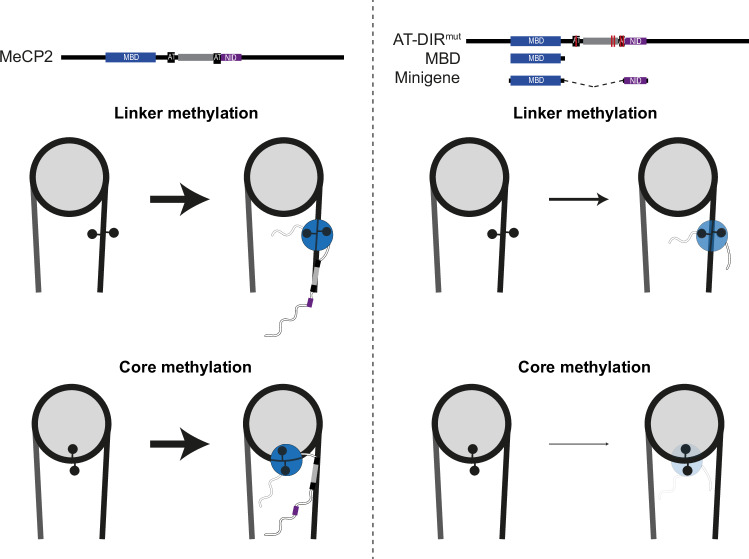
Proposed model for MeCP2 binding to nucleosomal DNA methylation. (Top) Schematics of MeCP2 and variants: MBD, methyl binding domain; AT, AT-hook; DIR, DNA-interacting region. (Middle) DNA methylation on linear linker DNA between nucleosome cores can be bound both by full-length wild-type MeCP2 (left) as well as variants of MeCP2 containing only the MBD, or mutations to the central region of MeCP2 (right). However, overall affinity is reduced in the absence of the central DNA-binding region, which provides additional affinity to dsDNA. When methylation is wrapped into nucleosomes (bottom), it becomes less accessible, and the MBD alone or mutations/loss of the central region is insufficient to bind. Additional linker DNA binding activity from central MeCP2, from two AT-hooks, and a novel DIR is required to allow nucleosomal DNA binding.

MeCP2 has been the subject of intensive study, in part due to the direct genetic link to the relatively common and severe neurological disorder Rett syndrome. Here, we took advantage of site-specific DNA methyltransferases and alternative DNA sequences to specifically engineer single sites of DNA methylation on a nucleosome, with the aim of clarifying disparate models of MeCP2 binding on chromatin. We identified an essential role for nucleosome linker DNA in the overall interaction of MeCP2 on nucleosomes and confirmed that separable meC and DNA binding regions are important for a stable interaction^35^. We observe that, facilitated by these linker DNA interactions, the MBD of MeCP2 can bind DNA methylation throughout the nucleosome (Fig. 8).

The density of meC has a large influence on MeCP2 binding^11,46,85^. Indeed, we see that the highest affinity is achieved when multiple meC are present per nucleosome (Fig. 1). However, CpG sites are under-represented in the genome, with on average 1–2 meC present per nucleosome repeat. MeCP2 levels are high in neurons, which undergo an increase in DNA methylation at non-CpG sites, roughly doubling the overall meC levels in the genome^86^. Our approach of using single meC sites within the nucleosome, therefore, approximates the meC density found in vivo. The relative difference in affinity for meC over unmodified nucleosomes is close to an order of magnitude in the case of the MBD alone, but more modest for the full-length protein, due to the addition of non-specific DNA binding by the central region. We propose that in vivo this can manifest as both DNA methylation co-dependent and independent recruitment of MeCP2, depending on exact chromatin context^9,11,23,34^.

The ability of MeCP2 to bind meC at sites in the nucleosome core was surprising, although previous DNase footprinting had suggested the possibility^44^. Some of the meC-core sites tested are in the major groove facing the histone octamer, which would be expected to occlude MeCP2 binding. Preferential binding to these DNA methylated nucleosomes therefore suggests that the nucleosome does not block access to meC to the MBD of MeCP2. Fluctuation of DNA close to the entry/exit sites on nucleosomes may allow for some DNA unwrapping, and therefore account for the unexpected accessibility of methylated DNA nearby. However, methylation was also placed near the nucleosome dyad, which is expected to be less affected by dynamic transitory unwrapping. A possible explanation is that DNA translocation around the nucleosome facilitates MeCP2 engagement. Indeed, meC has been implicated in directly affecting DNA fluidity on the nucleosome^58–62^. Future structural data would be invaluable in explaining how the MBD can engage with meC within predicted occluded bent core DNA.

Here, we show that MeCP2 binding to nucleosomal DNA methylation was dependent on nucleosome linker DNA (Fig. 3), as previously proposed^46^. Recent work suggested that MeCP2 is preferentially recruited to nucleosomes over linear DNA in single molecule assays^49^. This may be mediated through direct histone interaction^46,49–53^. Due to the nature of the assays used, we could not test binding to histones in the absence of DNA. We suggest MeCP2 can recognise features of DNA and histones in isolation that are possibly masked when wrapped into a nucleosome. The preferential engagement of nucleosomes may also be due to the parallel nature of concurrent adjacent entry and exit DNA from a nucleosome, read in combination by the multiple DNA binding motifs found within MeCP2. At the mono-nucleosome level, we find that multiple MeCP2 proteins can interact when sufficient linker DNA is available and that MeCP2 can bridge between linkers to satisfy the full DNA binding footprint (Supplementary Fig. 7B & C). Whether MeCP2 preferentially binds symmetrically between both linkers or asymmetrically on a single nucleosome linker is unclear, and may be context-dependent. Furthermore, how MeCP2 and H1 balance their functions is unclear. Both are present at high levels in the neuronal nucleus and have been reported to compete as well as be co-incident^11,45,49,82,87^. Intriguingly, longer linker lengths did allow the co-occurrence of MeCP2 and H1. Linker lengths are variable between cell lines and different chromatin states^88–90^, therefore, the distribution of nucleosomes may play a role in mediating H1 and MeCP2 engagement. DNA sequence also likely plays a role in relative association^91,92^. We suggest that the exact positioning and abundance of meC and DNA linker-length tunes the balance between MeCP2 and H1, reflecting the changes that occur during neurodevelopment^11^.

MeCP2 recruitment to chromatin is important for mediating its function, but has a complex binding mechanism described as both meC-dependent and independent. In our assays at a nucleosomal level, steep Hill slopes are indicative of multiple sites of cooperative binding, likely through multiple DNA binding modules in MeCP2. We propose a model where DNA-binding motifs in central MeCP2 interact with linker DNA, aiding the binding of the MBD to nucleosome-associated DNA methylation (Fig. 8). Mutation or removal of these DNA-binding regions disrupts MeCP2 binding to nucleosomal meC. However, meC sufficiently distanced from the nucleosome does not require these additional interactions. Previous studies have suggested that regions outside of the MBD stabilise chromatin binding^67^ and the central region binds DNA^35,40^. We show that this DNA-binding region is required for reading of nucleosomal meC and constitutes previously characterised AT hooks 1 and 2, as well as a novel DNA-interacting region (DIR). Primary sequence analysis mapped key residues within the DIR. While these show some overlap with the start of MeCP2’s nuclear localisation signal (NLS), the latter is not required for nuclear accumulation^93^. The motif may also perform DNA-binding functions. Interestingly, the binding appears to be preferential to double-stranded linear DNA. In all binding assays, a total of 200 mM ion concentration, as well as competitor DNA, was used to limit non-specific electrostatic interactions^40^. Under these conditions, the central region of MeCP2 displays a specific preference for linear linker DNA, whereas no nonspecific binding to the general negative charge displayed by a nucleosome core particle was observed at either the acidic patch or bent core nucleosomal DNA. Removal of competitor DNA does allow binding to the nucleosome core particle (Supplementary Fig. 8A), but no distinct complex is resolved on a gel, suggesting instead that aggregation predominates, likely through nonspecific electrostatic interactions.

Beyond the meC interaction, MeCP2 displays a degree of sequence preference. A caveat to note in our assays is that the local DNA sequence surrounding each methylated dinucleotide is only somewhat variable. This is both a consequence of the strong nucleosome positioning sequences used, as well as the recognition sequences of the methyltransferase enzymes required to allow methylation to be placed at specific sites. Nucleosome linker DNA sequence is also variable between the constructs tested, again to allow specific placement of DNA methylation. It is likely that a combination of changes to the DNA sequence affects the flexibility and entry/exit positioning of linker DNA, and a degree of MeCP2 sequence preference alters MeCP2 binding. This is likely seen on unmethylated 37-N_601_-27 nucleosomes with varying linker DNA, resulting in better binding to certain constructs irrespective of methylation state. Indeed, binding to multiple different DNA sequences mimics the ability of MeCP2 to bind broadly across the genome, coincident chiefly with DNA methylation^7,11,22–24^, suggesting that multiple DNA sequences can be tolerated by MeCP2. Future work fully exploring DNA register, DNA methylation and DNA sequence will be invaluable to fully understand MeCP2 recruitment to diverse chromatin substrates found in vivo.

Separation of function mutations of conserved residues in the central region (AT-DIR^mut^) diminishes MeCP2 binding and removes specificity for nucleosomal meC, without affecting the direct MBD-meC interaction. Interestingly, AT-DIR^mut^ also affects MeCP2 binding dynamics in live cells. This highlights the functional importance of the central region of MeCP2. There is a limited number of patient mutations in the central region compared to the clusters in the MBD and NID region (Fig. 5), suggesting that this region may not be as crucial for function. However, the central region does have sections of high conservation. Perhaps, as we have seen with AT-DIR^mut^, multiple mutations of the DNA-binding elements are required to perturb function, an unlikely event in a patient setting. Nevertheless, there are patient mutations in the novel DIR region, including Rett syndrome mutant P225R. Previous work showed that an MeCP2 ‘minigene’, containing only the MBD and NID domains, rescues embryonic lethality of a mouse *Mecp2* knock-out^54^. The results presented here predict that the minigene is insufficient to access the subset of DNA methylation found within the nucleosome core in vivo. In this context, it is interesting that the MeCP2 minigene did not fully rescue the Rett syndrome-like phenotype in mice, unlike a full-length or N-/C-terminal domain deletion construct^13,54^. We suggest that inclusion of the central region could enhance recruitment of truncated MeCP2 to chromatin, prompting a more complete rescue.

## Methods

### Generation of plasmid constructs

Histone and MeCP2 mutations were introduced either using site-directed mutagenesis or direct cloning of synthesised double-strand gBlock fragments containing mutations (Integrated DNA Technologies). Deletions were produced by PCR or Gibson assembly. HisMBP-tagged constructs were cloned using ligation-independent cloning. A summary of constructs generated in this study is listed in Supplementary Table 1.

### Histone purification

Histones were expressed in BL-21 DE3 RIL cells and purified from inclusion bodies essentially as described^94–96^.

Concentrations were determined via absorbance at 280 nm using a Nanodrop One spectrophotometer (Thermo Scientific), followed by SDS-PAGE and colloidal Coomassie staining with comparison to known amounts of control proteins.

For fluorescent octamer labelling, the lyophilised cysteine mutant histone H2B T115C was hydrated to 3 mg.ml^−1^ in resuspension buffer [20 mM Tris pH 7.5, 25 mM NaCl, 0.2 mM TCEP, 7 M Guanidine-HCl] for 30 min at room temperature. Oregon-Green488 maleimide dye (AAT Bioquest) was resuspended in DMSO and added at a 1:1 molar ratio to histone H2B T115C. Samples were incubated for 2 h at 4 °C, spiked with an equivalent volume of dye as initially added and further incubated overnight at 4 °C. Labelling extent was checked by 1D intact weight ESI mass spectrometry (SIRCAMs, School of Chemistry, University of Edinburgh). Labelled histones were either used immediately for octamer assembly or flash frozen in liquid nitrogen and stored at −80 °C.

H1.0 constructs were expressed and purified as previously described for the full-length protein^91^.

### Octamer assembly

Octamers were refolded as previously described^95,97^. Briefly, histones were resuspended in 20 mM Tris pH 7.5, 6 M guanidine, 10 mM DTT and mixed in a mass ratio of 1:1.4:1.6:1.6 H4, H3, H2A, H2B, and diluted to a total concentration of 2 mg.ml^−1^. The histone mixture was dialysed into 15 mM Tris, pH 7.5, 2 M NaCl, 5 mM β-mercaptoethanol, 1 mM EDTA. All octamers were purified using size exclusion chromatography (HiLoad Superdex 200 16/600 or Superdex 200 Increase 10/300 GL Cytiva) in 15 mM Tris pH 7.5, 2 M NaCl, 1 mM EDTA, 5 mM β-mercaptoethanol.

H2A Lys 119 labelling with Alexa647 was performed essentially as previously described^96^ on octamers assembled with H2A K119C, H3.1 C96S C110A, H2B and H4 and desalted in a Zebaspin 7 kDa column (ThermoFisher) to remove β-mercaptoethanol. Seventy micromolar of octamer was incubated with 5 mM TCEP for 10 min at room temperature, 105 µM of AlexaFluor647 C2-maleimide (Invitrogen) was then added and incubated for a further 1 h. Five millimolar β-mercaptoethanol was added to quench, and the reaction was desalted again as above to remove excess dye. Labelling extent was checked by measuring the 650 nm/280 nm absorbance ratio.

### MeCP2 construct purifications

Full-length untagged *H. sapien* MeCP2 (e2 isoform) constructs were expressed in Rosetta (DE3) pLysS *E. coli* cells in LB media for 3 h at 30 °C, induced with 1 mM IPTG. Cells were pelleted at 4000 × *g* for 15 min, snap frozen in liquid nitrogen and stored at −80 °C until use. Cell pellets were thawed and resuspended in lysis buffer [20 mM HEPES pH 8.0, 100 mM NaCl, 0.1% (v/v) reduced Triton X100, 100 µM PMSF, 100 µM benzamidine, 4 mM MgCl_2_, 5 µg.ml^−1^ DNase]. The suspension was nutated at 4 °C for 30 min and sonicated twice (2 s on, 2 s off, for a total of 20 s at 50% amplitude). Bacterial cell debris was pelleted by centrifugation for 45 min at 25,000 × *g*, 4 °C. Clarified lysate was filtered through a 0.4 µm filter, and the buffer was adjusted to 500 mM NaCl and 20 mM imidazole. A native internal stretch of Histidine residues in MeCP2 was utilised for the first step of affinity purification. Lysate was applied to a HiTrap chelating HP column (Cytiva) pre-charged with nickel ions and pre-equilibrated in Nickel A buffer [20 mM HEPES pH 8.0, 500 mM NaCl, 20 mM imidazole, 0.1% (v/v) reduced Triton-X100, 100 µM PMSF, 100 µM benzamidine]. The column was washed with 20 column volumes (CV) of Nickel A buffer and protein bulk eluted with 5 CV of Nickel B buffer [20 mM HEPES pH 8.0, 500 mM NaCl, 500 mM imidazole, 0.1% (v/v) reduced Triton-X100, 100 µM PMSF, 100 µM benzamidine]. Fractions containing the desired protein were pooled and diluted to 250 mM NaCl. Precipitate was removed by centrifugation at 4000 x *g*, 4 °C, for 10 min and filtering through a 0.4 µm filter. The sample was applied to a cation exchange SP HP column (Cytiva) pre-equilibrated in SP A buffer [20 mM HEPES pH 8.0, 280 mM NaCl, 1 mM EDTA, 0.1% (v/v) reduced Triton-X100, 100 µM PMSF, 100 µM benzamidine]. The column was washed with 10 CV of SP A buffer, and protein eluted across a 20 CV gradient from 280 mM to 1 M NaCl. Fractions containing the desired protein were pooled and concentrated in a 10 kDa MWCO centrifugal filter unit. The concentrated sample was applied to a HiLoad Superdex 200 16/60 column (Cytiva) pre-equilibrated in storage buffer [20 mM HEPES pH 7.5, 150 mM NaCl, 5% (v/v) glycerol, 100 µM EDTA, 5 mM β-mercaptoethanol]. Fractions containing the desired protein were pooled, concentrated as before, snap frozen in liquid nitrogen and stored at −80 °C until use.

HisMBP tagged proteins were expressed and purified essentially as described above in Rosetta (DE3) pLysS as described above. Depending on purity, the sample was either concentrated directly after nickel chromatography in a 10 kDa MWCO centrifugal filter unit for the final size-exclusion step or diluted to 150 mM NaCl for an additional ion-exchange step.

His-MBP-DNMT3A_1–427_ and His-MBP-DNMT3A_1–427_ K54A K56A was purified as described^80^.

To remove the His-MBP tag 1 µg of TEV protease was added to every 25 µg of protein, and the sample was incubated at 4 °C overnight with rotation. Precipitate was removed by centrifugation at 4000 × *g*, 4 °C, for 10 min and filtering through a 0.4 µm filter. The sample was then applied to an SP HP column (Cytiva) pre-equilibrated in SP A buffer [20 mM HEPES pH 7.5, 100 mM NaCl, 1 mM EDTA]. The column was washed with 5 CV of SP A buffer, and protein eluted across a 20 CV gradient from 100 mM to 1 M NaCl. Fractions containing the desired protein were pooled. A 3 kDa MWCO centrifugal filter unit was used to concentrate and buffer exchange the sample into storage buffer [20 mM HEPES pH 7.5, 150 mM NaCl, 5% (v/v) glycerol, 100 µM EDTA, 5 mM β-mercaptoethanol]. A sample was analysed by gel filtration on a Superdex 75 10/300 GL column (Cytiva) to check for purity. A BCA assay (Thermo) was used to determine protein concentration for the DIR construct, as it lacked aromatic residues. For all other MeCP2 proteins, Concentrations were determined via absorbance at 280 nm using a Nanodrop One spectrophotometer (Thermo Scientific), followed by iterative SDS-PAGE and colloidal Coomassie staining with comparison to known amounts of control proteins (Supplementary Fig. 2).

### NCP reconstitution

DNA for nucleosome assembly was generated by PCR as previously described^94–96^. Sequences of DNA used are described in Supplementary Table 2, derived from Widom 601 or 603 sequences (addgene plasmid 26656 and 26658, respectively), described and used as previously^80,94,98^ or modified to alter linker length and methyltransferase recognition sites. Fluorescent dyes, biotin tags and asymmetric methyl-cytosine bases were incorporated into HPLC-purified primers used in amplification PCR steps (IDT technologies). Enzymatic methylation was added to DNA at CpG sites using methyltransferase enzymes M.HhaI or M.HpaII (NEB). Reactions were set up with 1 unit of methyltransferase per µg of DNA, 640 µM S-adenosylmethionine (SAM) (NEB) and 1× rCutsmart buffer (NEB). Reactions were incubated for 3 h at 37 °C, spiked with an equivalent volume of SAM as initially added, and further incubated overnight. Methylation state was checked by digestion with methylation-sensitive HhaI or HpaII restriction enzymes (NEB). Samples were ethanol precipitated and resuspended in 10 mM Tris, pH 8.0.

Nucleosomes were reconstituted essentially as described^95,97,98^. Proper assembly of wrapped nucleosomes was analysed by native PAGE and histone composition by SDS-PAGE analysis (Supplementary Fig. 1).

### Hydroxyl radical DNA footprinting

DNA footprinting assays were performed essentially as described^98^. Briefly, 50 ng.µl^−1^ of 5’ 6-FAM labelled 37-N_601_-27 nucleosomes were set up in 10 µl reaction buffer (20 mM Hepes pH 7.5, 200 mM NaCl, 1 mM EDTA, 1 mM DTT). 2.5 µl each of 2 mM Ammonium Iron (II) Sulfate/4 mM EDTA, 0.1 M sodium ascorbate, and 0.12% H_2_O_2_ were simultaneously added to the sample. The reaction was stopped after 4 min by the addition of 100 µl STOP buffer (100 mM Tris pH 7.5, 1% glycerol, 325 mM EDTA, 0.1% SDS, 0.1 mg.ml^−1^ Proteinase K [Thermo]) and incubated for 20 min at 56 °C. Fragmented DNA was purified by ethanol precipitation and resuspended in 10 µl HiDi Formamide. 0.5 µl of GeneScan 500 LIZ size standard (Thermo) was also added as a size marker.

Samples were run on either a 3130xl Genetic or 3730xl DNA Analyzer, operated in accordance with the manufacturer’s instructions using the G5 dye filter set. Peaks were analysed using Thermofisher Connect Microsatellite analysis software. Peak size in base pairs was called by the Global Southern method.

### Electrophoretic mobility shift assays

Nucleosomes were fluorescently labelled either on their DNA component with 5’ 6-carboxyfluorescein (5’ 6-FAM), or by the addition of Oregon-Green488 maleimide dye to H2B at residue 115 (utilising a T115C mutation). Fluorophore addition to DNA or histone was found not to perturb MeCP2 binding. DNA sequences used are shown in Supplementary Table 2. Double-stranded competitor DNA was annealed using 47 bp complementary oligos, incubated at equimolar ratios and heated to 95 °C for 5 min prior to gradual cooling to room temperature.

Competitor DNA: 5’-GGCTGGACACGGAAGCTTAAGCAAGGGAAATCTGGCCGCTCTGCTGG-3’

Competitor DNA was used in all nucleosome binding experiments to promote meC specificity, but was absent from DNA binding experiments, as highlighted in figures and figure legends.

A concentration series of purified protein (e.g. MeCP2) was incubated with 3.75 ng of fluorescent nucleosome in 12 µl of competitor EMSA buffer at a final salt concentration of 200 mM [20 mM HEPES pH 7.5, 75 mM NaCl, 125 mM KCl, 50 µM EDTA, 2.5 mM β-mercaptoethanol, 5% (v/v) glycerol, 333 ng.µl^−1^ BSA, 1.58 µM competitor DNA (see below)]. The reaction was incubated at room temperature for 30 min, after which 3 µl of 5x native loading buffer [40% sucrose, 0.001% bromophenol blue] was added. A 5.2% native polyacrylamide gel was loaded with 10 µl of each sample (2.5 ng of fluorescent nucleosomes loaded) and separated at 100 V, 4 °C. Gels and buffers were either made up of 0.5x TBE or 1× tris glycine. After ~90 min, gels were imaged using a Bio-Rad ChemiDoc MP imaging system, exciting using blue filtered light and recorded using a 532/28 emission filter. ImageLab Touch Software version 3.0.1.14 was used to image. Gels were additionally stained with diamond DNA stain (Promega) and imaged using a Bio-Rad ChemiDoc MP imaging system set to 590/110 emission filter.

Unbound DNA/nucleosome bands were quantified in ImageLab version 6.1 (Bio-Rad) with the ‘lane and bands’ setting, and converted to ‘1 − relative band intensity’ using Eq. 1:

1 $$Y=1-\left[\frac{{D}_{x}-{D}_{\max }}{{D}_{0}-\,{D}_{\max }}\right]$$

where D_x_ is the unbound band intensity at a given protein concentration X, D_0_ is the unbound band intensity at 0 µM of protein, and *D*_max_ is the quantification of an area equal to a band in an empty lane (equivalent to 100% bound). Data was plotted in Prism 9 (GraphPad), with a log10 *x*-axis, and an isotherm fitted using the method ‘specific binding with Hill slope’ described by Eq. 2:

2 $$Y=\frac{{B}_{\max }\times {X}^{h}}{{K}_{d}+\,{X}^{h}}$$

where Bmax is the maximum fraction bound (=1), Kd is the dissociation constant, h is the hill slope, and X is protein concentration. Assumption has been made that non-specific binding has been removed, and the Hill coefficient may be variable. A summary of apparent Kds is reported in Supplementary Table 3. Kd apparent is reported due to the nature of competitor DNA added to the reaction and uncertainty in the absolute value.

### MeCP2-H1 competition assays

The H1 concentration required to form a single clear H1-bound fluorescent nucleosome band was first identified as described for EMSAs assaying protein binding to fluorescent nucleosomes. ‘H1 mix’ is a commercial mix of bovine H1 purified from calf thymus (Sigma-Aldrich, 14–155), predominantly H1.4.

This determined concentration of H1 was incubated with 3.75 ng of fluorescent nucleosomes for 30 min at 4 °C in nucleosome dilution buffer [20 mM HEPES pH 7.5, 250 mM KCl, 666 ng.µl^−1^ BSA, 3.15 µM competitor DNA], in a total volume of 6 µl. A concentration series of purified MeCP2 was then incubated with H1-bound fluorescent nucleosome in competitor EMSA buffer (20 mM HEPES pH 7.5, 75 mM NaCl, 125 mM KCl, 50 µM EDTA, 2.5 mM β-mercaptoethanol, 5% (v/v) glycerol, 333 ng.µl^−1^ BSA, 1.58 µM competitor DNA). A final reaction volume of 12 µl was incubated at room temperature for 30 min, after which 3 µl of 5× native loading buffer was added. 10 µl of the sample (2.5 ng of fluorescent nucleosome) was loaded onto a 6% native polyacrylamide gel and separated at 100 V, 4 °C. Gels and buffer were made up of 1× TG. After 120 min the gel was imaged as before.

### MeCP2-H1-nucleosome super-shift assay

Two hundred nanomolar of H1.0 was incubated with 3.75 ng of fluorescent 37-N_601_-27 nucleosomes for 30 min at 4 °C in nucleosome dilution buffer [20 mM HEPES pH 7.5, 250 mM KCl, 666 ng.µl^−1^ BSA, 3.15 µM competitor DNA)], in a total volume of 6 µl. 250 nM of purified MeCP2 was then incubated with H1 bound fluorescent nucleosome in competitor EMSA buffer (20 mM HEPES pH 7.5, 75 mM NaCl, 125 mM KCl, 50 µM EDTA, 2.5 mM β-mercaptoethanol, 5% (v/v) glycerol, 333 ng.µl^−1^ BSA, 1.58 µM competitor DNA), in a total volume of 12 µl for 30 min at room temperature.

0.5 µl of Polyclonal H1.0 antibody (Abcam: *ab154111*) (lot: GR3413078-6) was then added (1:25 dilution), and the 12.5 µl reaction was incubated for a further 30 min. Three microliters of 5× native loading buffer was added, and 10 µl of the sample (2.4 ng of fluorescent nucleosome) was loaded onto a 5.2% native polyacrylamide gel and separated at 100 V, 4 °C. Gels and buffer were made up of 1× TG. After 120 min the gel was imaged as before.

### Bioinformatic analysis

Disease-causing missense mutations from ClinVar were plotted along the length of the protein using PlotProtein^99,100^. Predicted missense severity scores were generated using Alphamissense^74^. Scores for each residue across all possible amino acids were then summed.

ClinVar mutations of interest within the MeCP2 central region were: R190H (VCV002664668.2), P217L (VCV000236302.4), P225R (VCV000143653.48), R255G (VCV000978959.2) and K266E (VCV000548706.1).

Species listed for sequence comparison are *Mus musculus* (House mouse), *Xenopus laevis* (African clawed frog), *Notamacropus eugenii* (Tammar wallaby), *Eudyptula minor* (Little penguin), *Danio rerio* (Zebrafish) and *Callorhinchus milii* (Ghost shark), with sequences retrieved from UniProt. The motif was also identified in *H. sapiens* DNMT3A and LEDGF using ScanProsite^101^ and BLASTP 2.12.0 + ^102^ against the UniprotKB_RefProtSwissProt database. The motif in LEDGF was found by a literature search^79^.

### MeCP2 PFV-GAG competition

GST-tagged PFV-GAG protein was purified as previously described^98^. Two micromolar of MeCP2, or 500 nM of HisMBP-MeCP2_162–309_, was incubated with 3.75 ng of fluorescent nucleosomes for 30 min at 4 °C in nucleosome buffer in a total volume of 6 µl. A concentration series of GST-PFV GAG was then incubated with MeCP2-bound fluorescent nucleosomes in competitor EMSA buffer. Samples were incubated and loaded on gels as before. Gels were 5.2% native polyacrylamide.

### Microscale thermophoresis

Microscale thermophoresis (MST) measurements were performed on a Monolith NT.115 Pico instrument (NanoTemper Technologies) using standard or premium capillaries. Reconstituted H2A^K119C^ alexa647 labelled nucleosomes (at 10 nM each) were mixed 1:1 with a dilution series of MeCP2 (WT or AT-DIR^mut^) in a final buffer containing 200 mM salt [20 mM HEPES pH 7.5, 75 mM NaCl, 125 mM KCl, 0.05 mM EDTA, 5% glycerol, 0.287 mg.ml^-1^ BSA, 0.02% NP-40, 2.5 mM β-mercaptoethanol, 0.3 µM competitor DNA] and incubated at room temperature for 30 min. All MST measurements were carried out at 22 °C using 5% Pico-Red excitation power and ‘Medium’ MST power, with 30 s laser ON and 5 s laser OFF time. The cold phase was defined as the average signal 1 to 3 s before excitation, whilst the hot phase was defined as 1.9 to 3.9 s after excitation (Supplementary Fig. 12). Data were collected using MO. Control version 1.6 software, and exported from MO.affinity analysis version 2.3 software. Fnorm was calculated using Eq. 3:

3 $${{\rm{Fnorm}}}=\frac{{{\rm{Hot\; phase}}}\times 1000}{{{\rm{Cold\; phase}}}}$$

MST data were plotted in Prism version 10.6.0 (GraphPad), with a log10 x-axis, and an isotherm fitted using ‘EC50 shift, X is concentration’. ∆Fnorm was calculated by subtracting the bottom estimate from each Fnorm value. ∆Fnorm data were fitted with an isotherm using the method ‘specific binding with Hill slope’, as performed for EMSA experiments.

### Mass photometry

Mass photometry data were collected using a TwoMP mass photometer (Refeyn), calibrated with β-amylase (56, 112, 224 kDa) and thyroglobulin (670, 1340 kDa). Movies were acquired for 2845 frames using AcquireMP software version 2024 R2.1.

Nucleosome:MeCP2 complexes were mixed to a final nucleosome concentration of 50 ng.µl^−1^ in MP buffer containing 200 mM salt [20 mM Na_2_HPO_4_/NaH_2_PO_4_ pH 7.5, 2 mM HEPES pH 7.5, 75 mM NaCl, 125 mM KCl, 0.5 mM EDTA, 2 mM DTT, 0.1% glycerol], and incubated on ice for 2 h. Final molar ratios of nucleosome:MeCP2 were 1:2 for 37-N_601_-27 nucleosomes, and 1:4 for 15-N_601_-15 nucleosomes. Complexes, and nucleosomes alone, were then diluted 100-fold in dilution buffer [20 mM Na_2_HPO_4_/NaH_2_PO_4_ pH 7.5, 75 mM NaCl, 125 mM KCl, 0.5 mM EDTA, 2 mM DTT], to a final nucleosome concentration of either 3.9 nM (37-N_601_-27 nucleosomes) or 4.6 nM (15-N_601_-15 nucleosomes).

Raw data were exported from DiscoverMP software version 2024 R2.1, after determining mass using calibrated standards, and plotted as a frequency distribution using Prism version 10.6.0 (GraphPad) and a bin size of 5 kDa. Data was fitted with either a ‘Sum of two Gaussians’ (for 15-N_601_-15 nucleosomes) or ‘Sum of three Gaussians’ (for 37-N_601_-27 nucleosomes).

### Size exclusion chromatography coupled to multi-angle light scattering (SEC-MALS)

Size-exclusion chromatography (AKTA PURE25^TM^; Cytiva) coupled with UV, static light scattering and refractive index (RI) detection (Viscotec SEC-MALS 20 and Viscotek RI Detector VE3580; Malvern Instruments) was used to determine the absolute molecular mass of untagged MeCP2 DIR (MeCP2_205–257_) in solution. A 100 µL injection of 2.11 mg.ml^−1^ (375 µM) untagged MeCP2_205–257_ was run on a calibrated Superdex-75 Increase 10/300 GL (Cytiva) size exclusion column pre-equilibrated in 20 mM HEPES, pH 7.5, 150 mM NaCl, 0.1 mM EDTA, 2.5% (v/v) glycerol at 22 °C with a flow rate of 1.0 ml.min^−1^. Light scattering, RI and A280 (protein elution from the chromatography system was monitored at 230 nm due to the protein construct having no aromatics) were analysed by a homo-polymer model (OMNISEC software, v5.1; Malvern Instruments) using the following parameters for MeCP2 DIR (MeCP2_205–257_): d*A*/d*c* = 0.01 AU.mL^−1^.mg^−1^, d*n*/d*c* = 0.185 ml.g^−1^ and buffer RI value of 1.3362. Peak fractions were run on a 17% SDS-PAGE gel for analysis.

### Surface plasmon resonance (SPR)

Nucleosomes wrapped with 37-N_601_-27 biotinylated DNA were immobilised on streptavidin sensor surfaces at 20 nM, at 25 ° C at a flow rate of 5 µl.min^−1^ on a BIAcore T200 instrument (Cytiva) equilibrated in 200 mM salt SPR Buffer [20 mM HEPES, pH 7.5; 200 mM KCl; 1 mM EDTA; 0.5 mM DTT; 0.02% NP-40; 0.33 mg.ml^−1^ BSA], Flow cell 1 (blocked with 20 nM D-biotin in SPR Buffer) was used as the bulk reference surface, while 3× meCpG, −1 meCpG or unmethylated nucleosomes were separately immobilised on flow cells 2, 3 and 4, respectively, to between 1540 and 1580 RU. A 2-fold dilution series (12.5−200 nM) of WT or AT-DIR^mut^ MeCP2 protein, supplemented with 50 ng.µl^−1^ of competitor DNA, was injected at 30 µl.min^−1^, in a single-cycle kinetic experiment, each with a 60 s contact time, a 60 s dissociation phase, and a final 600 s dissociation. A 30 s injection, at 30 µl.min^−1^ of 20 mM HEPES (pH 7.4); 1.5 M KCl; 1 mM EDTA, 0.5 mM DTT, 0.02% P20; 0.33 mg.ml^-1^ BSA was used to regenerate the surface between cycles. Data was collected using Biacore T200 version 3.2 software and analysed in Prism 10.6.0 (GraphPad).

### Crosslinking mass spectrometry

Complexes of MeCP2 bound to methylated 175 bp nucleosomes (2.5:1 molar ratio) were crosslinked using a concentration range of photo-reactive sulfo-SDA (sulfosuccinimidyl 4,4’-azipentanoate) (Thermo Fisher) in a w/w ratio of 1:0.25-1.5 (nucleosome: sulfo-SDA) in crosslink buffer [20 mM HEPES pH7.5, 250 mM NaCl, 1 mM EDTA, 1 mM DTT]. A total reaction volume of 30 µl, containing 7.5 µg of nucleosome, was incubated for 2 h on ice prior to UV irradiation at 365 nm in a CL-1000L UV cross-linker (Spectrum) for 20 min. The reaction was immediately quenched with 40 mM ammonium bicarbonate. The crosslinked sample was separated on an 3–8% gradient nuPAGE gel (Invitrogen) and bands running at a higher molecular weight than MeCP2 were excised. Protein gel bands were reduced with 10 mM TCEP for 30 min at 37 °C, alkylated with 55 mM iodoacetamide for 20 min at room temperature and digested using 13 ng.μl^−1^ trypsin (Promega) overnight at 37 °C. Digested peptides were desalted using C18-StageTips^103^ for LC-MS/MS analysis.

LC-MS/MS analysis was performed using Orbitrap Fusion Lumos (Thermo Fisher Scientific) with a ‘high/high’ acquisition strategy. The peptide separation was carried out on an EASY-Spray column (50 cm × 75 μm i.d., PepMap C18, 2 μm particles, 100 Å pore size, Thermo Fisher Scientific). Mobile phase A consisted of water and 0.1% v/v formic acid. Mobile phase B consisted of 80% v/v acetonitrile and 0.1% v/v formic acid. Peptides were loaded at a flow rate of 0.3 μl.min^-1^ and eluted at 0.2 μl.min^-1^ using a linear gradient going from 2% mobile phase B to 40% mobile phase B over 139 min (each sample has been running three time with different gradient), followed by a linear increase from 40% to 95% mobile phase B in 11 min (160 min total run time). The eluted peptides were directly introduced into the mass spectrometer. MS data were acquired in the data-dependent mode with 3 s acquisition cycle. Precursor spectrum was recorded in the Orbitrap with a resolution of 120,000. The ions with a precursor charge state between 3+ and 8+ were isolated with a window size of 1.6 m/z and fragmented using high-energy collision dissociation (HCD) with collision energy 30. The fragmentation spectra were recorded in the Orbitrap with a resolution of 30,000. Dynamic exclusion was enabled with a single repeat count and 60 s exclusion duration.

Peak lists were generated with ProteoWizard (version 3.0.24283)^104^, and cross-linked peptides were matched to spectra using Xi software (version 1.8.4.1)^105^; Xi search) within-search assignment of monoisotopic peaks^106^. MS1 accuracy was set to 3 ppm and MS2 accuracy to 10 ppm. Trypsin was the protease of choice, allowing four missed cleavages, and SDA was chosen from the crosslinker list. Carbamidomethylation of cysteine was chosen as a fixed modification, and oxidation of methionine was chosen as a variable modification. TR False discovery rate was computed using XiFDR and results reported at 1% residue level false discovery rate^107^. Our in-house protein database was used for the searches, containing MeCP2, and histones H2A, H2B, H3.1 and H4 (all *Homo sapiens*).

### Cell culture and transfection

NIH-3T3 mouse fibroblasts (ECACC, 93061524) (*Mus. Musculus*, male, ATCC CRL-1658) were cultured in Dulbecco’s Modified Eagle Medium (DMEM; Gibco ref. 41966029) supplemented with 10% foetal bovine serum and were grown at 37 °C with 5% carbon dioxide. For imaging, 1.5 × 10^5^ cells were plated and cultured directly on polymer coverslips (iBidi cat. 81156) with gelatine coating and the appropriate culture conditions. NIH-3T3 cells were transfected with 2 µg of wild-type eGFP-MeCP2 or mutant versions (∆163-271, AT-DIR^mut^, R133C) using Lipofectamine 2000 (Thermo Fisher Scientific cat. 11668019), following the manufacturer’s protocol.

Cells were tested for Mycoplasma contamination (Lonza cat. LT07-218) and cell identity verified by Sanger sequencing of mitochondrial Cytochrome b using universal primers (Fwd: CGAAGCTTGATATGAAAAACCATCGTTG, Rev: AAACTGCAGCCCCTCAGAATGAT ATTTGTCCTCA).

### Live cell imaging

Live cells were imaged 24 h after transfection. NucBlue Live Cell (ThermoFisher cat. R37605) stain was added prior to imaging, as directed by the manufacturer, using a Zeiss LSM 880 confocal microscope with an Airyscan module and environmental chamber at 37 °C with 5% carbon dioxide. Images were acquired using Zeiss ZEN software (black edition) and processed using Fiji software.

### Fluorescence recovery after photo-bleaching (FRAP)

Live cells were analysed the day after transfection, and FRAP was performed using a Zeiss LSM 880 confocal microscope, equipped with an Airyscan module and environmental chamber at 37 °C with 5% carbon dioxide. For each cell, the eGFP-MeCP2 signal was imaged every 1 s for 400 s with five images recorded before bleaching at a selected MeCP2-enriched spot (FRAP spot) with 100% laser power.

FRAP analysis of three independent transfection experiments was performed using a custom macro with Fiji software (10.5281/zenodo.2654601). Mean pre-bleach fluorescence was estimated per cell to control for transfection efficiency (Supplementary Fig. 14). Fluorescence was measured at the bleached MeCP2 spot (FRAP spot), as well as a non-bleached MeCP2 spot (control spot) to account for photobleaching during the experiment. Additionally, the fluorescence outside of transfected cells was measured as background. The first time point (T0) was defined as the first post-bleach image. For each time point, the FRAP fluorescence signal was normalised to fluorescence values before photobleaching, as described in Eq. 4:

4 $$	{{\rm{Normalised}}}\; {{\rm{FRAP}}}=\frac{({{\rm{FRAP}}}\; {{\rm{spot}}})t-({{\rm{background}}})t}{({{\rm{control}}}\; {{\rm{spot}}})t-({{\rm{background}}})t} \\ 	\times \frac{{{\rm{mean}}}[({{\rm{control}}}\; {{\rm{spot}}}){{\rm{prebleach}}}-({{\rm{background}}}){{\rm{prebleach}}}]}{{{\rm{mean}}}[({{\rm{FRAP}}}\; {{\rm{spot}}}){{\rm{prebleach}}}-({{\rm{background}}}){{\rm{prebleach}}}]}$$

A ‘Two phase association’ model (nonlinear regression) was used to fit experimental data using the software GraphPad Prism 10. The plateau (fluorescence at the last time point of the FRAP experiment), corresponding to the mobile fraction, was interpolated from the fitted curve. The T1/2 (time to recover 50% of fluorescence at plateau) was interpolated from the fitted curve. Data was plotted using GraphPad Prism 10.

### Reporting summary

Further information on research design is available in the Nature Portfolio Reporting Summary linked to this article.

## Supplementary information

Supplementary Information

Reporting Summary

Transparent Peer Review File

## Source data

Source Data File 1

Source Data File 2

Source Data File 3

Source Data File 4

Source Data File 5

## Data Availability

Source Data for Figs. 1–7 and associated Supplementary Figs. 1–16 are provided with this paper. Reagents that support the findings of this study are available from the corresponding author upon reasonable request. The authors declare that all other data supporting the findings of this study are available within the paper and its supplementary and source information files. Crosslinking mass spectrometry data has been uploaded to the PRIDE database under identifier PXD064826. Protein structures used for modelling are available on the PDB, accession codes 3c2i and 3LZ0. Source data are provided with this paper.

## Code availability

The script for FRAP analysis using a custom macro with Fiji software is available on Zenodo (10.5281/zenodo.2654601).
