# Supplementary material for: MeCP2 requires interactions with nucleosome linker DNA to read chromatin DNA methylation
Source: Nat Commun. 2026 Apr 17;17:5374. doi: 10.1038/s41467-026-71741-0 (PMC13276077; doi:10.1038/s41467-026-71741-0)
Supplement: Supplementary file 1 — Supplementary Information [file 41467_2026_71741_MOESM1_ESM.pdf]

Supplementary Information

MeCP2 requires interactions with nucleosome linker DNA to read chromatin DNA methylation

James A. Watson[1], Beatrice K. Alexander-Howden[1], Theo S. Hall[1], Martin A. Wear[2], Finlay McGhie[1], Gillian Clifford[1], Hannah Wapenaar[1], Juan Zou[1], Adrian Bird[1*], Marcus D. Wilson[1,2*]

[1] Centre for Cell Biology, University of Edinburgh, Michael Swann Building, Kings Buildings, Mayfield Road, Edinburgh, EH9 3JR, UK

[2] Institute of Quantitative Biology, Biochemistry and Biotechnology, Kings Buildings, University of Edinburgh, Edinburgh EH9 3JR, UK

[*]Correspondence should be addressed to a.bird@ed.ac.uk & marcus.wilson@ed.ac.uk

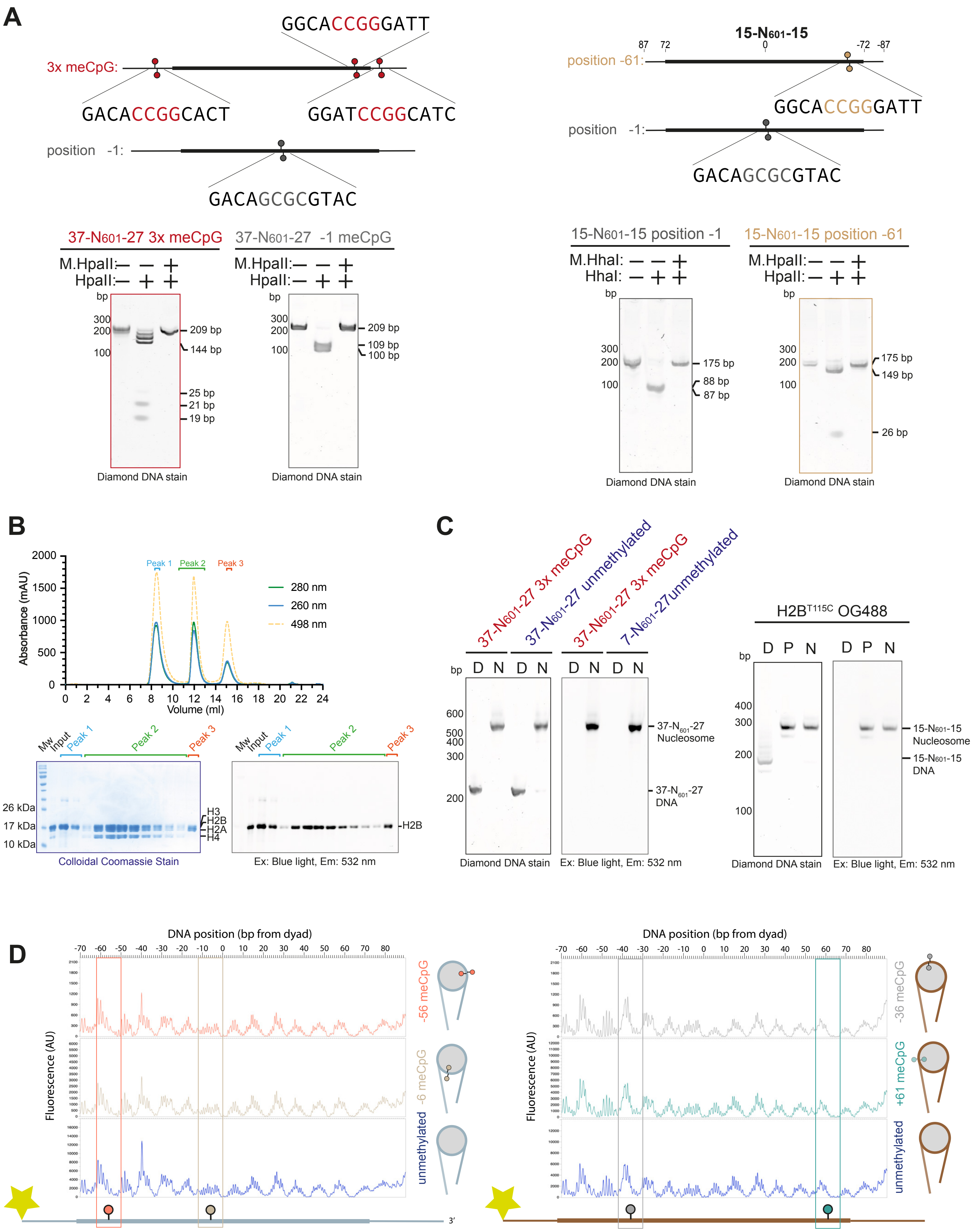

A
GGCACCGGGATT
3x meCpG:
GACACCGGCACT
GGATCCGGCATC
position -1:
GACAGCGCGTAC
15-N601-15
position -61:
GGCACCGGGATT
position -1:
GACAGCGCGTAC
37-N601-27 3x meCpG
37-N601-27 -1 meCpG
15-N601-15 position -1
15-N601-15 position -61
M.HpaII:
HpaII:
M.HhaI:
HhaI:
209 bp
144 bp
25 bp
21 bp
19 bp
109 bp
100 bp
175 bp
88 bp
87 bp
149 bp
26 bp
Diamond DNA stain
B
Absorbance (mAU)
Volume (ml)
Peak 1
Peak 2
Peak 3
280 nm
260 nm
498 nm
Mw
Input
26 kDa
17 kDa
10 kDa
H3
H2B
H2A
H4
Colloidal Coomassie Stain
Ex: Blue light, Em: 532 nm
C
37-N601-27 3x meCpG
37-N601-27 unmethylated
37-N601-27 3x meCpG
7-N601-27unmethylated
37-N601-27 Nucleosome
37-N601-27 DNA
H2BT115C OG488
15-N601-15 Nucleosome
15-N601-15 DNA
Diamond DNA stain
Ex: Blue light, Em: 532 nm
D
DNA position (bp from dyad)
Fluorescence (AU)
-56 meCpG
-6 meCpG
unmethylated
-36 meCpG
+61 meCpG
unmethylated
3'

Supplementary Figure 1: meCpG placement on Widom601 DNA.

A. Restriction digest reaction of methylated and unmethylated 15-N_{601}-15 and 37-N_{601}-27 DNA by M.HhaI or M.HpaII on native-PAGE. In both constructs M.HhaI positions meCpG at -1 bp from the dyad, M.HpaII positions meCpG at -61 bp from the dyad. Additionally M.HpaII positions meCpG at -80 and 83 bp from the dyad in 37-N_{601}-27. A lack of digestion upon addition of HhaI or HpaII restriction enzyme is indicative of complete methylation. Final expected DNA sizes upon complete digestion are marked. The DNA sequence +/- 5 bp from each meCpG site is shown.

B. Size exclusion chromatography trace of assembled OregonGreen488 (OG488) labelled $H2B^{T115C}$ octamers produced three distinct peaks. OG488 dye was followed by absorbance at 498 nm (yellow, dashed), protein at 280 nm (green, solid) and DNA at 260 nm (blue, solid). Peak fractions were run on a 17% SDS-PAGE gel, imaged for fluorescence, and stained for protein. Peak 1 primarily contained excess H2B, peak 2 the histone octamer, and peak 3 excess H2A H2B dimer.

C. Representative native polyacrylamide gel of $H2B^{T115C}$ OG488 labelled octamers wrapped with either 37-N_{601}-27 or 15-N_{601}-15 DNA. The gel was imaged for fluorescence before being stained for DNA. A band shift is indicative of nucleosome formation (N). 15-N_{601}-15 nucleosomes were purified from excess DNA (P) by precipitation with PEG6000. Unwrapped DNA (D) is included as a control.

D. Representative Hydroxyl radical footprinting of 5' 6-FAM labelled 37-$N_{601(mod)}$-27 and 37-$N_{601(mod2)}$-27 nucleosomes, either unmethylated (blue), or methylated with meCpG at -56 bp (orange), -6 bp (light brown), -36 bp (light grey), and 61 bp (teal). A 10 bp periodicity of protected bases is indicative of histone-DNA interactions. Location of methylated DNA on the analysed strand and footprinting traces is indicated below. +/- 5 bp from the meCpG site is highlighted by boxes.

Source data are provided as Source Data Files 3-4.

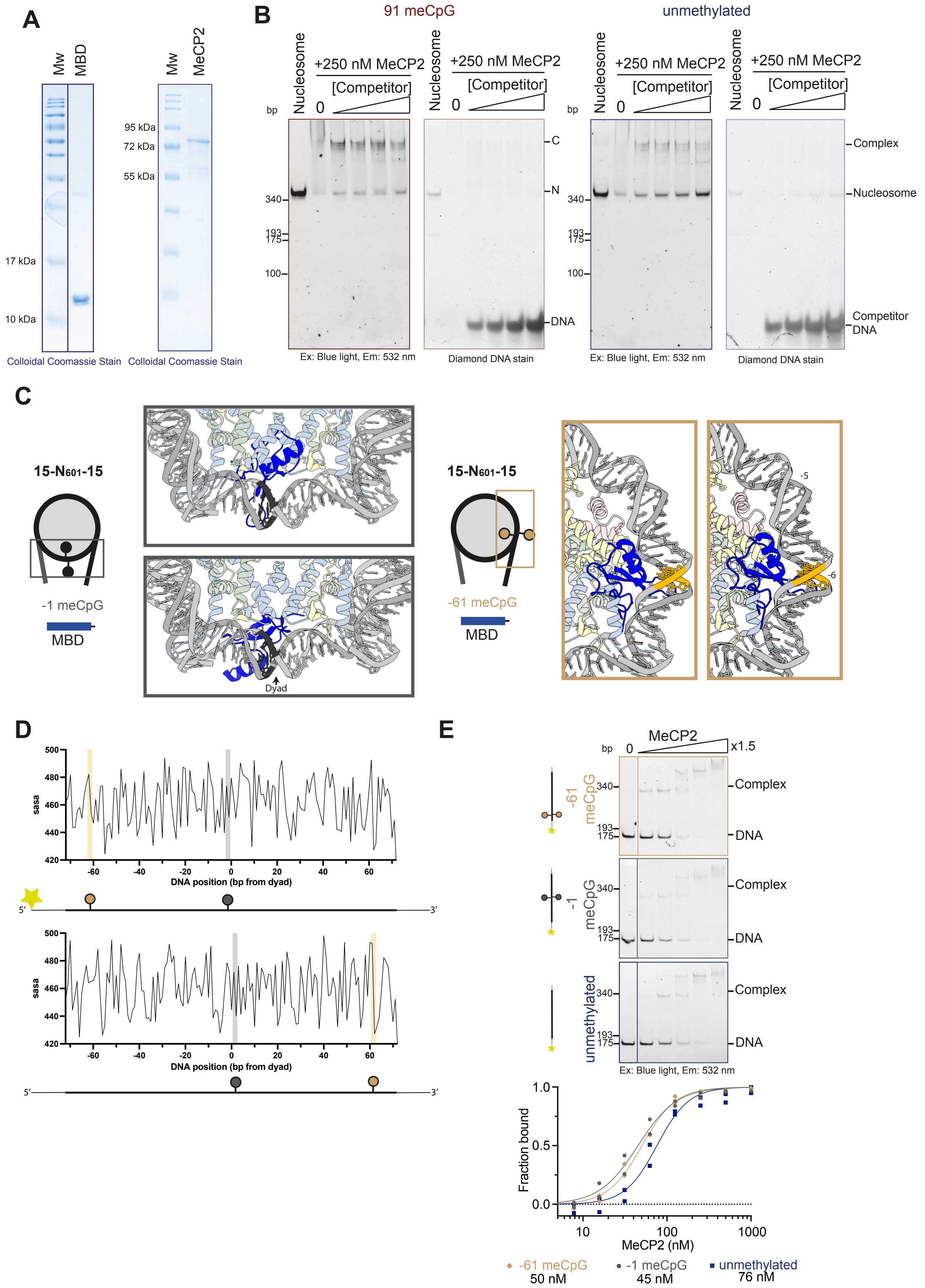
A
Mw
MBD
MeCP2
95 kDa
72 kDa
55 kDa
17 kDa
10 kDa
Colloidal Coomassie Stain
Colloidal Coomassie Stain
B
91 meCpG
unmethylated
Nucleosome
+250 nM MeCP2
[Competitor]
0
bp
340
193
175
100
C
N
DNA
Complex
Nucleosome
Competitor DNA
Ex: Blue light, Em: 532 nm
Diamond DNA stain
C
15-N601-15
-1 meCpG
MBD
Dyad
-61 meCpG
-5
-6
D
sasa
DNA position (bp from dyad)
5′
3′
E
MeCP2
x1.5
-61 meCpG
-1 meCpG
unmethylated
Complex
DNA
Ex: Blue light, Em: 532 nm
Fraction bound
MeCP2 (nM)
-61 meCpG 50 nM
-1 meCpG 45 nM
unmethylated 76 nM

Supplementary Figure 2: meCpG positioning on wrapped nucleosomes.

A. SDS-PAGE gels loaded with 1 µg of each purified construct. MBD (left) and full-length MeCP2(right). The 52 kDa MeCP2 protein runs at around 72 kDa.

B. Representative EMSA native gel (2 repeats) showing the effect of a two-fold dilution series of competitor DNA (0.4, 0.8, 1.6, 3.2 µM) on the binding of full-length MeCP2 (250 nM) to 16-N_{603}-30 nucleosomes. Nucleosomes were either methylated with meCpG at position 1 (red), or unmethylated (blue).

C. Location of meCpG dinucleotides -1 bp (grey) and -61 bp (brown) from the dyad, shown both as cartoons and on the crystal structure of a Widom 601 nucleosome (PDB: 3LZ0[1]). The crystal structure of MeCP2 MBD (PDB: 3C2I[2], blue) was modelled into the major grooves by alignment of the meCpG site, producing the two possible orientations shown for each site. In all cases the MBD clashes with the histone octamer.

D. Solvent accessibility surface (SASA) plot of a Widom 601 nucleosome (PDB: 3LZ0[1]). SASA values were generated for each DNA base with a probe radius of 1.4 Å (approximation of a water molecule). The calculation was performed for both strands of DNA, indicated by cartoons below. Again sites of methylated DNA are shown, and highlighted on the plots by coloured boxes.

E. Representative EMSA native-PAGE (2 repeats) showing a 2-fold dilution series of MeCP2 with limiting amounts (2.5 ng) of 5' 6-FAM labelled 15-N_{601}-15 DNA. Concentrations 31.3-500 nM on the gel are shown for clarity. DNA was methylated with a single meCpG either -61 bp (brown) or -1 bp (grey) from the dyad, or unmethylated (blue). Free DNA and complex bands are indicated, size markers in bp are shown. Quantification of the free DNA bands at each concentration, of the full concentration series (1.95-4000 nM), was fitted with a binding isotherm and an apparent dissociation constant ($K_{D\ app}$) calculated. Individual datapoints at each concentration represent a repeat. Full calculated $K_{D\ app}$ and hill slope statistics are summarised in Supplementary Table 3.

Source data are provided as Source Data Files 3-4.

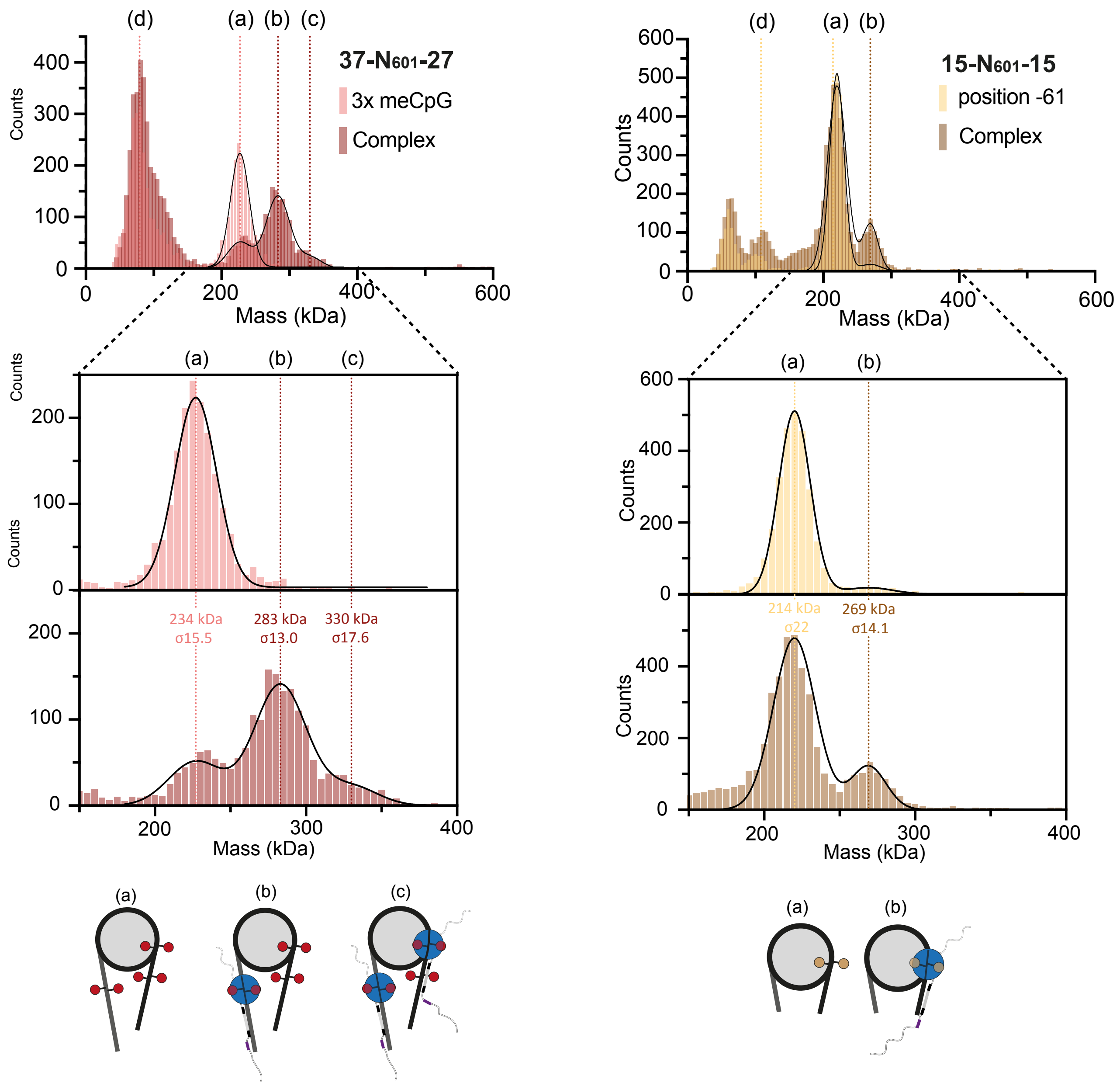

Supplementary Figure 3: Further analysis of MeCP2-nucleosome complexes.

Mass photometry (MP) analysis for the complexes formed by MeCP2 on 37-N_{601}-27 nucleosomes (left) in a 2:1 molar ratio, or on 15-N_{601}-15 nucleosomes (right) in a 4:1 molar ratio. MP of both nucleosomes alone is also shown for comparison. Based on expected mass, peak (a) corresponds to unbound nucleosome, whilst (b)-(c) are 1:1 and 1:2 nucleosome:MeCP2 complexes. Cartoons are shown below to describe the potential contents of each peak. However, due to the presence of multiple binding sites the actual positioning of MeCP2 on nucleosomes within each peak is likely more variable than depicted. Due to the limiting amounts of MeCP2 used, additional binding events were not observed. In all examples peaks were also observed measuring at around 100 kDa (d), which likely corresponds to unwrapped free DNA. Peaks were fitted to a sum of Gaussians. The mean of the fitted peaks is shown as the estimated molecular weight, whilst σ represents standard deviation.
Source data are provided as Source Data Files 3.

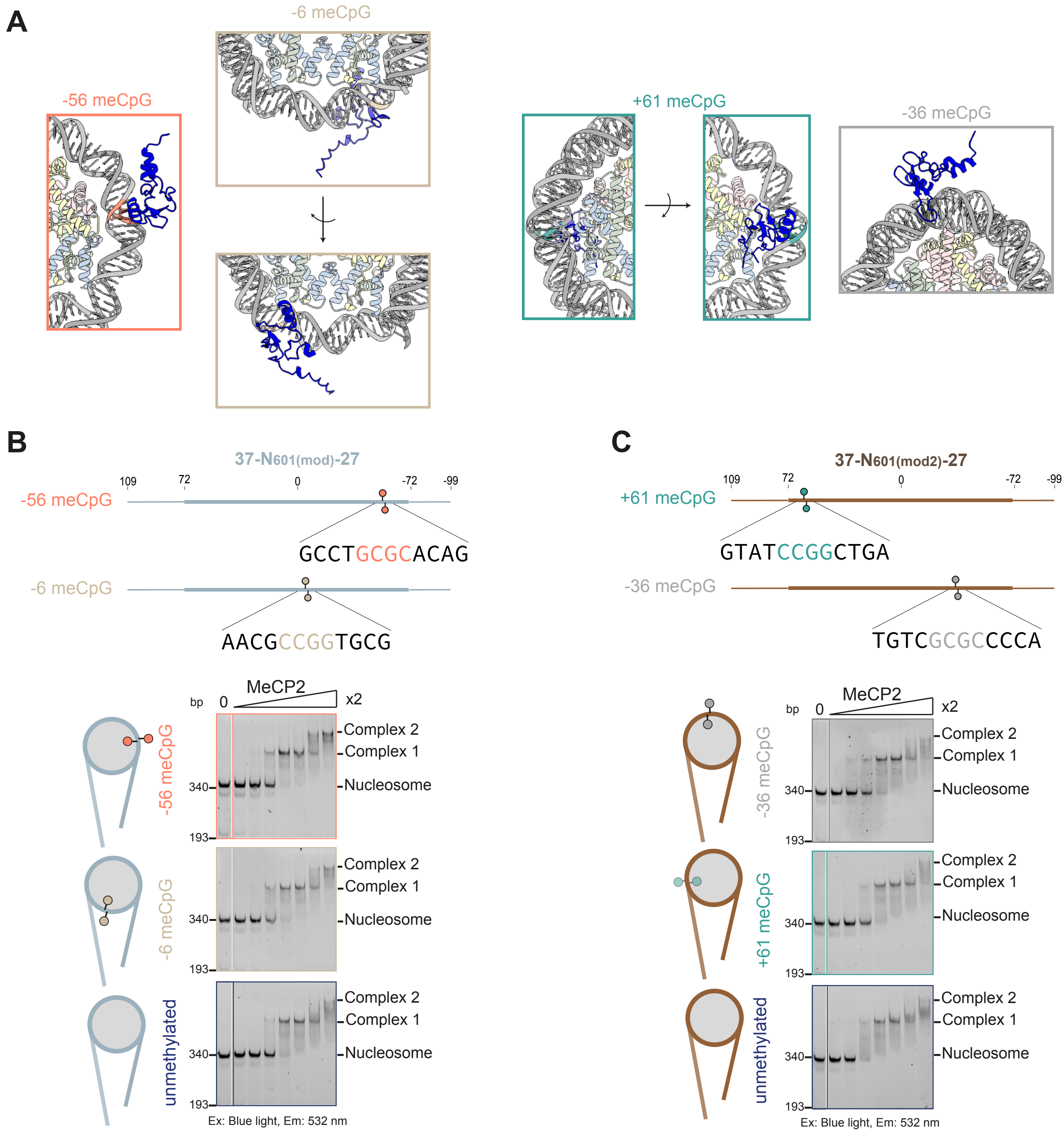
A
-6 meCpG
-56 meCpG
+61 meCpG
-36 meCpG
B
37-N601(mod)-27
109
72
0
-72
-99
-56 meCpG
GCCTGCGCACAG
-6 meCpG
AACGCCGGTGCG
C
37-N601(mod2)-27
+61 meCpG
GTATCCGGCTGA
-36 meCpG
TGTCGCGCCCCA
MeCP2
bp
0
x2
Complex 2
Complex 1
Nucleosome
340
193
-56 meCpG
-6 meCpG
unmethylated
-36 meCpG
+61 meCpG
Ex: Blue light, Em: 532 nm

Supplementary Figure 4: MeCP2 binds alternate meCG sites in core nucleosomal DNA.

A. Location of meCpG dinucleotides -56 bp (orange), -6 bp (light brown), -36 bp (light grey), and 61 bp (teal) from the dyad on the crystal structure of a Widom 601 nucleosome (PDB: 3LZ0[1]). The crystal structure of MeCP2 MBD (PDB: 3C2I[2]) was modelled into the major grooves by alignment of the meCpG site. Only position 61 produces clashes between the MBD and the histone octamer.

B. Representative EMSA native-PAGE (3 repeats) showing a 2-fold dilution series of MeCP2 with limiting amounts (2.5 ng) of 5' 6-FAM labelled 37-$N_{601(mod)}$-27 nucleosomes. Concentrations 7.8-500 nM on the gel are shown for clarity. The core Widom 601 sequence was altered to accommodate specific single meCpG positioning. Nucleosomes were methylated with meCpG either at position -6 (light brown) or -56 (orange), or unmethylated (blue). Quantification is shown in Figure 2A.

C. Representative EMSA native-PAGE (3 repeats) showing a 2-fold dilution series of MeCP2 with limiting amounts (2.5 ng) of 5' 6-FAM labelled 37-$N_{601(mod2)}$-27 nucleosomes. Concentrations 7.8-500 nM on the gel are shown for clarity. The core Widom 601 sequence was altered to accommodate specific single meCpG positioning. Nucleosomes were methylated with meCpG either at position -36 (light grey) or +61 (teal), or unmethylated (blue). Quantification is shown in Figure 2B.

Source data are provided as Source Data Files 4.

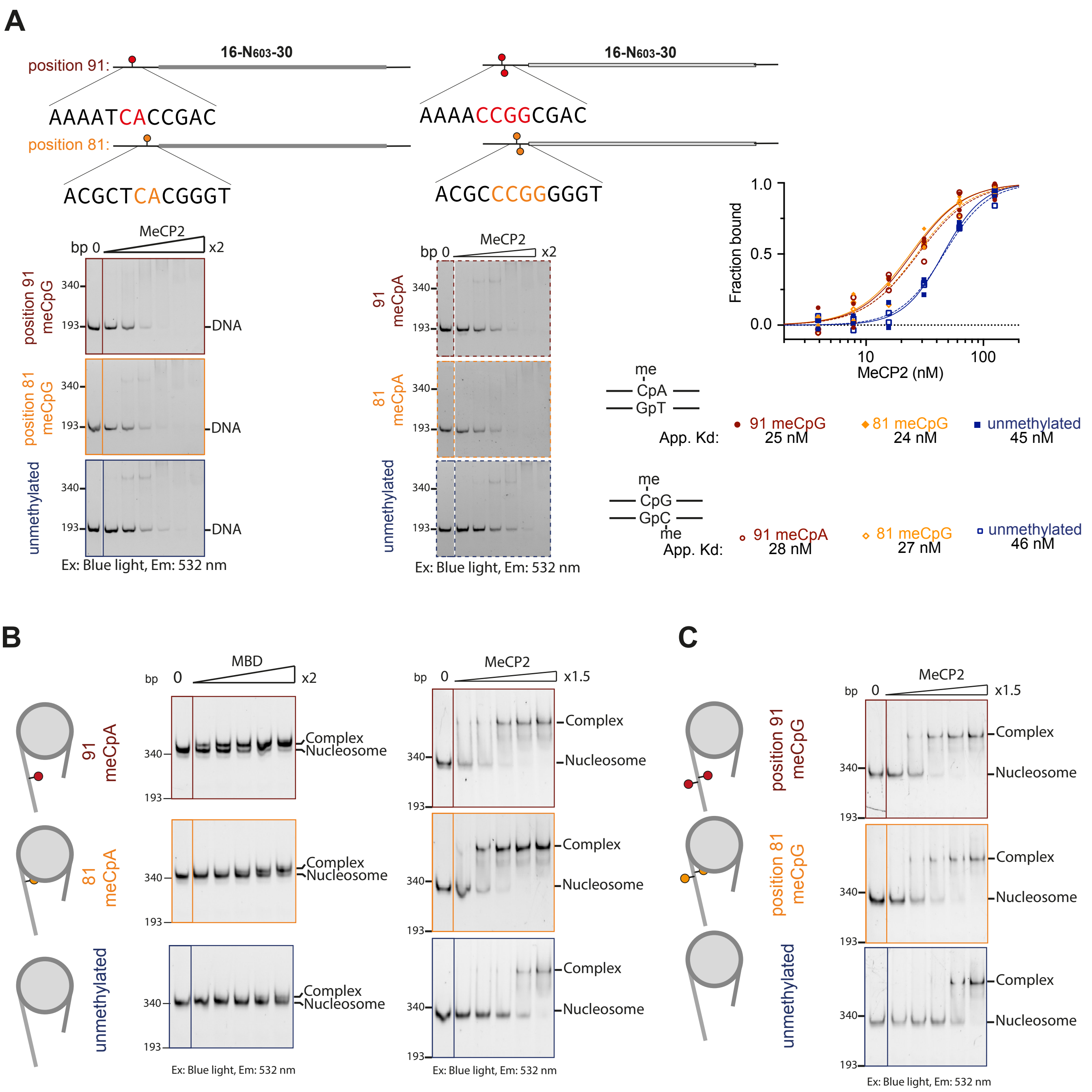
A
position 91:
16-N603-30
AAAATCACCGAC
AAAACCGGCGAC
position 81:
ACGCTCACGGGT
ACGCCCGGGGGT
MeCP2
bp 0
x2
position 91 meCpG
position 81 meCpG
unmethylated
91 meCpA
81 meCpA
340
193
DNA
Ex: Blue light, Em: 532 nm
Fraction bound
1.0
0.5
0.0
10
100
MeCP2 (nM)
me
CpA
GpT
App. Kd:
91 meCpG
25 nM
81 meCpG
24 nM
unmethylated
45 nM
CpG
GpC
91 meCpA
28 nM
81 meCpG
27 nM
unmethylated
46 nM
B
MBD
x1.5
Complex
Nucleosome
C
position 91 meCpG
position 81 meCpG

Supplementary Figure 5: MeCP2 binds meCG and meCA sites on nucleosomal linker DNA.

A. Representative EMSA native-PAGE (2 repeats) showing a 2-fold dilution series of MeCP2 with limiting amounts (2.5 ng) of 5' 6-FAM labelled 16-N_{603}-30 DNA. Concentrations 15.6-500 nM on the gel are shown for clarity. DNA was either methylated with meCpA (dashed) or meCpG (solid) at position 91 (red), position 81 (orange), or unmethylated (blue). Free DNA and complex bands are indicated, size markers in bp are shown. Quantification of the free DNA bands at each concentration, of the full concentration series (0.5-1000 nM), was fitted with a binding isotherm and an apparent dissociation constant ($K_{D\ app}$) calculated. Individual datapoints at each concentration represent a repeat. Full calculated $K_{D\ app}$ and hill slope statistics are summarised in Supplementary Table 3.

B. Representative EMSA native-PAGE showing a 2-fold dilution series of MBD (3 repeats) and MeCP2 (4 repeats) with limiting amounts (2.5 ng) of H2B T155C-OregonGreen488 labelled 16-N_{603}-30 nucleosomes. MBD concentrations 26.8-428 nM, and MeCP2 concentrations 56.7-287 nM, on the gel are shown for clarity. Nucleosomes were methylated with meCpA at position 91 (red), position 81 (orange), or unmethylated (blue). Quantification is shown in Figure 2C.

C. Representative EMSA native-PAGE (3 repeats) showing a 2-fold dilution series of MeCP2 with limiting amounts (2.5 ng) of H2B T155C-OregonGreen488 labelled 16-N_{603}-30 nucleosomes. Concentrations 56.7-287 nM on the gel are shown for clarity. Nucleosomes were methylated with meCpG at position 91 (red), position 81 (orange), or unmethylated (blue). Quantification is shown in Figure 2D.

Source data are provided as Source Data Files 3-4.

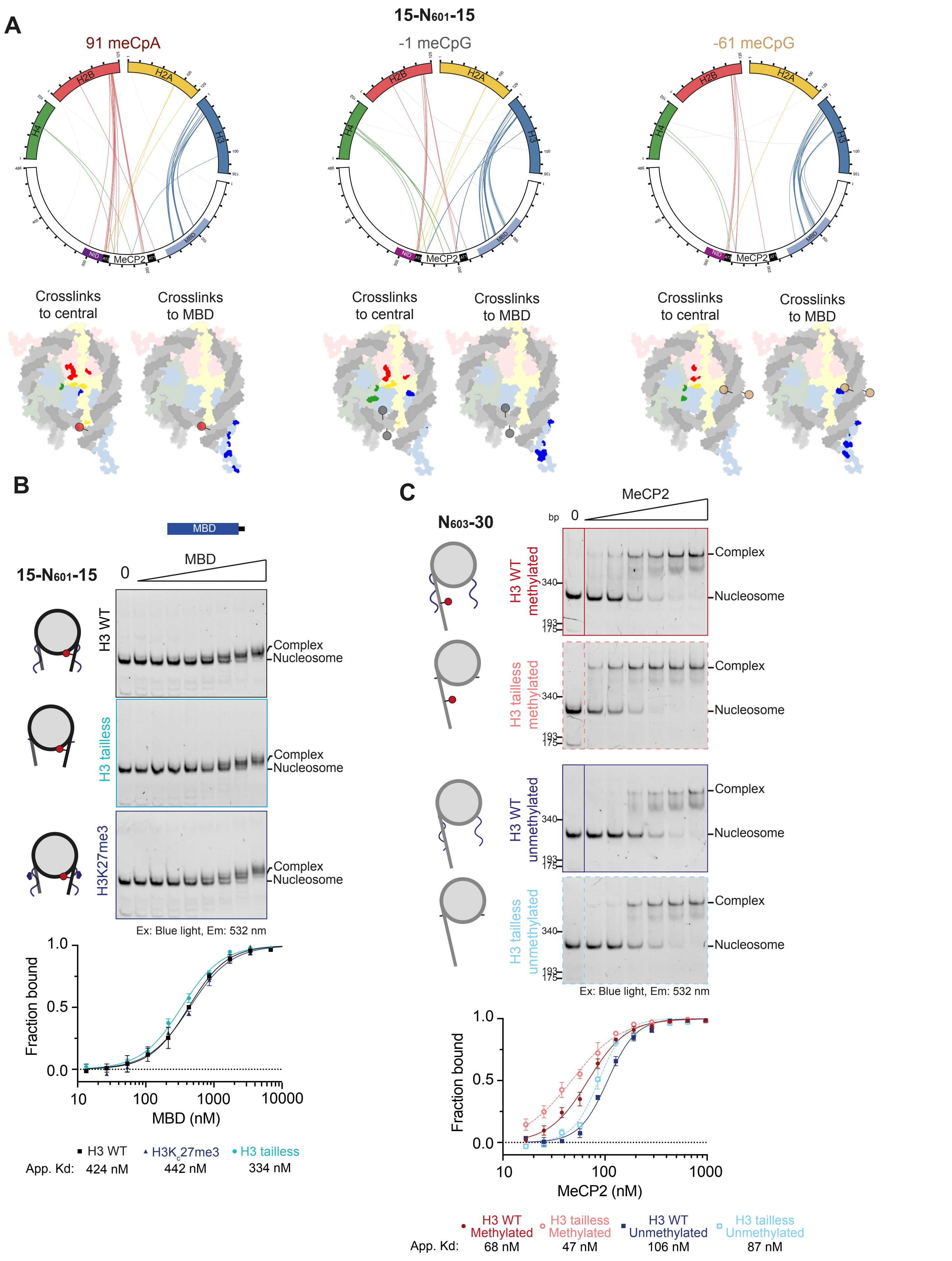

A
15-N601-15
91 meCpA
-1 meCpG
-61 meCpG
H2B
H2A
H4
H3
NID
MeCP2
MBD
Crosslinks to central
Crosslinks to MBD
B
MBD
15-N601-15
0
H3 WT
H3 tailless
H3K27me3
Complex
Nucleosome
Ex: Blue light, Em: 532 nm
Fraction bound
MBD (nM)
1.0
0.5
0.0
10
100
1000
10000
H3 WT
H3Kc27me3
H3 tailless
App. Kd:
424 nM
442 nM
334 nM
C
N603-30
MeCP2
bp
H3 WT methylated
H3 tailless methylated
H3 WT unmethylated
H3 tailless unmethylated
340
193
175
Complex
Nucleosome
Ex: Blue light, Em: 532 nm
Fraction bound
MeCP2 (nM)
1.0
0.5
0.0
10
100
1000
H3 WT Methylated
H3 tailless Methylated
H3 WT Unmethylated
H3 tailless Unmethylated
App. Kd:
68 nM
47 nM
106 nM
87 nM

Supplementary Figure 6: MeCP2 binds linker DNA alongside the H3 N-terminal tail.

A. Circular representation of Sulfo-SDA crosslinks between MeCP2 and histones on 15-N_{601}-15 nucleosomes containing 91 meCpA (left), -1 meCpG (middle), and -61 meCpG (right). Graphics of 15-N_{601}-15 nucleosomes (based on PDB:3LZ0) are shown below, with crosslinked histone residues highlighted by darker shading. Each graphic is split into crosslinks to the central region of MeCP2 (left) and crosslinks to the MBD (right).

B. Representative EMSA native-PAGE (3 repeats) showing a 2-fold dilution series of MBD (residues 77-167) with limiting amounts (2.5 ng) of 5' 6-FAM labelled 15-N_{601}-15 nucleosomes. Concentrations 13.4-1713 nM on the gel are shown for clarity. Nucleosomes were either wild-type (black), H3 tailless ($H3_{25\text{-}135}$ A25C) (light blue), or H3K27Cme3 (dark blue). All nucleosomes were methylated with meCpA at position 81. Free nucleosome and complex bands are indicated. Quantification of the free nucleosome bands at each concentration, of the full concentration series (13.4-27413 nM), was fitted with a binding isotherm and an apparent dissociation constant ($K_{D\ app}$) calculated. Error bars show standard error of the mean. Full calculated $K_{D\ app}$ and hill slope statistics are summarised in Supplementary Table 3.

C. Representative EMSA native-PAGE (3 repeats) showing a 1.5-fold dilution series of MeCP2 with limiting amounts (2.5 ng) of 5' 6-FAM labelled 16-N_{603}-30 nucleosomes. Concentrations 25.2-191 nM on the gel are shown for clarity. Nucleosomes were either wild-type with 91 meCpA (red), H3 tailless with 91 meCpA (light red), wild-type unmethylated (dark blue), or H3 tailless unmethylated (light blue). Free nucleosome and complex bands are indicated, size markers in bp are shown. Quantification of the full concentration series (16.8-2179 nM) was performed as described in B.

Source data are provided as Source Data Files 3-4.

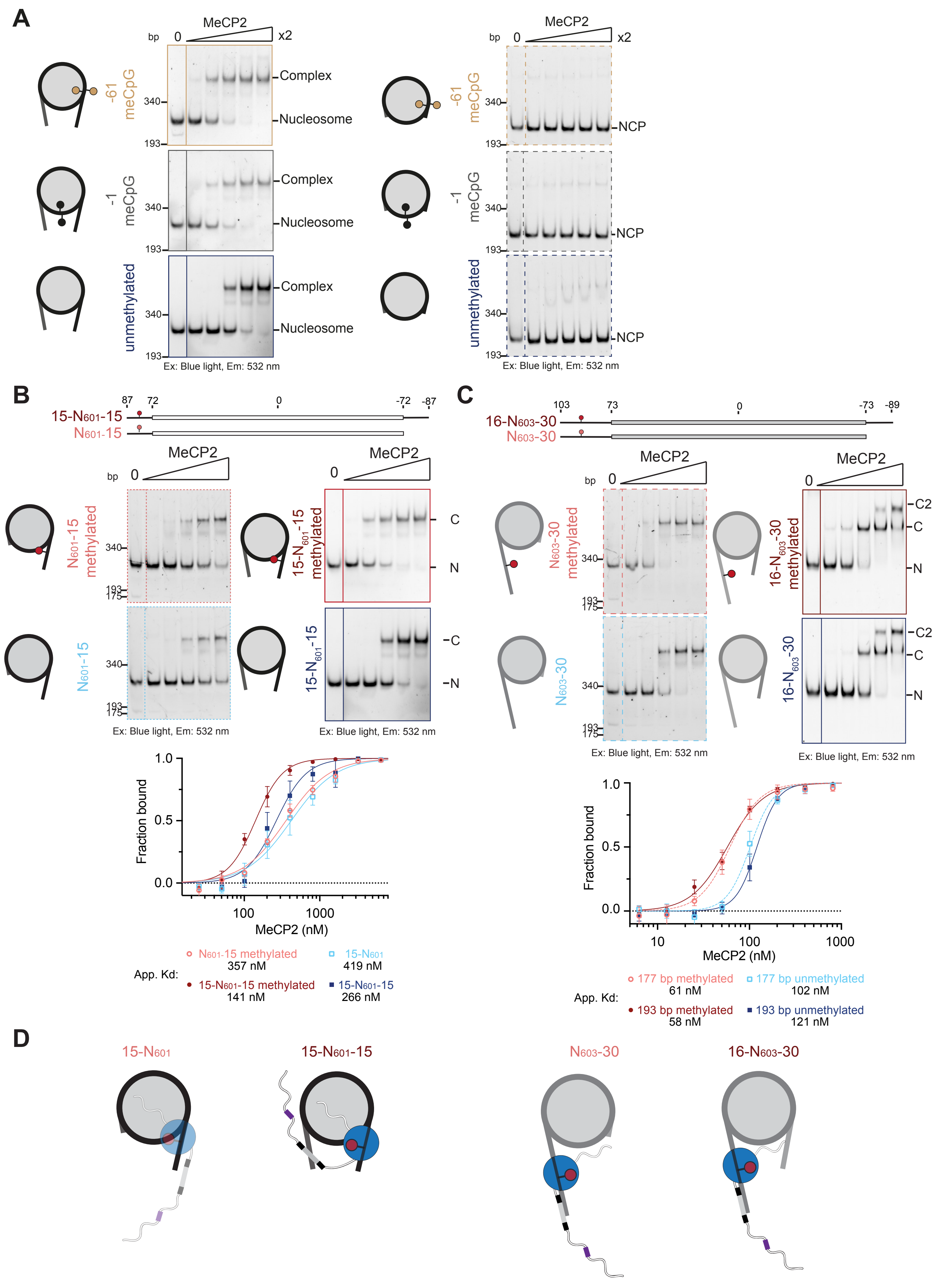
A
MeCP2
bp
0
x2
-61 meCpG
-1 meCpG
unmethylated
Complex
Nucleosome
NCP
340
193
Ex: Blue light, Em: 532 nm
B
15-N601-15
N601-15
87
72
0
-72
-87
N601-15 methylated
15-N601-15 methylated
C
N
175
Fraction bound
MeCP2 (nM)
App. Kd:
N601-15 methylated 357 nM
15-N601 419 nM
15-N601-15 methylated 141 nM
15-N601-15 266 nM
C
16-N603-30
N603-30
103
73
-73
-89
N603-30 methylated
16-N603-30 methylated
C2
177 bp methylated 61 nM
177 bp unmethylated 102 nM
193 bp methylated 58 nM
193 bp unmethylated 121 nM
D
15-N601
15-N601-15
N603-30
16-N603-30

Supplementary Figure 7: Nucleosome linker length specifies MeCP2 binding to nucleosomal DNA methylation.

A. Representative EMSA native-PAGE (3 repeats) showing a 2-fold dilution series of MeCP2 with limiting amounts (2.5 ng) of H2B T155C-OregonGreen488 labelled 15-N_{601}-15 nucleosomes and N_{601} NCPs. Concentrations 50.4-807 nM on the gel are shown for clarity. Nucleosomes were methylated with meCpG either at position -1 (grey) or -61 (brown), or unmethylated (blue). Quantification is shown in Figure 3A&B.

B. Representative EMSA native-PAGE (3 repeats) showing a 2-fold dilution series of MeCP2 with limiting amounts (2.5 ng) of H2B T115C-OregonGreen488 labelled 0-N_{601}-15 and 15-N_{601}-15 nucleosomes. Concentrations 50.4-807 nM on the gel are shown for clarity. Nucleosomes were methylated with meCpA at position 81 (pink/red), or unmethylated (light/dark blue). Free nucleosome and complex bands are indicated, size markers in bp are shown where included. Unmethylated 15-N_{601}-15 is repeated from A. Quantification of the free nucleosome bands at each concentration, of the full concentration series (6.3-12912 nM), was fitted with a binding isotherm and an apparent dissociation constant ($K_{D\ app}$) calculated. Error bars show standard error of the mean. Full calculated $K_{D\ app}$ and hill slope statistics are summarised in Supplementary Table 3.

C. Representative EMSA native-PAGE (3 repeats) showing a 2-fold dilution series of MeCP2 with limiting amounts (2.5 ng) of H2B T115C-OregonGreen488 labelled 0-N_{603}-30 and 16-N_{603}-30 nucleosomes. Concentrations 25.2-404 nM on the gel are shown for clarity. Nucleosomes were methylated with meCpA at position 91 (pink/red), or unmethylated (light/dark blue). Free nucleosome and complex bands are indicated, size markers in bp are shown where included. Quantification of the full concentration series (6.3-12912 nM) was performed as described in B.

D. A model for linker DNA dependent binding events of MeCP2 on nucleosomes. MeCP2 binds once to 15-N_{601}-15 nucleosomes. Both linkers are needed for optimal binding. While the MBD likely binds the meCpA site on one linker, a region of MeCP2 binding to the other linker is hypothesised. There are two binding events on 16-N_{603}-30 nucleosomes. MeCP2 first binds to the 30 bp linker containing a meCpA site, likely through an interaction with the MBD. The addition of a 16 bp second linker allows the DNA-mediated binding of a second MeCP2 at high protein concentrations.

Source data are provided as Source Data Files 3-4.

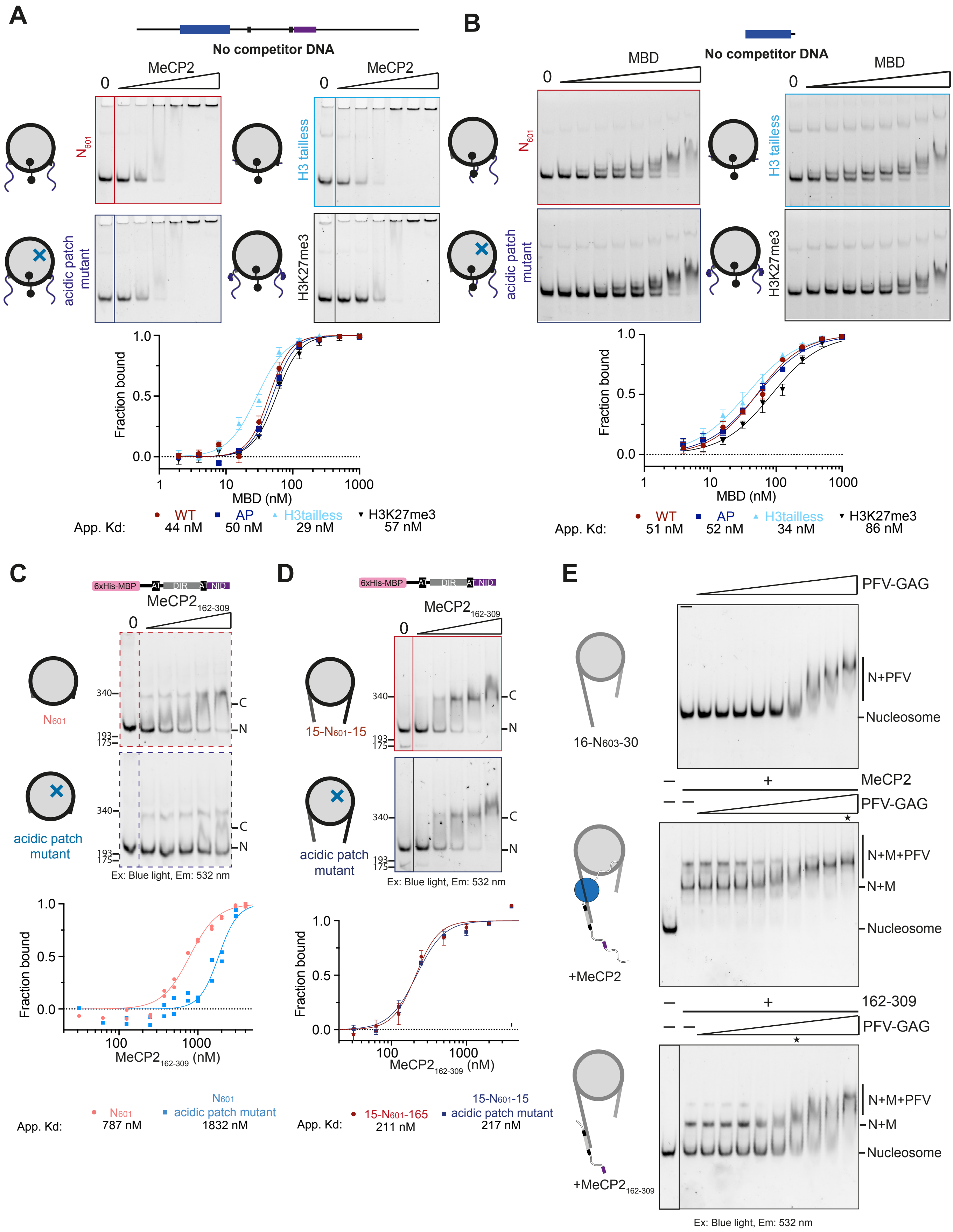
A
No competitor DNA
MeCP2
0
N601
H3 tailless
acidic patch mutant
H3K27me3
Fraction bound
MBD (nM)
WT
AP
H3tailless
H3K27me3
App. Kd:
44 nM
50 nM
29 nM
57 nM
B
MBD
No competitor DNA
51 nM
52 nM
34 nM
86 nM
C
6xHis-MBP
AT
DIR
NID
MeCP2162-309
340
193
175
C
N
N601
acidic patch mutant
Ex: Blue light, Em: 532 nm
MeCP2162-309 (nM)
787 nM
1832 nM
D
15-N601-15
15-N601-165
211 nM
217 nM
E
PFV-GAG
N+PFV
Nucleosome
16-N603-30
MeCP2
N+M+PFV
N+M
+MeCP2
162-309
+MeCP2162-309
Ex: Blue light, Em: 532 nm

Supplementary Figure 8: MeCP2 weakly contacts the nucleosome acidic patch.

A. Representative EMSA native-PAGE (3 repeats) showing a 2-fold dilution series of MeCP2 with limiting amounts (2.5 ng) of 5' 6-FAM labelled N_{601} nucleosome core particles. Concentrations 31.3-1000 nM on the gel are shown for clarity. Nucleosomes were either wild-type (WT) (red), acidic patch mutant (H2A E61/91/92A and H2B E113A) (blue), H3 tailless ($H3_{25\text{-}135}$ A25C) (light blue), or H3K27Cme3 (dark blue). Quantification of the free nucleosome bands at each concentration, of the full concentration series (1.95-4000 nM), was fitted with a binding isotherm and an apparent dissociation constant ($K_{D\ app}$) calculated. Error bars show standard error of the mean. Full calculated $K_{D\ app}$ and hill slope statistics are summarised in Supplementary Table 3. No competitor DNA was used in this assay.

B. Representative EMSA native-PAGE (3 repeats) showing a 2-fold dilution series of MBD (residues 77-167) with limiting amounts (2.5 ng) of 5' 6-FAM labelled N_{601} nucleosome core particles. Concentrations 3.9-500 nM on the gel are shown for clarity. Nucleosomes were either wild-type (WT) (red), acidic patch mutant (described in A) (blue), H3 tailless (described in A) (light blue), or H3K27Cme3 (dark blue). Quantification of the full concentration series (3.9-8000 nM) was performed as described in A. No competitor DNA was used in this assay.

C. Representative EMSA native-PAGE (2 repeats) showing a dilution series of HisMBP-$MeCP2_{162\text{-}309}$ with limiting amounts (2.5 ng) of 5' 6-FAM labelled N_{601} nucleosome core particles. Concentrations 375, 500, 750, 1000, 1500 nM on the gel are shown for clarity. Nucleosomes were either wild-type (WT) (red) or acidic patch mutant (described in A)(blue). Free nucleosome (n) and complex (c) bands are indicated, size markers in bp are shown. Quantification of the full concentration series (31.3-8000 nM) was performed as described in A. . Individual datapoints at each concentration represent a repeat.

D. Representative EMSA native-PAGE (3 repeats) showing a 2-fold dilution series of HisMBP-$MeCP2_{162\text{-}309}$ with limiting amounts (2.5 ng) of 5' 6-FAM labelled 15-N_{601}-15 nucleosomes. Concentrations 125-2000 nM on the gel are shown for clarity. Nucleosomes were either wild-type (WT) (red) or acidic patch mutant (described in A) (blue). Free nucleosome (n) and complex (c) bands are indicated, size markers in bp are shown. Quantification of the full concentration series (7.8-64000 nM) was performed as described in A.

E. EMSA native gel showing a two-fold dilution series (7.8-2000 nM) of acidic-patch binding PFV-GAG protein on 16-N_{603}-30 nucleosomes either alone (top), pre-bound with 2 μM of MeCP2 (middle), or pre-bound with 500 nM of HisMBP-$MeCP2_{162\text{-}309}$ (bottom). The lane with equimolar amounts of MeCP2 and PFV-GAG is marked (*), suggesting MeCP2 and PFV-GAG can co-exist on nucleosomes.

Source data are provided as Source Data Files 3-4.

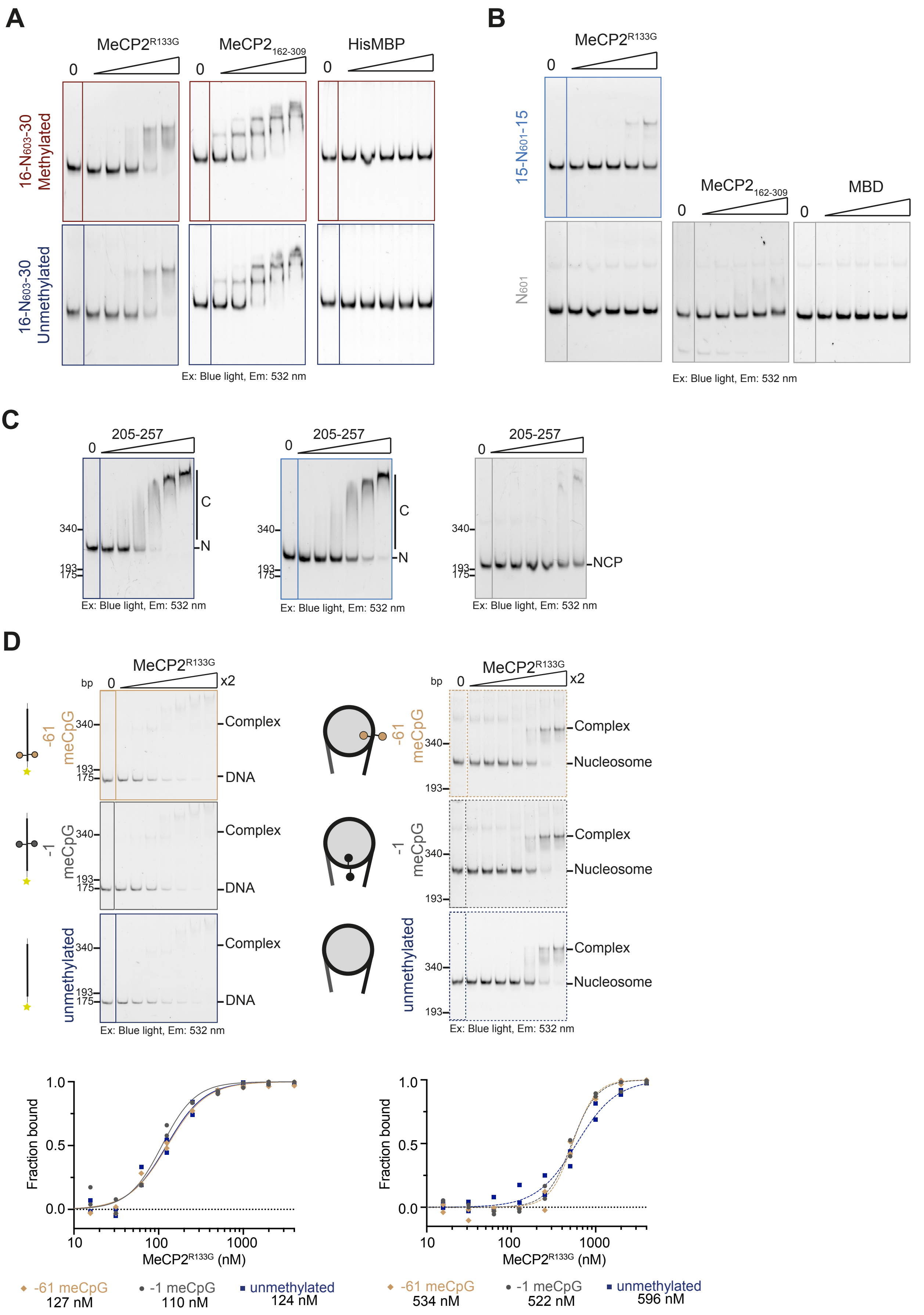
A
MeCP2R133G
MeCP2162-309
HisMBP
16-N603-30 Methylated
16-N603-30 Unmethylated
Ex: Blue light, Em: 532 nm
B
MeCP2R133G
15-N601-15
N601
MeCP2162-309
MBD
Ex: Blue light, Em: 532 nm
C
205-257
C
N
NCP
340
193
175
Ex: Blue light, Em: 532 nm
D
MeCP2R133G
bp
x2
-61 meCpG
-1 meCpG
unmethylated
Complex
DNA
Nucleosome
Ex: Blue light, Em: 532 nm
Fraction bound
MeCP2R133G (nM)
-61 meCpG 127 nM
-1 meCpG 110 nM
unmethylated 124 nM
-61 meCpG 534 nM
-1 meCpG 522 nM
unmethylated 596 nM

Supplementary Figure 9: MeCP2 constructs binding a panel of nucleosomes.

A. Representative EMSA native-PAGE (3 repeats) for the additional data plotted in Figure 4B showing a two-fold dilution series of MeCP2 R133G (27.2-435 nM) and MeCP2$_{162\text{-}309}$ (31.3-500 nM) construct with limiting amounts (2.5 ng) of H2B T155C-OregonGreen488 labelled 16-N$_{601}$-30 nucleosomes. Nucleosomes were either methylated with meCpA at position -91 (red) or unmethylated (blue). A control HisMBP EMSA was also performed (2 repeats) (31.3-500 nM).

B. Representative EMSA native-PAGE for the additional data plotted in Figure 4B showing a two-fold dilution series of MeCP2 R133G (27.2-435 nM) (3 repeats), MeCP2$_{162\text{-}309}$ (31.3-500 nM) (2 repeats) and MBD (26.8-428 nM) (3 repeats) constructs with limiting amounts (2.5 ng) of H2B T155C-OregonGreen488 labelled 15-N$_{601}$-15 (light blue) and N$_{601}$ (grey) unmethylated nucleosomes.

C. Representative EMSA native-PAGE (3 repeats) for the additional data plotted in Figure 5E showing a two-fold dilution series of MeCP2$_{205\text{-}257}$ (62.5-2000 nM) with limiting amounts (2.5 ng) of H2B T155C-OregonGreen488 labelled 16-N$_{603}$-30 (dark blue), 15-N$_{601}$-15 (light blue) and N$_{601}$ (grey) unmethylated nucleosomes.

D. Representative EMSA native-PAGE (2 repeats) showing a dilution series of MeCP2^{R133G} with limiting amounts (2.5 ng) of 5' 6-FAM labelled 15-N$_{601}$-15 DNA (solid), or H2B T115C-OregonGreen488 labelled 15-N$_{601}$-15 nucleosomes (dashed) . Concentrations 31.3-2000 nM on the gel are shown for clarity. DNA and nucleosomes were methylated with a single meCpG either -61 bp (brown) or -1 bp (grey) from the dyad, or unmethylated (blue). Free DNA and complex bands are indicated, size markers in bp are shown. Quantification of the free DNA bands at each concentration, of the full concentration series (7.8-16000 nM), was fitted with a binding isotherm and an apparent dissociation constant ($K_{D\ app}$) calculated. Individual datapoints at each concentration represent a repeat. Full calculated $K_{D\ app}$ and hill slope statistics are summarised in Supplementary Table 3.

Source data are provided as Source Data Files 3-4.

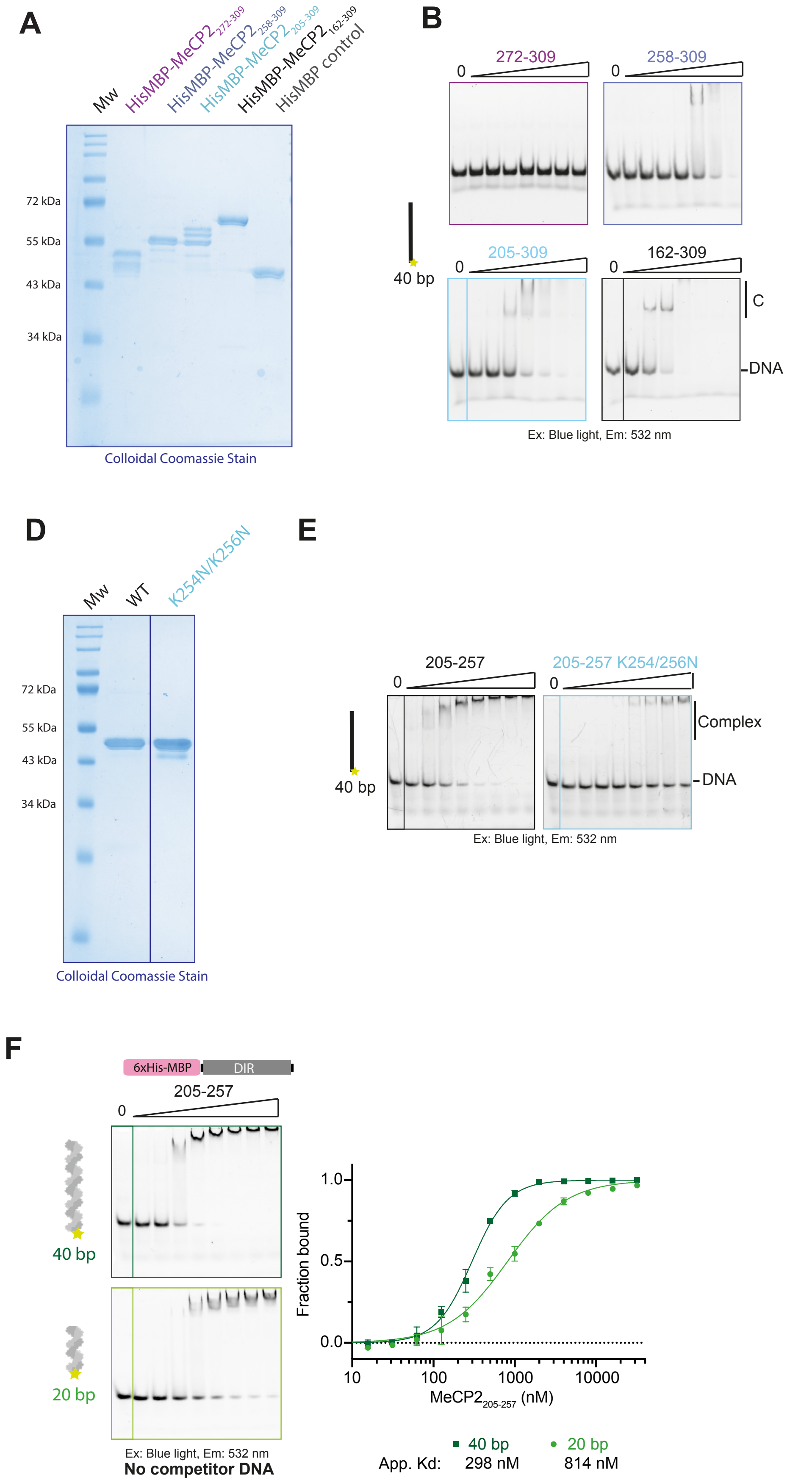
A
Mw
HisMBP-MeCP2 272-309
HisMBP-MeCP2 258-309
HisMBP-MeCP2 205-309
HisMBP-MeCP2 162-309
HisMBP control
72 kDa
55 kDa
43 kDa
34 kDa
Colloidal Coomassie Stain
B
272-309
258-309
205-309
162-309
0
40 bp
C
DNA
Ex: Blue light, Em: 532 nm
C
6xHis-MBP
HisMBP
0
40 bp
20 bp
Ex: Blue light, Em: 532 nm
No competitor DNA
D
Mw
WT
K254N/K256N
72 kDa
55 kDa
43 kDa
34 kDa
Colloidal Coomassie Stain
E
205-257
205-257 K254/256N
0
40 bp
Complex
DNA
Ex: Blue light, Em: 532 nm
F
6xHis-MBP
DIR
205-257
0
40 bp
20 bp
Ex: Blue light, Em: 532 nm
No competitor DNA
Fraction bound
1.0
0.5
0.0
10
100
1000
10000
MeCP2 205-257 (nM)
40 bp
20 bp
App. Kd:
298 nM
814 nM

Supplementary Figure 10: MeCP2 constructs binding to short DNA targets.

A. 12% SDS-PAGE gel loaded with 1 µg of each purified HisMBP tagged construct (Fig. 5).

B. Representative EMSA native-PAGE (3 repeats) showing a 2-fold dilution series of HisMBP tagged MeCP2 constructs (272-309 purple, 258-309 blue, 205-309 light blue, 162-309 black) with limiting amounts (2.5 ng) of 5' 6-FAM labelled 40 bp dsDNA. Concentrations 15.6-1000 nM on the gel are shown for clarity. Quantification is shown in Figure 5B. No competitor DNA was used in this assay.

C. Representative EMSA native-PAGE (2 repeats) showing a 2-fold dilution series of His-MBP tag alone control with limiting amounts (2.5 ng) of 5' 6-FAM labelled 40 bp dsDNA (dark green) and 20 bp dsDNA (light green). Concentrations 125-16000 nM on the gel are shown for clarity. Quantification of 40 bp binding is shown in Figure 5B. No competitor DNA was used in this assay.

D. 12% SDS-PAGE gel loaded with 1 µg of purified wild-type and K254N/K256N HisMBP-$MeCP2_{205\text{-}257}$ construct (Fig. 5).

E. Representative EMSA native-PAGE (3 repeats) showing a 2-fold dilution series of HisMBP tagged $MeCP2_{205\text{-}257}$ WT (black) and mutant (K254/256N) (light blue) with limiting amounts (2.5 ng) of 5' 6-FAM labelled 40 bp dsDNA. Concentrations 31.3-4000 nM on the gel are shown for clarity. Quantification is shown in Figure 5D. No competitor DNA was used in this assay.

F. Representative EMSA native-PAGE (3 repeats) showing a two-fold dilution series of HisMBP tagged $MeCP2_{205\text{-}257}$ on 5' 6-FAM labelled 40 bp dsDNA (dark green) and 20 bp dsDNA (light green). Concentrations 125-16000 nM on the gel are shown for clarity. Quantification of the free DNA bands at each concentration, of the full concentration series (7.8-32000 nM), was fitted with a binding isotherm and an apparent dissociation constant ($K_{D\ app}$) calculated. Error bars show standard error of the mean. Full calculated $K_{D\ app}$ and hill slope statistics are summarised in Supplementary Table 3. No competitor DNA was used in this assay.

Source data are provided as Source Data Files 3-4.

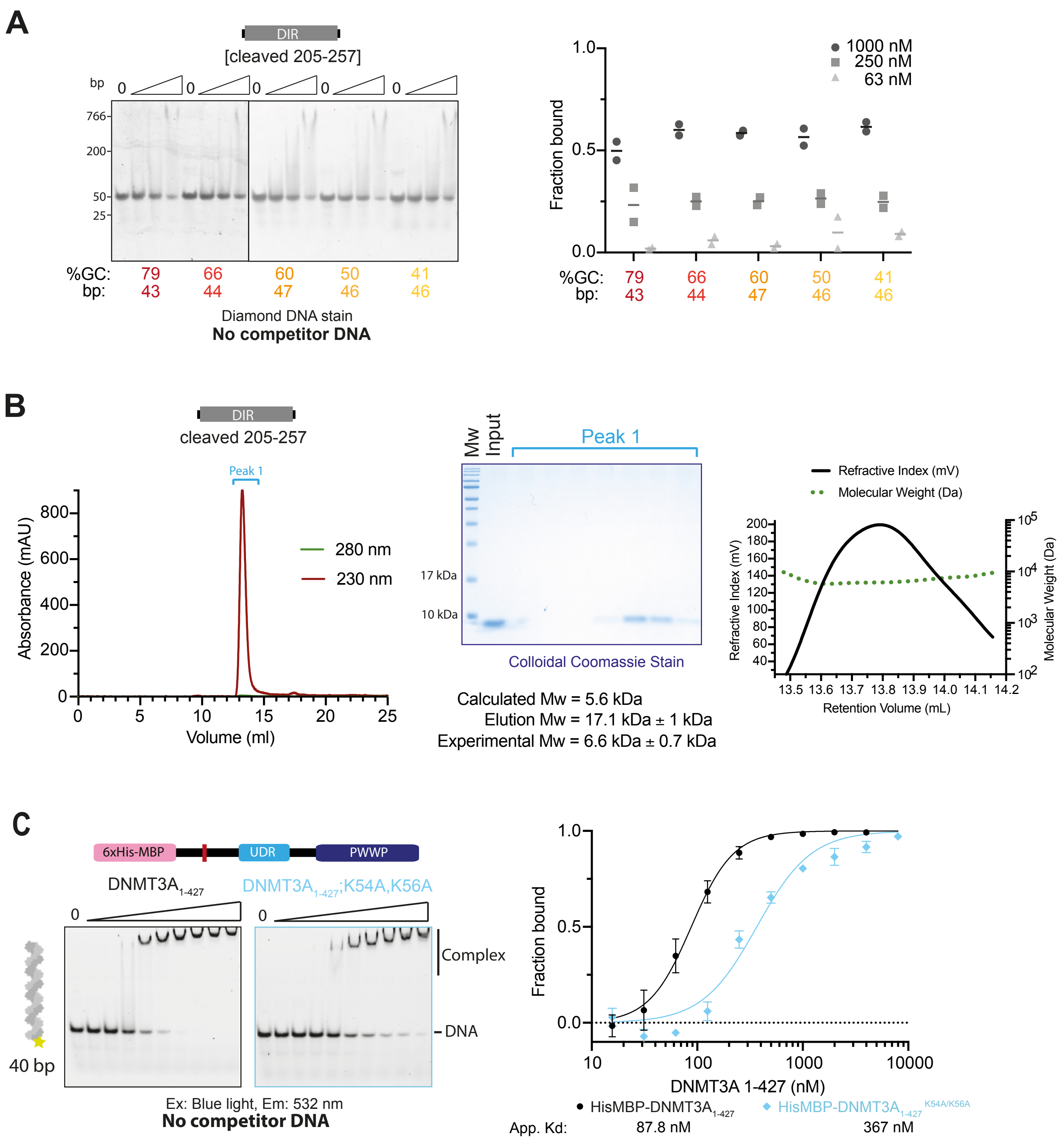
A
DIR
[cleaved 205-257]
bp
766
200
50
25
%GC: 79 66 60 50 41
bp: 43 44 47 46 46
Diamond DNA stain
No competitor DNA
1000 nM
250 nM
63 nM
Fraction bound
B
DIR
cleaved 205-257
Peak 1
Absorbance (mAU)
280 nm
230 nm
Volume (ml)
Mw
Input
17 kDa
10 kDa
Colloidal Coomassie Stain
Calculated Mw = 5.6 kDa
Elution Mw = 17.1 kDa ± 1 kDa
Experimental Mw = 6.6 kDa ± 0.7 kDa
Refractive Index (mV)
Molecular Weight (Da)
Retention Volume (mL)
C
6xHis-MBP
UDR
PWWP
DNMT3A1-427
DNMT3A1-427;K54A,K56A
Complex
DNA
40 bp
Ex: Blue light, Em: 532 nm
No competitor DNA
DNMT3A 1-427 (nM)
HisMBP-DNMT3A1-427
HisMBP-DNMT3A1-427 K54A/K56A
App. Kd: 87.8 nM 367 nM

Supplementary Figure 11: Further biochemical analysis of the DIR of MeCP2.

A. Representative EMSA native-PAGE (2 repeats) showing a 4-fold dilution series of HisMBP tagged $MeCP2_{205\text{-}257}$ binding to a panel of dsDNA with varied sequences. Concentrations 63, 250 and 1000 nM on the gel are shown. GC content (red-yellow) of each dsDNA target is annotated. Quantification of the free DNA bands at each concentration is shown as fraction bound. Bars show the mean. No competitor DNA was used in this assay.

B. (left) Size-exclusion chromatography trace of purified tag cleaved $MeCP2_{205\text{-}257}$. 280 nm (green) and 230 nm (red) traces are shown. Note that this construct does not absorb at 280 nm. Peak fractions were run on a 17 % SDS-PAGE gel and stained for protein (middle). Tag cleaved $MeCP2_{205\text{-}257}$ is 5.6 kDa. SEC-MALS analysis of the peak was also performed (right). Refractive index (mV) and calculated molecular weight (Da) are shown. Molecular weight (Mw) determined by amino acid composition (calculated), size-exclusion chromatography elution volume (elution apparent) and SEC-MALS measurement (experimental) are also shown below.

C. Representative EMSA native-PAGE (3 repeats) showing a 2-fold dilution series of His-MBP-$DNMT3A_{1\text{-}427}$ WT (black) and mutant (K54/56A) (light blue) with limiting amounts (2.5 ng) of 5' 6-FAM labelled 40 bp dsDNA. Concentrations 15.6-4000 nM on the gel are shown for clarity. Quantification of the free DNA bands at each concentration, of the full concentration series (15.6-8000 nM), was fitted with a binding isotherm and an apparent dissociation constant ($K_{D\ app}$) calculated. Error bars show standard error of the mean. Full calculated $K_{D\ app}$ and hill slope statistics are summarised in Supplementary Table 3. No competitor DNA was used in this assay.

Source data are provided as Source Data Files 3-4.

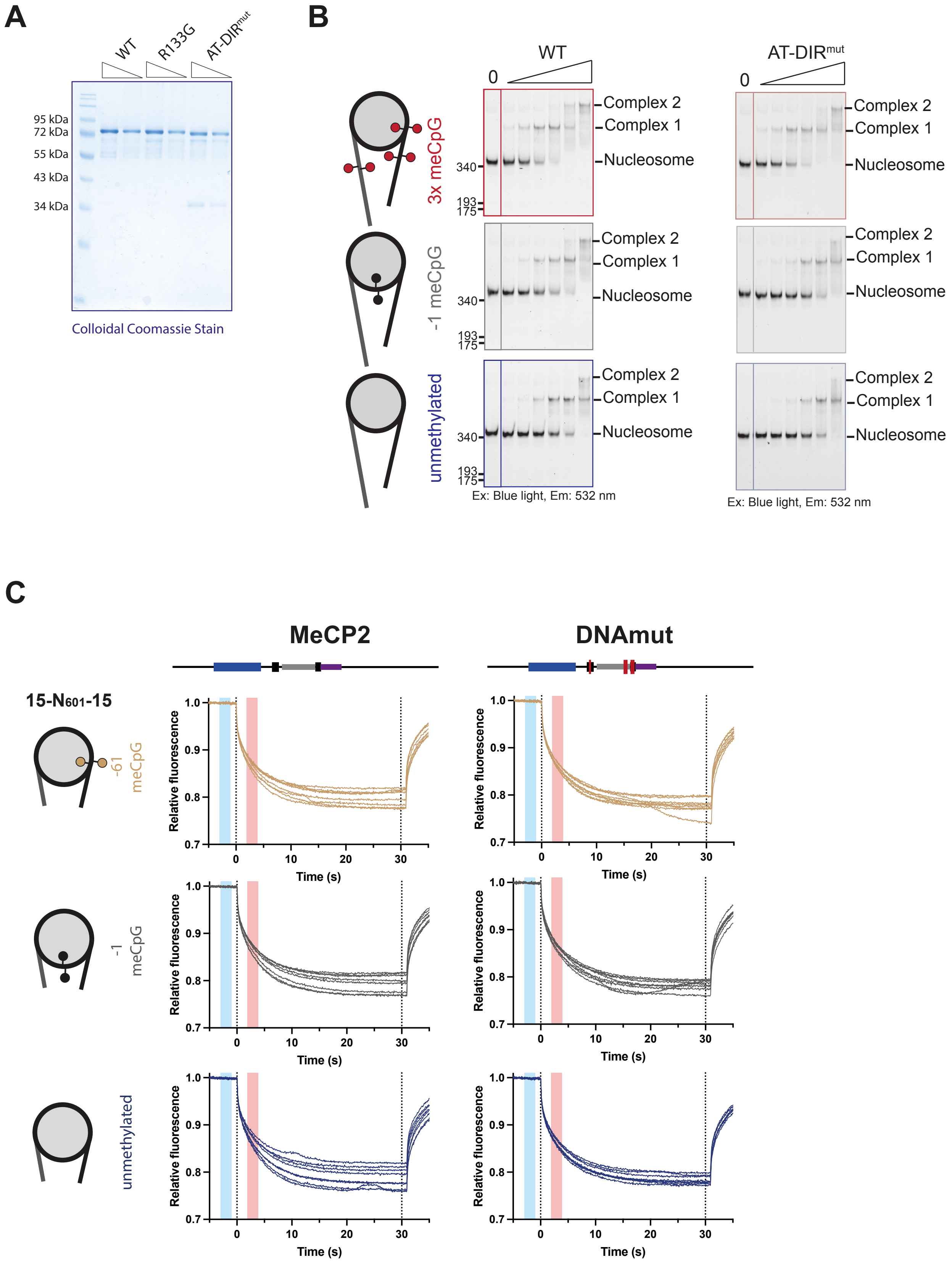
A
WT
R133G
AT-DIRmut
95 kDa
72 kDa
55 kDa
43 kDa
34 kDa
Colloidal Coomassie Stain
B
WT
AT-DIRmut
0
3x meCpG
-1 meCpG
unmethylated
Complex 2
Complex 1
Nucleosome
340
193
175
Ex: Blue light, Em: 532 nm
C
MeCP2
DNAmut
15-N601-15
-61 meCpG
-1 meCpG
unmethylated
Relative fluorescence
Time (s)

Supplementary Figure 12: AT-DIR^{mut} disrupts binding to nucleosomes.

A. 12% SDS-PAGE gel loaded with 0.5 and 0.25 µg of wild-type, R133G and AT-DIR^{mut} (R188G, R190G, K254N, K256N, R268Q) full-length MeCP2.

B. Representative EMSA native-PAGE (3 repeats) showing a 2-fold dilution series of MeCP2 wild-type (left) or AT-DIR^{mut} (R188G, R190G, K254N, K256N, R268Q) (right) with limiting amounts (2.5 ng) of H2B T115C-OregonGreen488 labelled 37-N_{601}-27 nucleosomes. Concentrations 3.9-2000 nM on the gel are shown for clarity. WT data was shown previously (Figure 1C). Nucleosomes were methylated with meCpG at three positions (+83, -61, -80 bp from the dyad) (red), a single meCpG (-1 bp from the dyad) (grey), or unmethylated (blue). Quantification is shown in Figure 6A.

C. Raw MST traces of MeCP2 WT and AT-DIR^{mut} (R188G, R190G, K254N, K256N, R268Q) titrated against H2AK119C-alexa647 labelled 15-N_{601}-15 nucleosomes. Cold (-3 to -1 s) and hot (1.9 to 3.9 s) regions are highlighted in blue and red. Initial (-5 to 0 s), thermophoresis (0 to 30 s), and recovery phases (30 to 35 s) are indicated by dotted lines.

Source data are provided as Source Data Files 3-4.

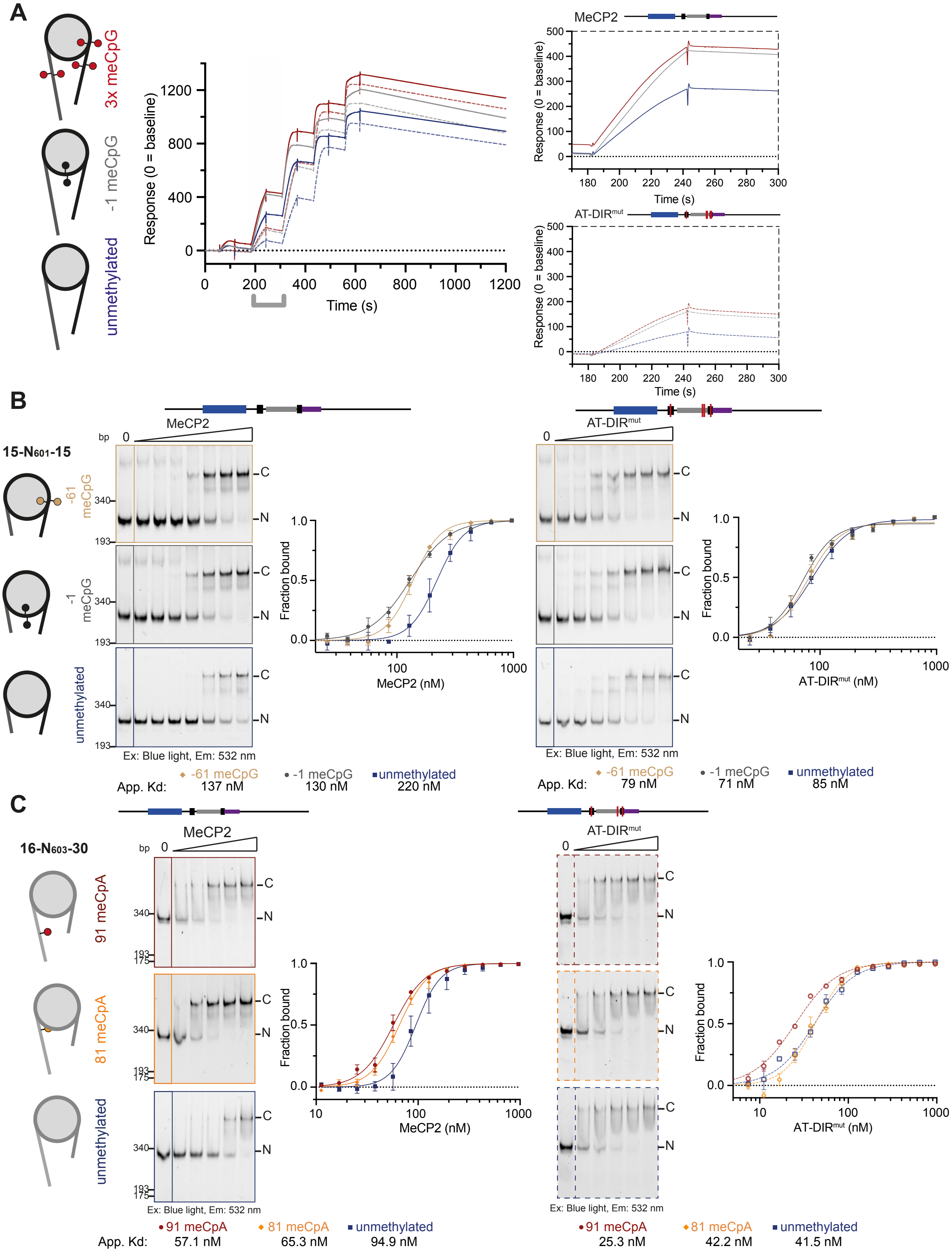
A
3x meCpG
-1 meCpG
unmethylated
Response (0 = baseline)
Time (s)
MeCP2
AT-DIR^mut
B
15-N601-15
-61 meCpG
-1 meCpG
unmethylated
MeCP2 (nM)
AT-DIR^mut (nM)
Fraction bound
Ex: Blue light, Em: 532 nm
App. Kd: -61 meCpG 137 nM; -1 meCpG 130 nM; unmethylated 220 nM
App. Kd: -61 meCpG 79 nM; -1 meCpG 71 nM; unmethylated 85 nM
C
16-N603-30
91 meCpA
81 meCpA
unmethylated
App. Kd: 91 meCpA 57.1 nM; 81 meCpA 65.3 nM; unmethylated 94.9 nM
91 meCpA 25.3 nM; 81 meCpA 42.2 nM; unmethylated 41.5 nM

Supplementary Figure 13: Disruption of DNA interactors in central MeCP2 disturbs binding to nucleosomes.

A. SPR trace of MeCP2 WT (solid) and AT-DIR^{mut} (R188G, R190G, K254N, K256N, R268Q) (dashed) binding to biotinylated 37-N_{601}-27 nucleosomes. Nucleosomes were methylated with meCpG at three positions (red), a single meCpG at position -1 bp (grey), or unmethylated (blue). A 2-fold concentration series of protein (12.5-200 nM), supplemented with 50 ng/µl, was injected sequentially. Comparisons of 25 nM protein association/dissociation phases (160-300 seconds) are enlarged (right).

B. Representative EMSA native-PAGE (3 repeats) showing a 1.5-fold dilution series of MeCP2 WT and AT-DIR^{mut} (R188G, R190G, K254N, K256N, R268Q) with limiting amounts (2.5 ng) of H2B T115C-OregonGreen488 labelled 15-N_{601}-15 nucleosomes. Wild-type concentrations 56.7-656 nM, and AT-DIR^{mut} concentrations 56.3-641 nM, on the gel are shown for clarity. Nucleosomes were methylated with meCpG either at position -61 (brown) position -1 (grey), or unmethylated (blue). Free nucleosome (n) and complex (c) bands are indicated, size markers in bp are shown where included. Quantification of the free nucleosome bands at each concentration, of the full concentration series (WT: 11.2-968 nM, AT-DIR^{mut}: 11.1-961 nM), was fitted with a binding isotherm and an apparent dissociation constant ($K_{D\ app}$) calculated. Error bars show standard error of the mean. Full calculated $K_{D\ app}$ and hill slope statistics are summarised in Supplementary Table 3.

C. Representative EMSA native-PAGE (3 repeats) showing a 1.5-fold dilution series of MeCP2 WT and AT-DIR^{mut} (R188G, R190G, K254N, K256N, R268Q) with limiting amounts (2.5 ng) of H2B T115C-OregonGreen488 labelled 16-N_{603}-30 nucleosomes. Wild-type concentrations 56.7-287 nM, and AT- DIR^{mut} concentrations 56.3-285 nM, on the gel are shown for clarity. WT data was shown previously (Supplementary Figure 5B). Nucleosomes were methylated with meCpA either at position 91 (red) position 81 (orange), or unmethylated (blue). Free nucleosome (n) and complex (c) bands are indicated, size markers in bp are shown where included. Quantification of the full concentration series (WT: 11.2-968 nM, AT-DIR^{mut}: 3.3-285 nM) was performed as described in B.

Source data are provided as Source Data Files 3-4.

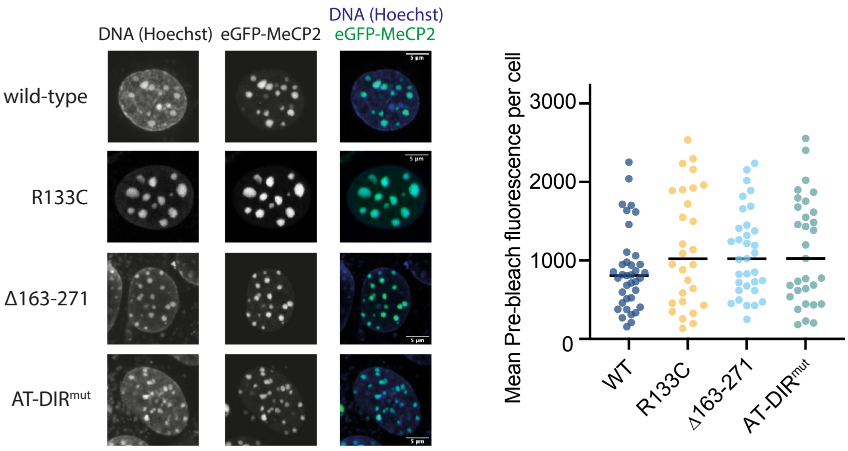

Supplementary Figure 14: MeCP2 constructs localise to pericentric heterochromatin foci in mouse fibroblasts.

(Left) Live cell imaging of NIH3T3 mouse fibroblast cells transfected with each eGFP labelled MeCP2 construct as indicated. Hoechst staining was used to visualise DNA. 5 μm scale bars are shown. (Right) Scatter plot of the mean fluorescence of each cell before bleaching and FRAP analysis. Median fluorescence of each construct is shown.

Source data are provided as Source Data Files 3.

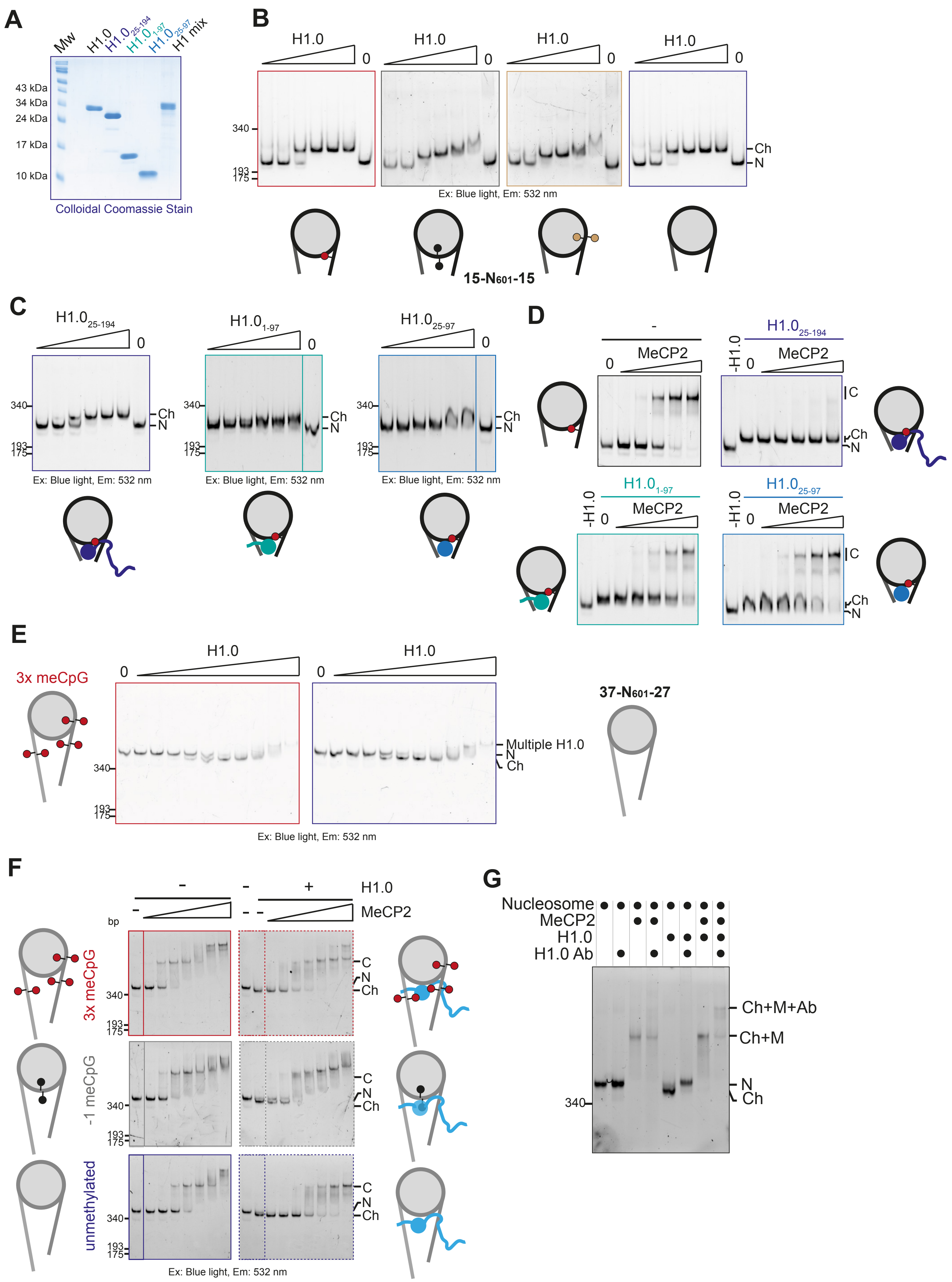

A
Mw
H1.0
H1.0 25-194
H1.0 1-97
H1.0 25-97
H1 mix
43 kDa
34 kDa
24 kDa
17 kDa
10 kDa
Colloidal Coomassie Stain
B
H1.0
0
340
193
175
Ch
N
Ex: Blue light, Em: 532 nm
15-N601-15
C
H1.0 25-194
H1.0 1-97
H1.0 25-97
D
-
MeCP2
-H1.0
C
E
3x meCpG
Multiple H1.0
37-N601-27
F
+
bp
3x meCpG
-1 meCpG
unmethylated
G
Nucleosome
MeCP2
H1.0
H1.0 Ab
Ch+M+Ab
Ch+M

Supplementary Figure 15: Formation of H1.0 chromatosomes.

A. SDS-PAGE loaded with 1 µg of each H1.0 construct.

B. EMSA native-PAGE showing a 2-fold dilution series (15.6-500 nM) of purified H1.0 protein on limiting amounts (2.5 ng) of H2B T115C-OregonGreen488 labelled 15-N_{601}-15 nucleosomes. Nucleosomes were either methylated with meCpA at position 81 (red), or with meCpG at position -1 (grey), -61 meCpG (brown), or unmethylated (blue). Free nucleosome (N) and chromatosome (Ch) bands are indicated, size markers in bp are shown.

C. EMSA native-PAGE showing a 2-fold dilution series (31.3-1000 nM) of H1.0 deletion proteins on limiting amounts (2.5 ng) of H2B T115C-OregonGreen488 labelled 15-N_{601}-15 nucleosomes with meCpA at position 81. Free nucleosome (N) and chromatosome (Ch) bands are indicated, size markers in bp are shown.

D. Representative EMSA native-PAGE (2 repeats) showing a 2-fold dilution series of MeCP2 on limiting amounts (2.5 ng) of H2B T115C-OregonGreen488 labelled 15-N_{601}-15 nucleosomes, or chromatosomes assembled with each H1.0 tail deletion construct as indicated. Concentrations 50.4-807 nM on the gel are shown for clarity. Nucleosomes were methylated with linker meCpA 81 bp from the dyad. Quantification is shown in Figure 7B.

E. EMSA native-PAGE showing a two-fold dilution series (3.9-2000 nM) of H1.0 protein on limiting amounts (2.5 ng) of H2B T115C-OregonGreen488 labelled 37-N_{601}-27 nucleosomes, Nucleosomes were methylated with meCpG at three positions (+83, -61, -80 bp from the dyad) (red), or unmethylated (blue). Free nucleosome (N), chromatosome (Ch), and multiple binding event bands are indicated, size markers in bp are shown.

F. Representative EMSA native-PAGE (3 repeats) showing a 2-fold dilution series of MeCP2 with limiting amounts (2.5 ng) of H2B T115C-OregonGreen488 labelled 37-N_{601}-27 nucleosomes. Chromatosomes were formed by pre-incubation with H1.0. Concentrations 25.2-1614 nM on the gel are shown for clarity. Nucleosomes were either methylated with meCpG at three positions (+83, -61, -80 bp from the dyad) (red), a single meCpG (-1 bp from the dyad) (grey), or unmethylated (blue). Quantification is shown in Figure 7C.

G. Super-shift EMSA native-PAGE probing H1 and MeCP2 coincidence on limiting amounts (2.5 ng) of H2B T115C-OregonGreen488 labelled nucleosomes. Nucleosomes were methylated with meCpG at three positions (+83, -61, -80 bp from the dyad) and bound by 100 nM of H1.0 (100 nM) and 250 nM of MeCP2 (250 nM). Polyclonal H1.0 antibody (*ab154111*) was added in a 1:25 dilution to induce super-shifting.

Source data are provided as Source Data Files 4.

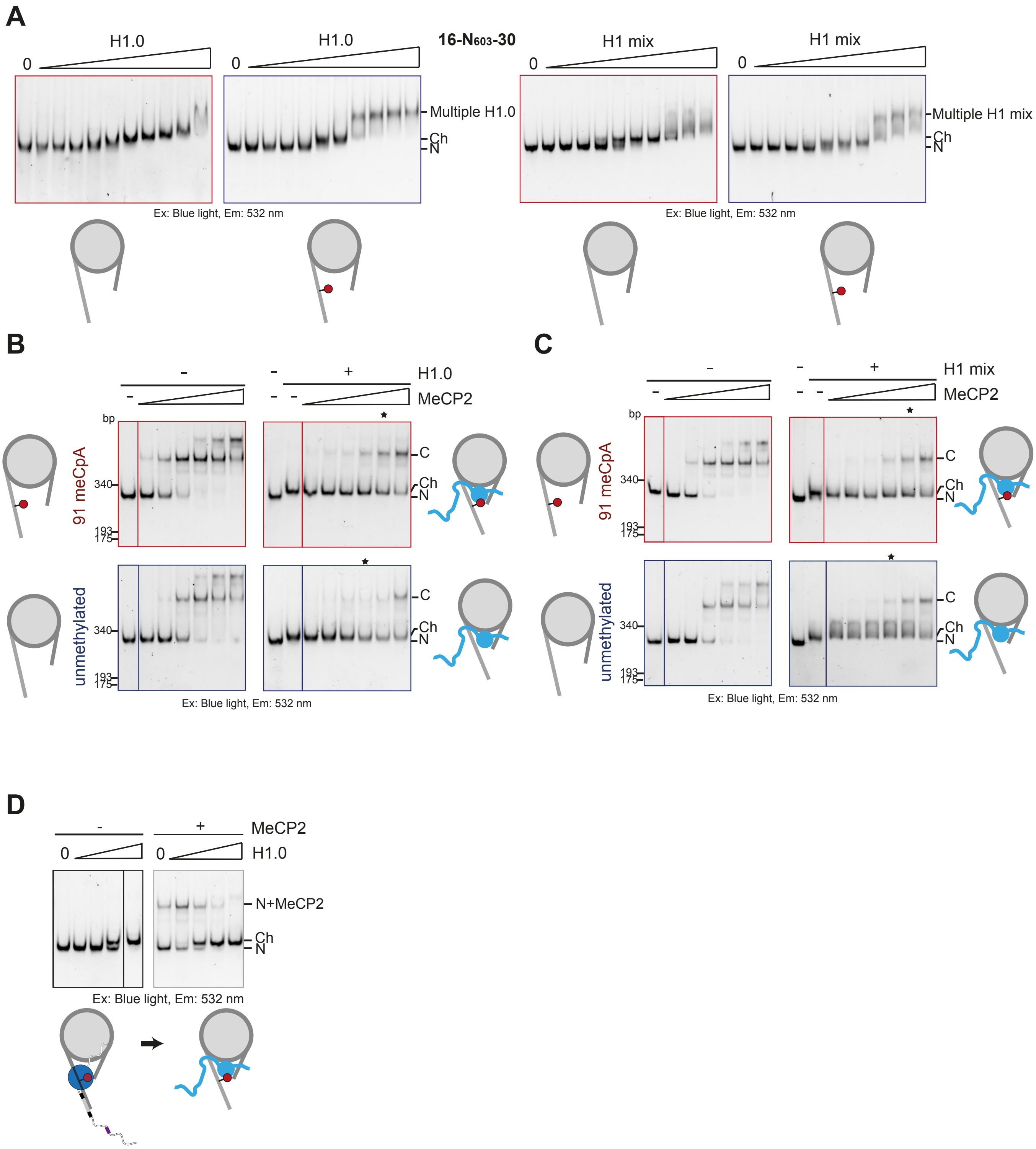
A
H1.0
0
H1.0
0
16-N603-30
H1 mix
0
H1 mix
0
Multiple H1.0
Ch
N
Multiple H1 mix
Ch
N
Ex: Blue light, Em: 532 nm
Ex: Blue light, Em: 532 nm
B
-
-
+
H1.0
-
- -
MeCP2
bp
91 meCpA
340
193
175
C
Ch
N
unmethylated
340
193
175
C
Ch
N
Ex: Blue light, Em: 532 nm
C
-
-
+
H1 mix
-
- -
MeCP2
bp
91 meCpA
340
193
175
C
Ch
N
unmethylated
340
193
175
C
Ch
N
Ex: Blue light, Em: 532 nm
D
-
+
MeCP2
0
0
H1.0
N+MeCP2
Ch
N
Ex: Blue light, Em: 532 nm

Supplementary Figure 16: H1 variants also compete with MeCP2 for nucleosome binding.

A. EMSA native-PAGE showing a 2-fold dilution series (3.9-2000 nM) of purified H1.0 protein (left) or commercially bought H1 mix protein (sigma-aldrich, 14-155) (right) on limiting amounts (2.5 ng) of H2B T115C-OregonGreen488 labelled 16-N_{601}-30 nucleosomes. Nucleosomes were either methylated with meCpA at position 91 (red) or unmethylated (blue). Free nucleosome (N) and chromatosome (Ch) bands are indicated, size markers in bp are shown.

B. EMSA native-PAGE (2 repeats) showing a 2-fold dilution series of MeCP2 on limiting amounts (2.5 ng) of H2B T115C-OregonGreen488 labelled 16-N_{601}-30 nucleosomes (left) or chromatosomes (right). Concentrations … nM on the gel are shown for clarity. Nucleosomes were either methylated with meCpA at position 91 (red), or unmethylated (blue). Free nucleosome (N), chromatosome (Ch), and complex bands are indicated, size markers in bp are shown. The concentration point at which MeCP2 and H1 were added in equimolar amounts is marked (*).

C. Repetition of (b) with commercial H1 mix.

D. EMSA native-PAGE showing a 2-fold dilution series (31.3-250 nM) of H1.0 on limiting amounts (2.5 ng) of H2B T115C-OregonGreen488 labelled 16-N_{603}-30 nucleosomes either alone (left), or pre-bound with 250 nM of MeCP2 (right). Free nucleosome (N), chromatosome (Ch), and complex bands are indicated.

Source data are provided as Source Data Files 4.

Supplementary Table 1: Expression constructs used in this study.

Constructs for MeCP2 *E.coli* expression	Source
MeCP2 FL	[3]
MeCP2 R133G	This study
MeCP2 K254N,K256N,R188G,R190G,R268Q	This study
MeCP2 77-167 6xHis	[4]
6xHisMBP MeCP2 162-309	This study
6xHisMBP MeCP2 205-257	This study
6xHisMBP MeCP2 205-257 K254N K256N	This study
6xHisMBP MeCP2 205-309	This study
6xHisMBP MeCP2 258-309	This study
6xHisMBP MeCP2 272-309	This study

Constructs for MeCP2 NIH3T3 expression	Source
MeCP2 FL	[5]
MeCP2 R133C	[6]
MeCP2 ∆163-271	This study
MeCP2 K254N,K256N,R188G,R190G,R268Q	This study

Constructs for histone *E.coli* expression	Source
H1.0 TEV-6xHis	This study
H1.0 1-97 TEV-6xHis	This study
H1.0 25-194 TEV-6xHis	This study
H1.0 25-194 TEV-6xHis	This study
unmodified H3.1 (No cys)	[7]
H3.1 K27C	[8]
H2A	Addgene
H2A E61A E91A E92A	[9]
H2A K119C	[10]
H2B	Addgene
H2B T155C	This study
H2B E113A	[9]
H4	Addgene
H3 A25C 25-135	[9]

Constructs for additional protein *E.coli* expression	Source
6xHisGST PFV GAG 534-557	[11]
6xHisMBP DNMT3A 1-427	[8]
6xHisMBP DNMT3A 1-427 K54A K56A	[12]

Constructs for nucleosomal DNA	Source
N_{601}	[11]
15-N_{601}-15	[10]
N_{601}-15	This study

37-N_{601}-27	[8]
37-$N_{601(mod)}$-27	This study
37-$N_{601(mod2)}$-27	This study
16-N_{603}-30	This study
N_{603}-30	This study
16-N_{603}-30 +81 CG	This study
16-N_{603}-30 +91 CG	This study

Supplementary Table 2: DNA sequences used for wrapping nucleosomes and binding assays

Name	Length	Sequence
N_{601}	145	CAGGATGTATATATCTGACACGTGCCTGGAGACTAGGGAGTAATCCCCTTGGCGGTTAAAACGCGGGGGACAGCGCGTCGTGCGTTTAAGCGGTGCTAGAGCTGTCTACGACCAATTGAGCGGCCTCGGCACCGGGATTCTCCA
15-N_{601}-15	175	ATGGAACACATTGCACAGGATGTATATATCTGACACGTGCCTGGAGACTAGGGAGTAATCCCCTTGGCGGTTAAAACGCGGGGGACAGCGCGTACGTGCGTTTAAGCGGTGCTAGAGCTGTCTACGACCAATTGAGCGGCCTCGGCACCGGGATTCTCCAGGGCGGCCGCGTATT
N_{601}-15	160	ATGGAACACATTGCACAGGATGTATATATCTGACACGTGCCTGGAGACTAGGGAGTAATCCCCTTGGCGGTTAAAACGCGGGGGACAGCGCGTACGTGCGTTTAAGCGGTGCTAGAGCTGTCTACGACCAATTGAGCGGCCTCGGCACCGGGATTCTCCA
37-N_{601}-27	209	GCTTCACCTCGTGACCCAAGCGACACCGGCACTGGGACAGGATGTATATATGTGACACGTGCCTGGAGACTAGGGAGTAATCCCCTTGGCGGTTAAAACGCGGGGGACAGCGCGTACGTGCGTTTAAGCGGTGCTAGAGCTGTCTACGACCAATTGAGCGGCCTCGGCACCGGGATTCTCCAGGGGATCCGGCATCATGGTCTAGAGCG
37-$N_{601(mod)}$-27	209	CGGTACCTCGCGAATGCATCTAATGGAACACATTGCACAGGATGTATATATCTGACACGTGCCTGGAGACTAGGGAGTAATCCCCTTGGCGGTTAAAACGCGGGGGACAGAACGCCGGTGCGTTTAAGCGGTGCTAGAGCTGTCTACGACCAATTGAGCGGCCTGCGCACAGGGATTCTCCAGGGCGGCCGCGTATTGGATCCCGAGCC
37-$N_{601(mod2)}$-27	209	CGGTACCTCGCGAATGCATCTAATGGAACACATTGCACAGGATGTATCCGGCTGACACGTGCCTGGAGACTAGGGAGTAATCCCCTTGGCGGTTAAAACGCGGGGGACAGAGCGTACGTGCGTTTAAGCGGTGCTAGAGCTGTCGCGCCCCAATTGAGCGGCCTCGGCACAGGGATTCTCCAGGGCGGCCGCGTATTGGATCCCGAGCC
16-N_{603}-30	193	GAGCCACAAAATCACCGACGCTCACGGGTGCCCAGTTCGCGCGCCCACCTACCGTGTGAAGTCGTCACTCGGGCTTCTAAGTACGCTTAGCGCACGGTAGAGCGCAATCCAAGGCTAACCACCGTGCATCGATGTTGAAAGAGGCCCTCCGTCCTTATTACTTCAAGTCCCTGGGGTACCCGTTTCACTGCCA
N_{603}-30	177	GAGCCACAAAATCACCGACGCTCACGGGTGCCCAGTTCGCGCGCCCACCTACCGTGTGAAGTCGTCACTCGGGCTTCTAAGTACGCTTAGCGCACGGTAGAGCGCAATCCAAGGCTAACCACCGTGCATCGATGTTGAAAGAGGCCCTCCGTCCTTATTACTTCAAGTCCCTGGGGT
16-N_{603}-30 +91 CG	193	GAGCCACAAAACCGGCGACGCTCACGGGTGCCCAGTTCGCGCGCCCACCTACCGTGTGAAGTCGTCACTCGGGCTTCTAAGTACGCTTAGCGCACGGTAGAGCGCAATCCAAGGCTAACCACCGTGCATCGATGTTGAAAGAGGCCCTCCGTCCTTATTACTTCAAGTCCCTGGGGTACCCGTTTCACTGCCA
16-N_{603}-30 +81 CG	193	GAGCCACAAAATCACCGACGCCCGGGGGTGCCCAGTTCGCGCGCCCACCTACCGTGTGAAGTCGTCACTCGGGCTTCTAAGTACGCTTAGCGCACGGTAGAGCGCAATCCAAGGCTAACCACCGTGCATCGATGTTGAAAGAGGCCCTCCGTCCTTATTACTTCAAGTCCCTGGGGTACCCGTTTCACTGCCA
20 bp	20	aatacgcggccgccCTGGAG
40 bp	40	aatacgcggccgccCTGGAGAATCCCGGTGCCGAGGCCGC
79% GC	43	GGAGGGCTCCCGGGGCCGGAACCGGGGTGGCTTGGGCTGGGAG
66% GC	44	GCGCAAGCGGGACGAGTGGAACGCACGCTGGAAAAGGGAGGCTG
60% GC (competitor DNA)	47	GGCTGGACACGGAAGCTTAAGCAAGGGAAATCTGGCCGCTCTGCTGG
50% GC	46	GACGCAGAGTTCGTCTGCGAACAAACCCTGAAGTACTTTCTTGGCA
40% GC	46	CGAAAATCTATACTTCCAAGGATGGTCACACCCCCAATTTGAAAAG

Supplementary Table 3: Summary of affinity measurements assessed by EMSA assays.

Protein construct	DNA length	Substrate type	Methylation state	K_D app			h		
FL MeCP2	37-N601-27	Nucleosome	3x meCpG	37.5	±	1.5	1.7	±	0.1
FL MeCP2	37-N601-27	Nucleosome	position -1 meCpG	72.1	±	3.2	1.8	±	0.1
FL MeCP2	37-N601-27	Nucleosome	unmethylated	137.4	±	6.4	2.1	±	0.2
FL MeCP2	37-$N_{601(mod)}$-27	Nucleosome	-56 meCpG	55.8	±	2.7	2.6	±	0.3
FL MeCP2	37-$N_{601(mod)}$-27	Nucleosome	-6 meCpG	57.4	±	2.5	2.1	±	0.2
FL MeCP2	37-$N_{601(mod)}$-27	Nucleosome	unmethylated	82.1	±	3.4	3.6	±	0.4
FL MeCP2	37-$N_{601(mod2)}$-27	Nucleosome	61 meCpG	62.0	±	3.0	2.2	±	0.2
FL MeCP2	37-$N_{601(mod2)}$-27	Nucleosome	-36 meCpG	70.8	±	5.0	2.0	±	0.2
FL MeCP2	37-$N_{601(mod2)}$-27	Nucleosome	unmethylated	91.9	±	5.4	2.8	±	0.4
FL MeCP2	15-N601-15	Nucleosome	position -1 meCpG	130.1	±	3.3	2.6	±	0.1
FL MeCP2	15-N601-15	Nucleosome	position -61 meCpG	137.4	±	3.8	3.7	±	0.3
FL MeCP2	15-N601-15	Nucleosome	unmethylated	220.5	±	7.5	3.7	±	0.4
FL MeCP2	15-N601-15	DNA	position -1 meCpG	45.3	±	2.0	1.9	±	0.1
FL MeCP2	15-N601-15	DNA	position -61 meCpG	50.5	±	1.9	2.2	±	0.2
FL MeCP2	15-N601-15	DNA	unmethylated	75.9	±	4.2	2.3	±	0.3
FL MeCP2	N601	Nucleosome	position -1 meCpG		N.D.			N.D.	
FL MeCP2	N601	Nucleosome	position -61 meCpG		N.D.			N.D.	
FL MeCP2	N601	Nucleosome	unmethylated		N.D.			N.D.	
FL MeCP2	16-N603-30	Nucleosome	position 91 meCpA	57.1	±	1.4	2.8	±	0.2
FL MeCP2	16-N603-30	Nucleosome	position 81 meCpA	65.3	±	3.3	3.1	±	0.4
FL MeCP2	16-N603-30	Nucleosome	unmethylated	94.9	±	2.7	3.6	±	0.3
FL MeCP2	16-N603-30	Nucleosome	position 91 meCpG	71.3	±	1.4	3.5	±	0.2
FL MeCP2	16-N603-30	Nucleosome	position 81 meCpG	65.5	±	1.6	2.9	±	0.2
FL MeCP2	16-N603-30	Nucleosome	unmethylated	137.7	±	4.0	3.4	±	0.3
FL MeCP2	16-N603-30	DNA	position 91 meCpA	27.8	±	1.9	1.9	±	0.2
FL MeCP2	16-N603-30	DNA	position 81 meCpA	27.1	±	1.8	1.9	±	0.2
FL MeCP2	16-N603-30	DNA	unmethylated	46.2	±	2.8	2.3	±	0.3
FL MeCP2	16-N603-30	DNA	position 91 meCpG	24.6	±	1.7	2.0	±	0.2
FL MeCP2	16-N603-30	DNA	position 81 meCpG	23.8	±	1.2	1.9	±	0.2
FL MeCP2	16-N603-30	DNA	unmethylated	45.4	±	2.6	2.6	±	0.3
FL MeCP2	15-N601-15	Nucleosome	position 81 meCpA	140.8	±	11.3	2.4	±	0.4
FL MeCP2	N601-15	Nucleosome	position 81 meCpA	357.1	±	19.7	1.5	±	0.1
FL MeCP2	N601-15	Nucleosome	unmethylated	419.0	±	42.8	1.5	±	0.2
FL MeCP2	N603-30	Nucleosome	position 91 meCpA	61.4	±	2.2	2.6	±	0.2
FL MeCP2	N603-30	Nucleosome	unmethylated	101.7	±	6.3	3.4	±	0.8
FL MeCP2	N603-30	Nucleosome, H3tailless	position 91 meCpA	47.0	±	1.1	1.8	±	0.1
FL MeCP2	N603-30	Nucleosome, H3tailless	unmethylated	86.9	±	2.3	3.4	±	0.3
FL MeCP2	N601	Nucleosome, no competitor	position -1 meCpG	44.2	±	1.8	2.7	±	0.3

FL MeCP2	N601	Nucleosome, AP mutant, no competitor	position -1 meCpG	49.7	±	1.6	2.6	±	0.2
FL MeCP2	N601	Nucleosome, H3tailless, no competitor	position -1 meCpG	29.2	±	1.3	2.0	±	0.1
FL MeCP2	N601	Nucleosome, H3K27me3, no competitor	position -1 meCpG	56.8	±	2.1	2.5	±	0.2
FL MeCP2	15-N601-15	H1.0 Chromatosome	position -1 meCpG		N.D.			N.D.	
FL MeCP2	15-N601-15	H1.0 Chromatosome	position -61 meCpG		N.D.			N.D.	
FL MeCP2	15-N601-15	H1.0 Chromatosome	unmethylated		N.D.			N.D.	
FL MeCP2	37-N601-27	H1.0 Chromatosome	3x meCpG meCpG	46.0	±	2.4	1.4	±	0.1
FL MeCP2	37-N601-27	H1.0 Chromatosome	position -1 meCpG	80.4	±	7.7	1.4	±	0.2
FL MeCP2	37-N601-27	H1.0 Chromatosome	unmethylated	164.5	±	16.8	2.2	±	0.4
FL MeCP2	15-N601-15	H1.0$_{25-194}$ Chromatosome	position 81 meCpA		N.D.			N.D.	
FL MeCP2	15-N601-15	H1.0$_{1-97}$ Chromatosome	position 81 meCpA	381.7	±	14.1	1.6	±	0.1
FL MeCP2	15-N601-15	H1.0$_{25-97}$ Chromatosome	position 81 meCpA	294.7	±	35.7	1.4	±	0.2
FL MeCP2 AT-DIR^{mut}	37-N601-27	Nucleosome	3x meCpG	42.5	±	1.8	1.8	±	0.1
FL MeCP2 AT-DIR^{mut}	37-N601-27	Nucleosome	position -1 meCpG	119.0	±	4.3	1.9	±	0.1
FL MeCP2 AT-DIR^{mut}	37-N601-27	Nucleosome	unmethylated	167.1	±	5.1	2.1	±	0.1
FL MeCP2 AT-DIR^{mut}	15-N601-15	Nucleosome	position -1 meCpG	71.3	±	2.3	3.3	±	0.3
FL MeCP2 AT-DIR^{mut}	15-N601-15	Nucleosome	position -61 meCpG	78.6	±	2.6	3.1	±	0.3
FL MeCP2 AT-DIR^{mut}	15-N601-15	Nucleosome	unmethylated	85.2	±	3.5	2.7	±	0.3
FL MeCP2 AT-DIR^{mut}	16-N603-30	Nucleosome	position 91 meCpA	25.3	±	0.6	1.8	±	0.1
FL MeCP2 AT-DIR^{mut}	16-N603-30	Nucleosome	position 81 meCpA	42.2	±	1.9	2.4	±	0.2
FL MeCP2 AT-DIR^{mut}	16-N603-30	Nucleosome	unmethylated	41.5	±	1.4	2.0	±	0.1
MBD	37-N601-27	Nucleosome	3x meCpG	67.9	±	4.6	1.0	±	0.1
MBD	37-N601-27	Nucleosome	position -1 meCpG	233.7	±	9.9	1.6	±	0.1
MBD	37-N601-27	Nucleosome	unmethylated	278.6	±	17.9	1.5	±	0.1
MBD	15-N601-15	Nucleosome	position -1 meCpG	812.5	±	55.7	1.6	±	0.1
MBD	15-N601-15	Nucleosome	position -61 meCpG	808.9	±	56.2	1.9	±	0.2
MBD	15-N601-15	Nucleosome	unmethylated	1001.9	±	62.9	1.6	±	0.1
MBD	N601	Nucleosome	unmethylated		N.D.			N.D.	
MBD	16-N603-30	Nucleosome	position 91 meCpA	85.7	±	6.1	1.0	±	0.1
MBD	16-N603-30	Nucleosome	position 81 meCpA	267.6	±	17.3	1.3	±	0.1
MBD	16-N603-30	Nucleosome	unmethylated	715.9	±	38.3	2.0	±	0.2
MBD	15-N601-15	Nucleosome	position 81 meCpA	424.4	±	14.4	1.5	±	0.1
MBD	15-N601-15	Nucleosome, H3tailless	position 81 meCpA	334.1	±	11.7	1.5	±	0.1
MBD	15-N601-15	Nucleosome, H3K27me3	position 81 meCpA	442.2	±	23.7	1.4	±	0.1
MBD	N601	Nucleosome, no competitor	position -1 meCpG	50.7	±	2.6	1.3	±	0.1
MBD	N601	Nucleosome, AP mutant, no competitor	position -1 meCpG	51.5	±	2.4	1.1	±	0.1

MBD	N601	Nucleosome, H3tailless, no competitor	position -1 meCpG	35.5	±	2.2	1.1	±	0.1
MBD	N601	Nucleosome, H3K27me3, no competitor	position -1 meCpG	85.5	±	6.0	1.2	±	0.1
FL MeCP2 R133G	16-N603-30	Nucleosome	position 91 meCpA	161.6	±	4.8	3.7	±	0.3
FL MeCP2 R133G	16-N603-30	Nucleosome	unmethylated	187.7	±	9.8	2.9	±	0.4
FL MeCP2 R133G	15-N601-15	Nucleosome	position -1 meCpG	522.4	±	20.6	2.9	±	0.3
FL MeCP2 R133G	15-N601-15	Nucleosome	position -61 meCpG	533.8	±	18.5	3.2	±	0.3
FL MeCP2 R133G	15-N601-15	Nucleosome	unmethylated	595.9	±	32.8	1.8	±	0.2
FL MeCP2 R133G	15-N601-15	DNA	position -1 meCpG	109.5	±	4.9	2.2	±	0.2
FL MeCP2 R133G	15-N601-15	DNA	position -61 meCpG	127.2	±	5.0	1.9	±	0.1
FL MeCP2 R133G	15-N601-15	DNA	unmethylated	124.3	±	6.0	1.9	±	0.2
FL MeCP2 R133G	N601	Nucleosome	unmethylated		N.D.			N.D.	
MeCP2 162-309	16-N603-30	Nucleosome	position 91 meCpA	102.9	±	2.7	1.9	±	0.1
MeCP2 162-309	16-N603-30	Nucleosome	unmethylated	94.6	±	3.4	2.7	±	0.2
MeCP2 162-309	15-N601-15	Nucleosome	unmethylated	239.6	±	11.8	2.8	±	0.4
MeCP2 162-309	N601	Nucleosome	unmethylated	734.3	±	53.4	2.6	±	0.4
MeCP2 162-309	15-N601-15	Nucleosome, AP mutant	unmethylated	217.0	±	9.3	2.4	±	0.2
MeCP2 162-309	N601	Nucleosome	unmethylated	786.5	±	28.0	2.5	±	0.2
MeCP2 162-309	N601	Nucleosome, AP mutant	unmethylated	1832.1	±	78.3	3.6	±	0.5
MeCP2 162-309	40 bp	DNA	unmethylated	31.6	±	1.7	2.9	±	0.4
MeCP2 205-257	16-N603-30	Nucleosome	unmethylated	298.1	±	9.5	2.6	±	0.2
MeCP2 205-257	15-N601-15	Nucleosome	unmethylated	581.2	±	21.1	2.1	±	0.1
MeCP2 205-257	N601	Nucleosome	unmethylated	1968.5	±	229.5	1.3	±	0.2
MeCP2 205-257	40 bp	DNA	unmethylated	215.5	±	9.2	1.5	±	0.1
MeCP2 205-257	20 bp	DNA	unmethylated	814.5	±	50.1	1.2	±	0.1
MeCP2 205-257 K254N K256N	40 bp	DNA	unmethylated		N.D.			N.D.	
MeCP2 272-309	40 bp	DNA	unmethylated		N.D.			N.D.	
MeCP2 258-309	40 bp	DNA	unmethylated	167.3	±	8.5	1.9	±	0.2
MeCP2 205-309	40 bp	DNA	unmethylated	77.5	±	4.8	2.6	±	0.4
DNMT3A 1-427	40 bp	DNA	unmethylated	87.8	±	5.2	2.2	±	0.2
DNMT3A 1-427 K54A K56A	40 bp	DNA	unmethylated	366.7	±	27.5	1.7	±	0.2
HisMBP control	16-N603-30	Nucleosome	position 91 meCpA		N.D.			N.D.	
HisMBP control	16-N603-30	Nucleosome	unmethylated		N.D.			N.D.	
HisMBP control	40 bp	DNA	unmethylated		N.D.			N.D.	
HisMBP control	20 bp	DNA	unmethylated		N.D.			N.D.	

References

1. Vasudevan, D., Chua, E.Y.D. & Davey, C.A. Crystal structures of nucleosome core particles containing the '601' strong positioning sequence. *J Mol Biol* **403**, 1-10 (2010).
2. Ho, K.L. et al. MeCP2 binding to DNA depends upon hydration at methyl-CpG. *Mol Cell* **29**, 525-31 (2008).
3. Klose, R.J. & Bird, A.P. MeCP2 behaves as an elongated monomer that does not stably associate with the Sin3a chromatin remodeling complex. *J Biol Chem* **279**, 46490-6 (2004).
4. Connelly, J.C. et al. Absence of MeCP2 binding to non-methylated GT-rich sequences in vivo. *Nucleic Acids Res* **48**, 3542-3552 (2020).
5. Klose, R.J. et al. DNA binding selectivity of MeCP2 due to a requirement for A/T sequences adjacent to methyl-CpG. *Mol Cell* **19**, 667-78 (2005).
6. Schmiedeberg, L., Skene, P., Deaton, A. & Bird, A. A temporal threshold for formaldehyde crosslinking and fixation. *PLoS One* **4**, e4636 (2009).
7. Wilson, M.D. et al. Retroviral integration into nucleosomes through DNA looping and sliding along the histone octamer. *Nat Commun* **10**, 4189 (2019).
8. Wapenaar, H. et al. The N-terminal region of DNMT3A engages the nucleosome surface to aid chromatin recruitment. *EMBO Rep* **25**, 5743-5779 (2024).
9. Belotserkovskaya, R. et al. PALB2 chromatin recruitment restores homologous recombination in BRCA1-deficient cells depleted of 53BP1. *Nat Commun* **11**, 819 (2020).
10. Burdett, H. et al. BRCA1-BARD1 combines multiple chromatin recognition modules to bridge nascent nucleosomes. *Nucleic Acids Res* **51**, 11080-11103 (2023).
11. Deak, G. et al. Histone divergence in trypanosomes results in unique alterations to nucleosome structure. *Nucleic Acids Res* (2023).
12. Wapenaar, H. et al. The N-terminal region of DNMT3A combines multiple chromatin reading motifs to guide recruitment. *bioRxiv*, 2023.10.29.564595 (2023).
