# Supplementary material for: MeCP2 requires interactions with nucleosome linker DNA to read chromatin DNA methylation
Source: Nat Commun. 2026 Apr 17;17:5374. doi: 10.1038/s41467-026-71741-0 (PMC13276077; doi:10.1038/s41467-026-71741-0)
Supplement: Supplementary file 2 — Reporting Summary [file 41467_2026_71741_MOESM2_ESM.pdf]

nature portfolio

Corresponding author(s): Marcus Wilson & Adrian Bird

Last updated by author(s): Oct 3, 2025

# Reporting Summary

Nature Portfolio wishes to improve the reproducibility of the work that we publish. This form provides structure for consistency and transparency in reporting. For further information on Nature Portfolio policies, see our Editorial Policies and the Editorial Policy Checklist.

## Statistics

For all statistical analyses, confirm that the following items are present in the figure legend, table legend, main text, or Methods section.

| n/a | Confirmed | |
|---|---|---|
| ☐ | ☒ | The exact sample size (*n*) for each experimental group/condition, given as a discrete number and unit of measurement |
| ☐ | ☒ | A statement on whether measurements were taken from distinct samples or whether the same sample was measured repeatedly |
| ☐ | ☒ | The statistical test(s) used AND whether they are one- or two-sided<br>*Only common tests should be described solely by name; describe more complex techniques in the Methods section.* |
| ☒ | ☐ | A description of all covariates tested |
| ☐ | ☒ | A description of any assumptions or corrections, such as tests of normality and adjustment for multiple comparisons |
| ☐ | ☒ | A full description of the statistical parameters including central tendency (e.g. means) or other basic estimates (e.g. regression coefficient) AND variation (e.g. standard deviation) or associated estimates of uncertainty (e.g. confidence intervals) |
| ☐ | ☒ | For null hypothesis testing, the test statistic (e.g. *F*, *t*, *r*) with confidence intervals, effect sizes, degrees of freedom and *P* value noted<br>*Give P values as exact values whenever suitable.* |
| ☒ | ☐ | For Bayesian analysis, information on the choice of priors and Markov chain Monte Carlo settings |
| ☒ | ☐ | For hierarchical and complex designs, identification of the appropriate level for tests and full reporting of outcomes |
| ☒ | ☐ | Estimates of effect sizes (e.g. Cohen's *d*, Pearson's *r*), indicating how they were calculated |

*Our web collection on statistics for biologists contains articles on many of the points above.*

## Software and code

Policy information about availability of computer code

| | |
|---|---|
| Data collection | ImageLab Touch Software version 3.0.1.14 for gel imaging, Zeiss ZEN software (black edition) for microscopy, AcquireMP software version 2024 R2.1 for mass photometry, MO.Control version 1.6 for MST, Biacore T200 software version 3.2 for SPR, ChromLab version 6.1.27.0 for chromatography traces, UCSF ChimeraX version 1.7.1 (Meng et al., 2023) for structural images and SASA data. |
| Data analysis | ImageLab version 6.1 for gel quantification, Thermofisher Connect Microsatellite analysis software for creating footprinting images, GraphPad Prism version 10.6.0 for graph generation and statistical analysis, PlotProtein (Turner., 2013) for plotting ClinVar mutations, DiscoverMP version 2024 R2.1 for mass photometry, MO.affinity analysis version 2.3 for MST data, OMNISEC software version 5.1 for SEC-MALS, Fiji based on ImageJ version 1.53 (Schindelin et al., 2012) and a custom script for FRAP analysis (https://doi.org/10.5281/zenodo.2654601), ProteoWizard version 3.0.24283 and Xi software version 1.8.4.1 for crosslinking mass spectrometry. |

For manuscripts utilizing custom algorithms or software that are central to the research but not yet described in published literature, software must be made available to editors and reviewers. We strongly encourage code deposition in a community repository (e.g. GitHub). See the Nature Portfolio guidelines for submitting code & software for further information.

# Data

Policy information about availability of data

All manuscripts must include a data availability statement. This statement should provide the following information, where applicable:

- Accession codes, unique identifiers, or web links for publicly available datasets
- A description of any restrictions on data availability
- For clinical datasets or third party data, please ensure that the statement adheres to our policy

Source Data are provided with this paper. The authors declare that all other data supporting the findings of this study are available within the paper and its supplementary and source information files. Crosslinking mass spectrometry data has been uploaded to PRIDE database under identifier PXD064826. Protein structures used for modelling are available on the PDB , accession codes 3c2i (https://doi.org/10.2210/pdb3C2I/pdb) and 3LZ0 (https://doi.org/10.2210/pdb3LZ0/pdb). The script for FRAP analysis using a custom macro with Fiji software is available on Zenodo (https://doi.org/10.5281/zenodo.2654601).

# Research involving human participants, their data, or biological material

Policy information about studies with human participants or human data. See also policy information about sex, gender (identity/presentation), and sexual orientation and race, ethnicity and racism.

| | |
|---|---|
| Reporting on sex and gender | Not applicable, this study does not involve human participants or cell lines. |
| Reporting on race, ethnicity, or other socially relevant groupings | Not applicable, this study does not involve human participants or cell lines. |
| Population characteristics | Not applicable, this study does not involve human participants or cell lines. |
| Recruitment | Not applicable, this study does not involve human participants or cell lines. |
| Ethics oversight | Not applicable, this study does not involve human participants or cell lines. |

Note that full information on the approval of the study protocol must also be provided in the manuscript.

# Field-specific reporting

Please select the one below that is the best fit for your research. If you are not sure, read the appropriate sections before making your selection.

☒ Life sciences ☐ Behavioural & social sciences ☐ Ecological, evolutionary & environmental sciences

For a reference copy of the document with all sections, see nature.com/documents/nr-reporting-summary-flat.pdf

# Life sciences study design

All studies must disclose on these points even when the disclosure is negative.

| | |
|---|---|
| Sample size | Biochemical experiment concentration ranges were determined empirically based on initial/previous experiments to cover the full binding isotherm. For EMSAs the number of concentration points were limited by the number of wells available per gel. Two control samples and a size ladder were run per gel. For MST the number of concentration points were limited by the number of capillaries available to be simultaneously loaded.<br><br>FRAP experiments sample size was not predetermined. We acquired the maximum number of images possible per microscopy session. Three sessions were performed, each with an independent transfection. |
| Data exclusions | For EMSA experiments, lanes with abnormal (spread or misshapen, preventing accurate quantification) or degraded bands were excluded. For FRAP experiments, bleached spots that had moved out of focus during live cell imaging were discarded. Spots with very high fluorescence prior to bleaching, compared to the mean, were also discarded. |
| Replication | Biochemical experiments were independently replicated at least two times, typically more, with exact numbers given in each figure legend. For FRAP experiments three independent transfection and data collection sessions were performed for each sample. |
| Randomization | Randomisation was not applicable as there was no grouping in this study. |
| Blinding | Blinding was not applicable as there were no prior assumptions about experimental outcomes, and no clinical trials or population studies were conducted. |

# Reporting for specific materials, systems and methods

We require information from authors about some types of materials, experimental systems and methods used in many studies. Here, indicate whether each material, system or method listed is relevant to your study. If you are not sure if a list item applies to your research, read the appropriate section before selecting a response.

## Materials & experimental systems

| n/a | Involved in the study | |
|---|---|---|
| ☐ | ☒ | Antibodies |
| ☐ | ☒ | Eukaryotic cell lines |
| ☒ | ☐ | Palaeontology and archaeology |
| ☒ | ☐ | Animals and other organisms |
| ☒ | ☐ | Clinical data |
| ☒ | ☐ | Dual use research of concern |
| ☒ | ☐ | Plants |

## Methods

| n/a | Involved in the study | |
|---|---|---|
| ☒ | ☐ | ChIP-seq |
| ☒ | ☐ | Flow cytometry |
| ☒ | ☐ | MRI-based neuroimaging |

# Antibodies

| | |
|---|---|
| Antibodies used | Anti histone H1.0 (rabbit polyclonal antibody): Abcam ab154111, Lot: GR3413078-6. Used at a 1:25 dilution. |
| Validation | This antibody recognises a fragment within human H1.0, for manufacturers validation see: https://www.abcam.com/en-us/products/primary-antibodies/histone-h10-antibody-ab154111 |

# Eukaryotic cell lines

Policy information about cell lines and Sex and Gender in Research

| | |
|---|---|
| Cell line source(s) | NIH 3T3 (Mus musculus, Sex: Male, Source: ATCC CRL-1658) |
| Authentication | For manufacturers authentication see: https://www.atcc.org/products/crl-1658. The identity of the cell line was verified by Sanger sequencing of mitochondrial Cytochrome b and compared to reference sequences (Ensembl, NCBI). |
| Mycoplasma contamination | All cell lines were tested negative for Mycoplasma contamination. |
| Commonly misidentified lines (See ICLAC register) | No commonly misidentified lines were used. |

# Plants

| | |
|---|---|
| Seed stocks | Not applicable |
| Novel plant genotypes | Not applicable |
| Authentication | Not applicable |
