# Supplementary material for: MeCP2 requires interactions with nucleosome linker DNA to read chromatin DNA methylation
Source: Nat Commun. 2026 Apr 17;17:5374. doi: 10.1038/s41467-026-71741-0 (PMC13276077; doi:10.1038/s41467-026-71741-0)

Figure 1B

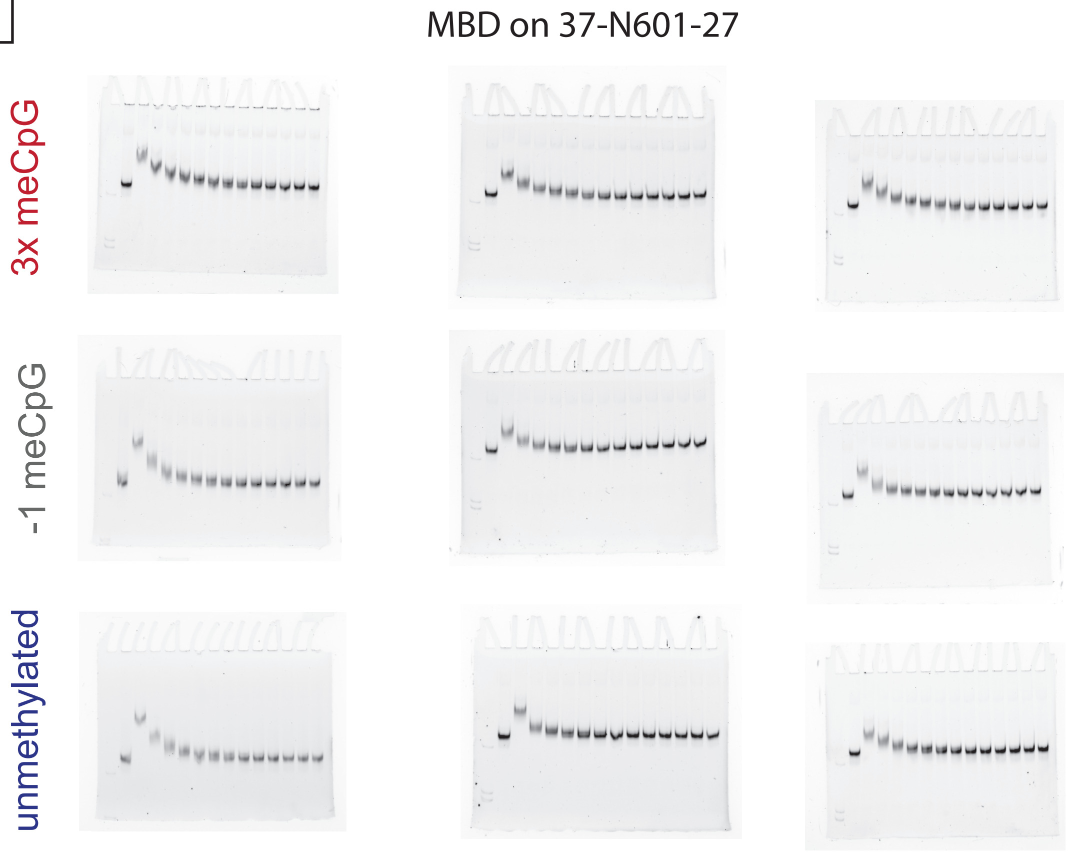

Figure 1C

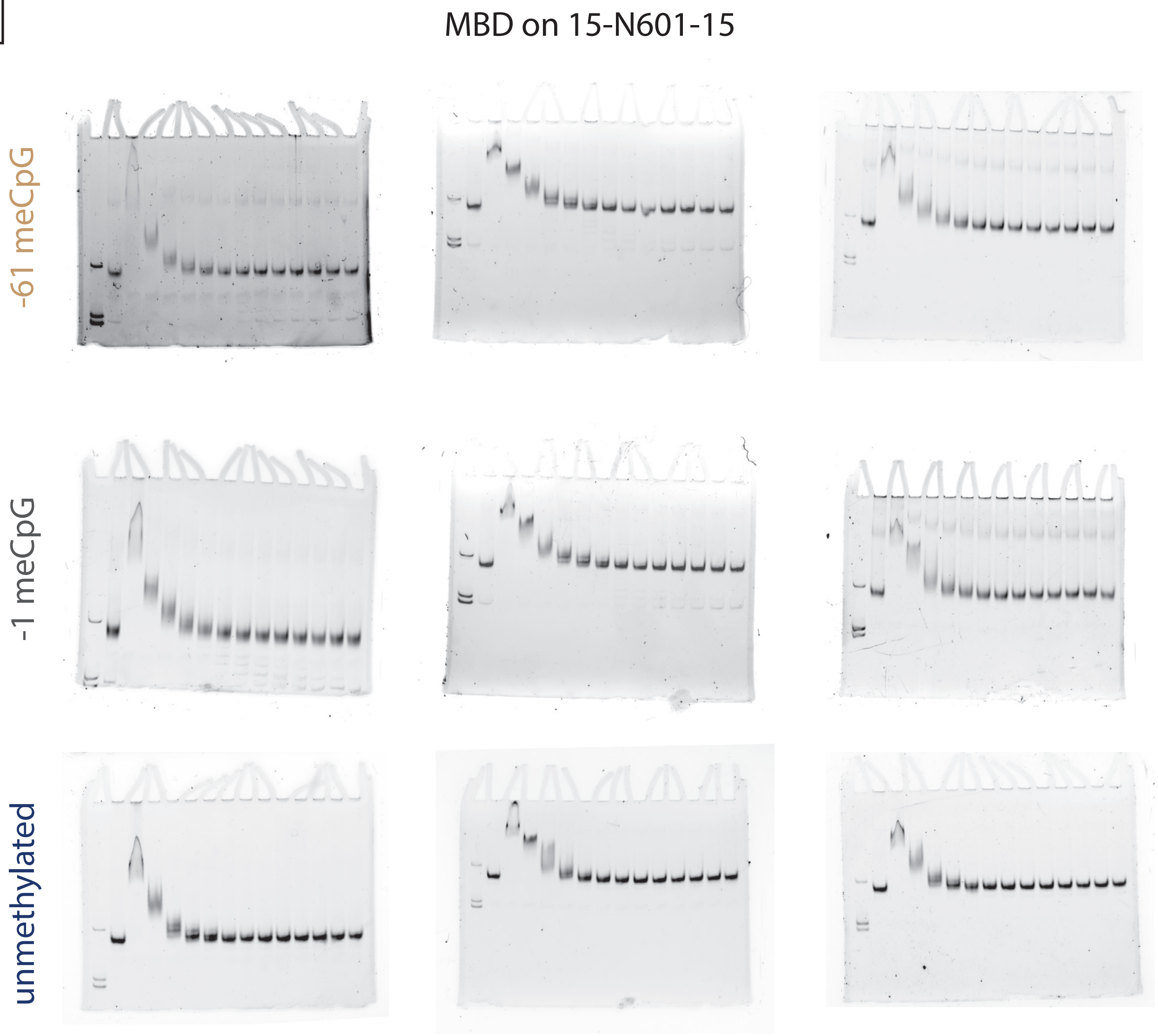

Figure 1D

MeCP2 on 37-N601-27

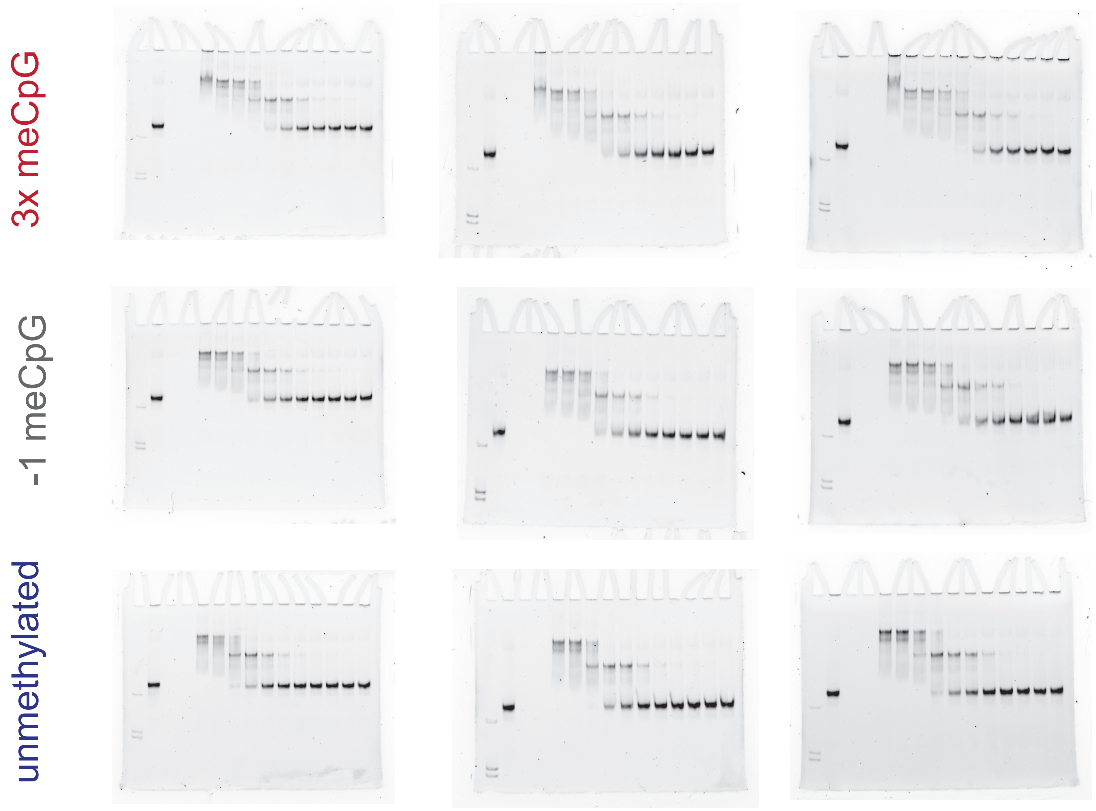

Figure 1E

MeCP2 on 15-N601-15

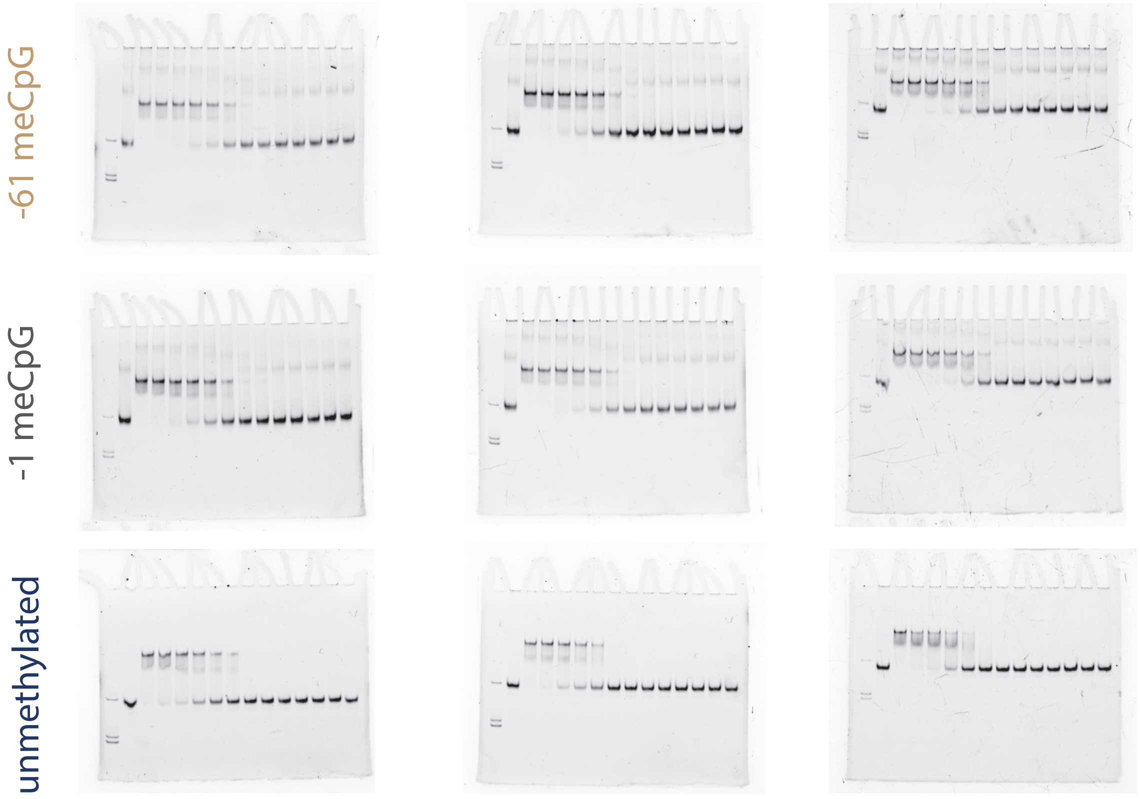

Figure 3C

MeCP2 on DNA and Nucleosome

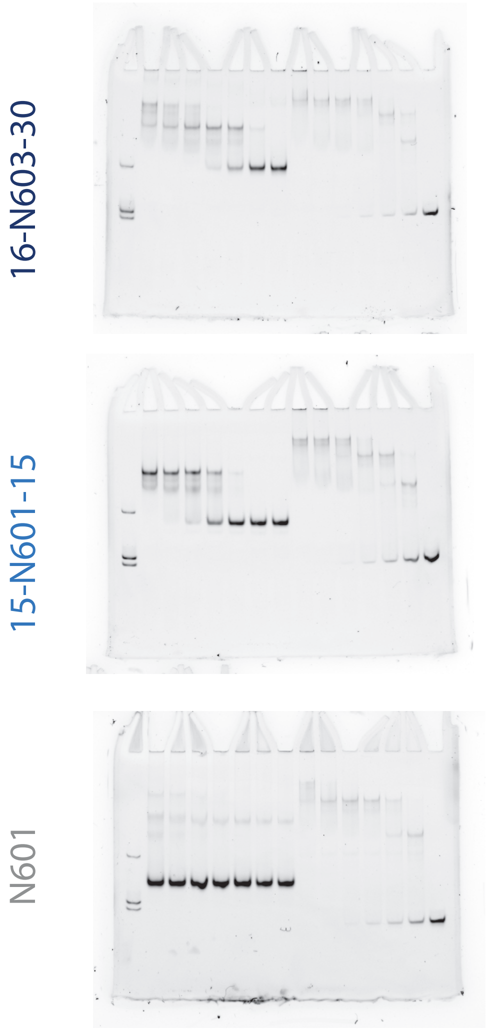

Figure 7A

MeCP2 on 15-N601-15 nucleosome and chromatosome

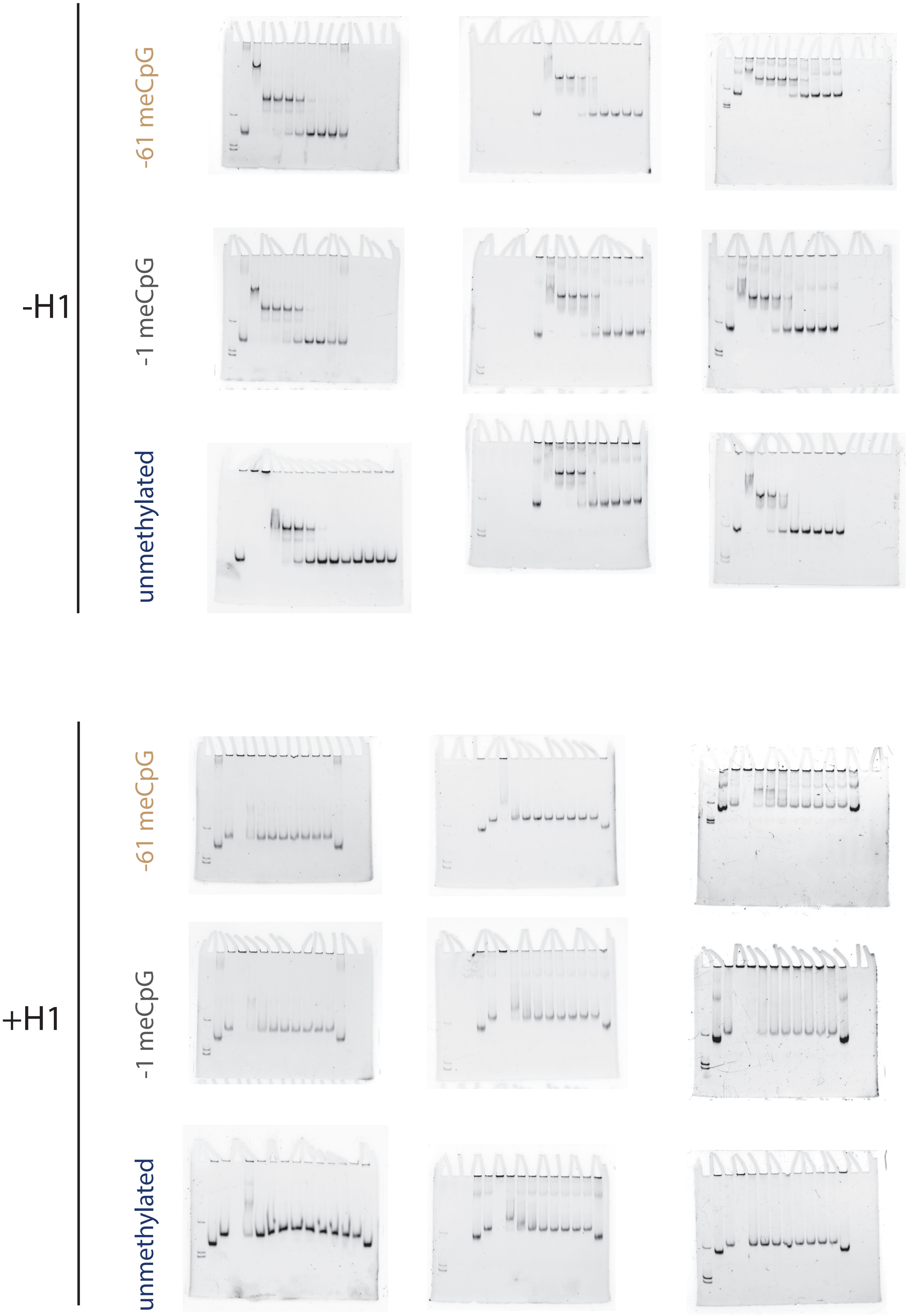

Supplement: Supplementary file 5 — Source Data File 2 [file 41467_2026_71741_MOESM5_ESM.pdf]
