# Supplementary material for: MeCP2 requires interactions with nucleosome linker DNA to read chromatin DNA methylation
Source: Nat Commun. 2026 Apr 17;17:5374. doi: 10.1038/s41467-026-71741-0 (PMC13276077; doi:10.1038/s41467-026-71741-0)

Supplementary Figure 1A

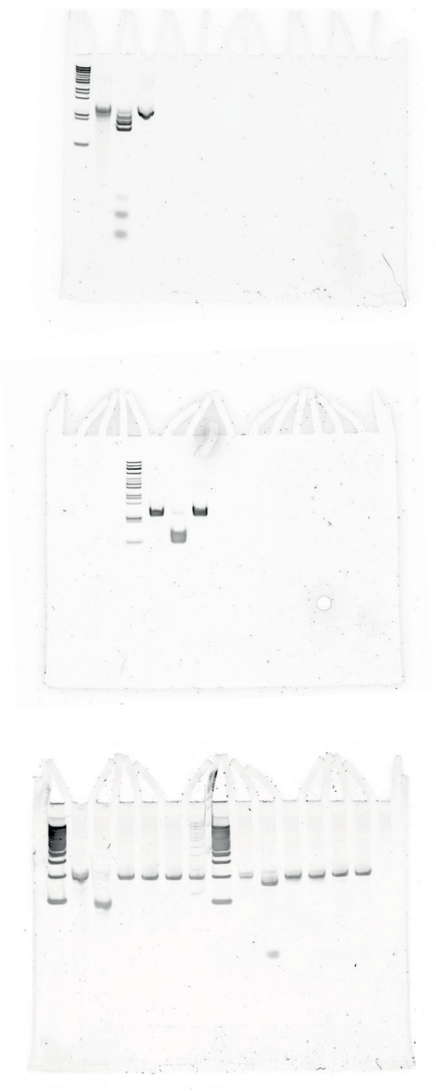

Supplementary Figure 1B

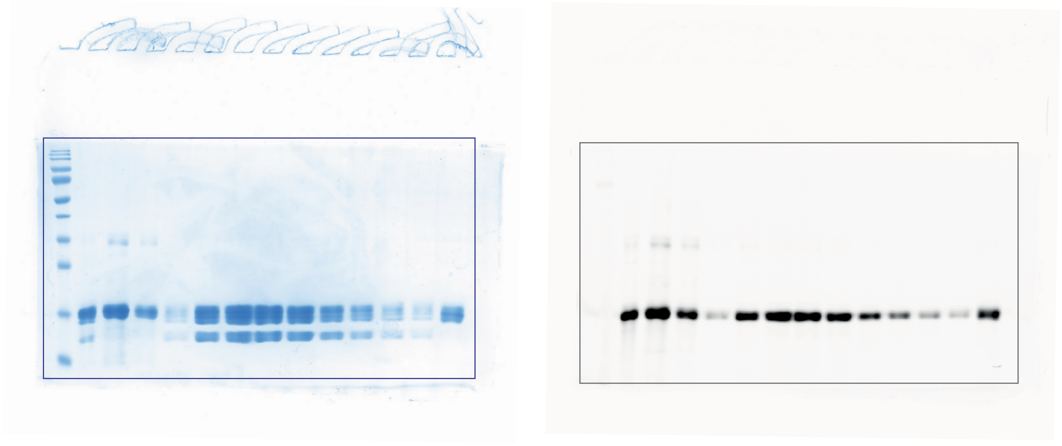

Supplementary Figure 1C

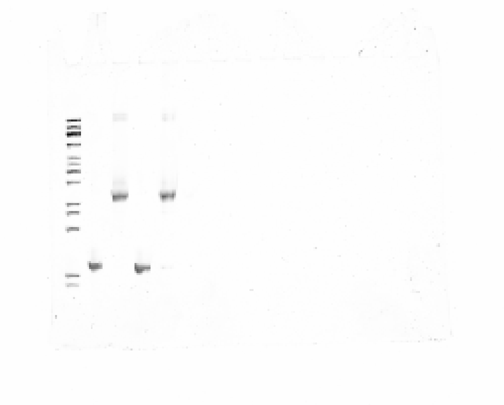

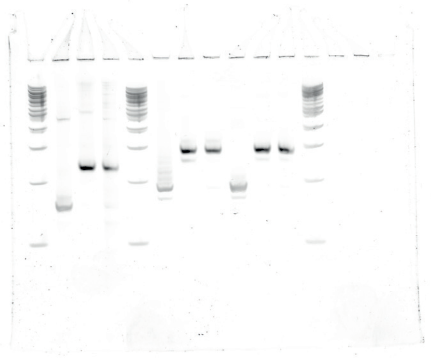

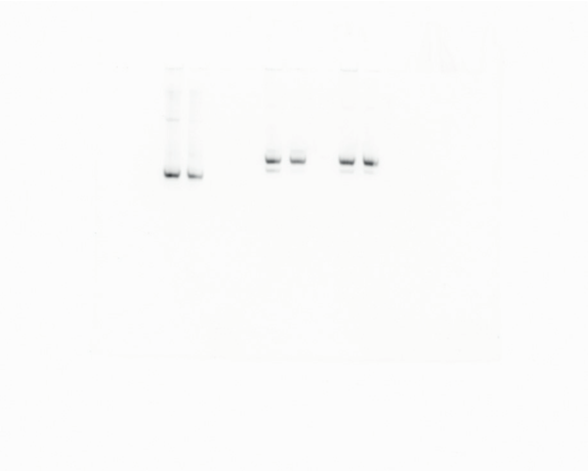

Supplementary Figure 2A

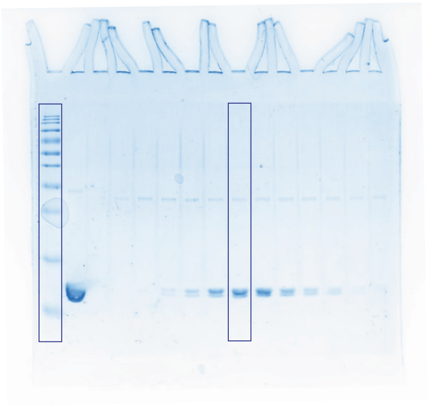

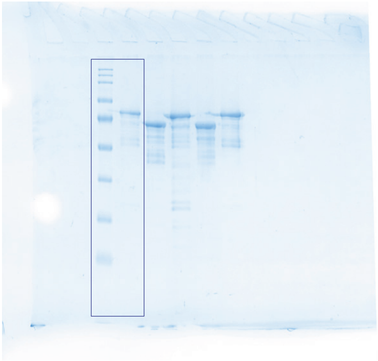

Supplementary Figure 2B

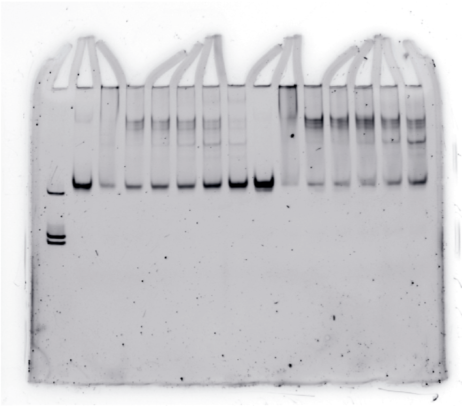

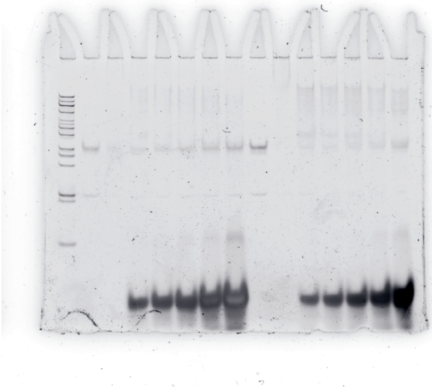

Supplementary Figure 2E

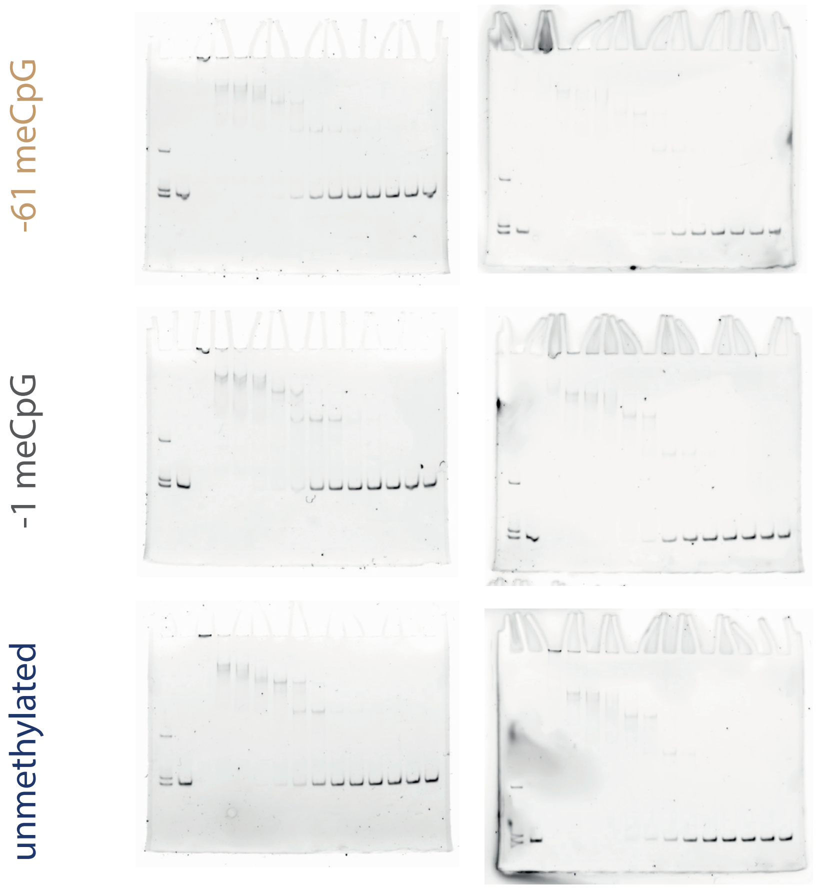

Supplementary Figure 4B

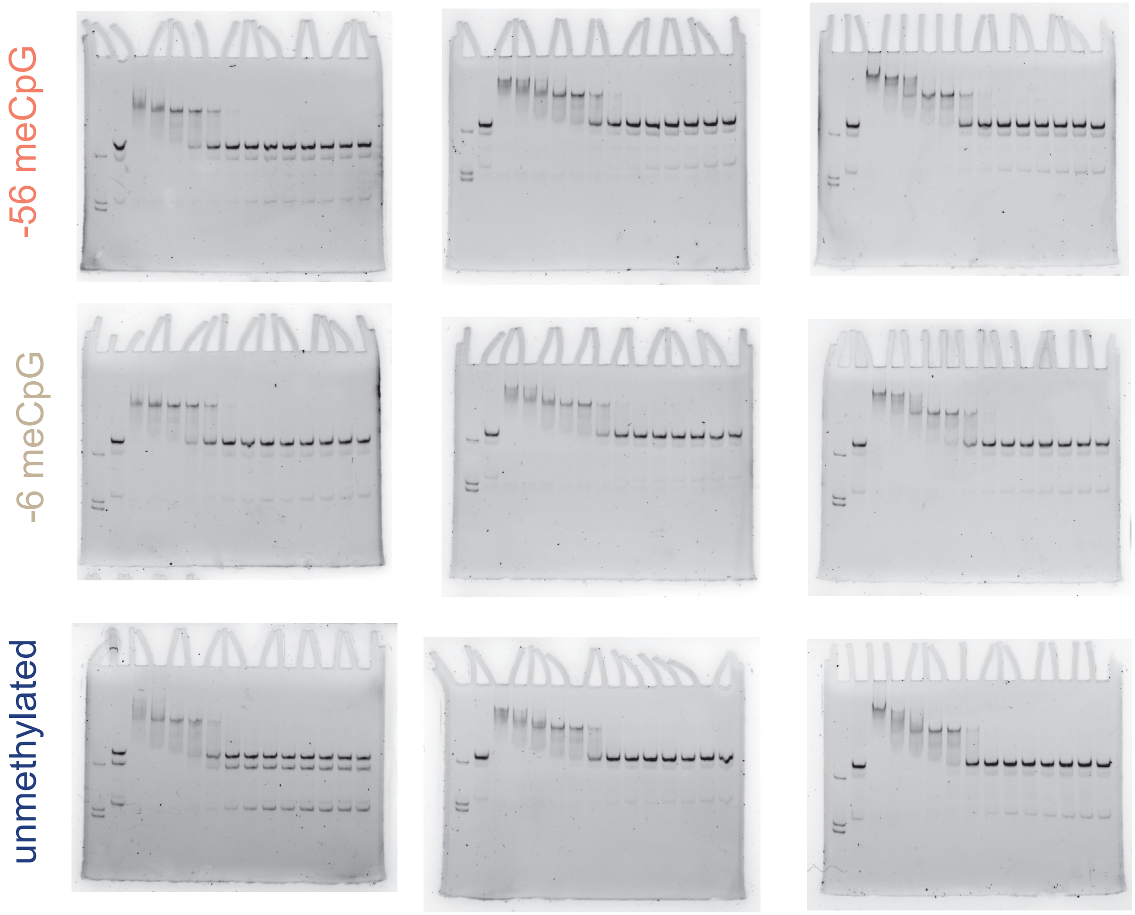

Supplementary Figure 4C

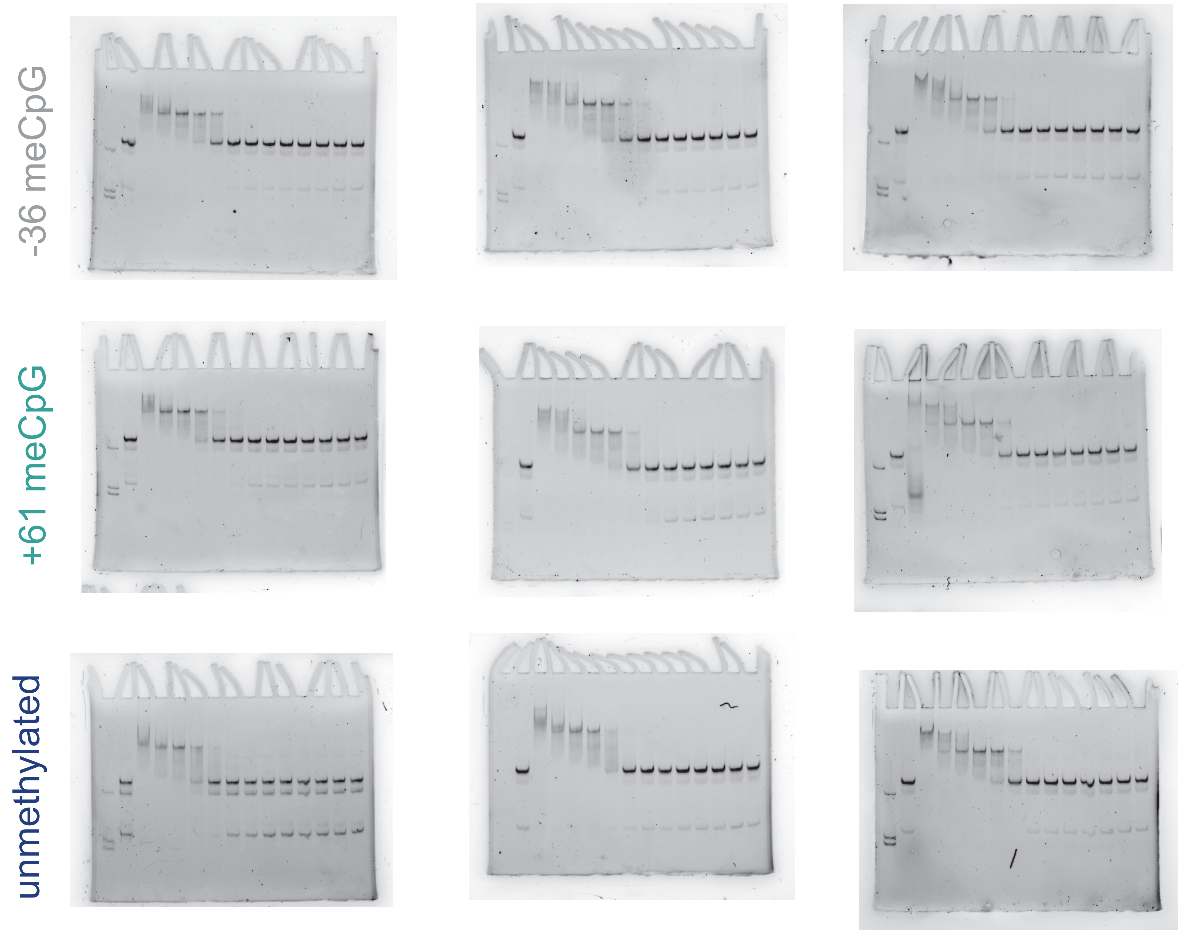

Supplementary Figure 5A

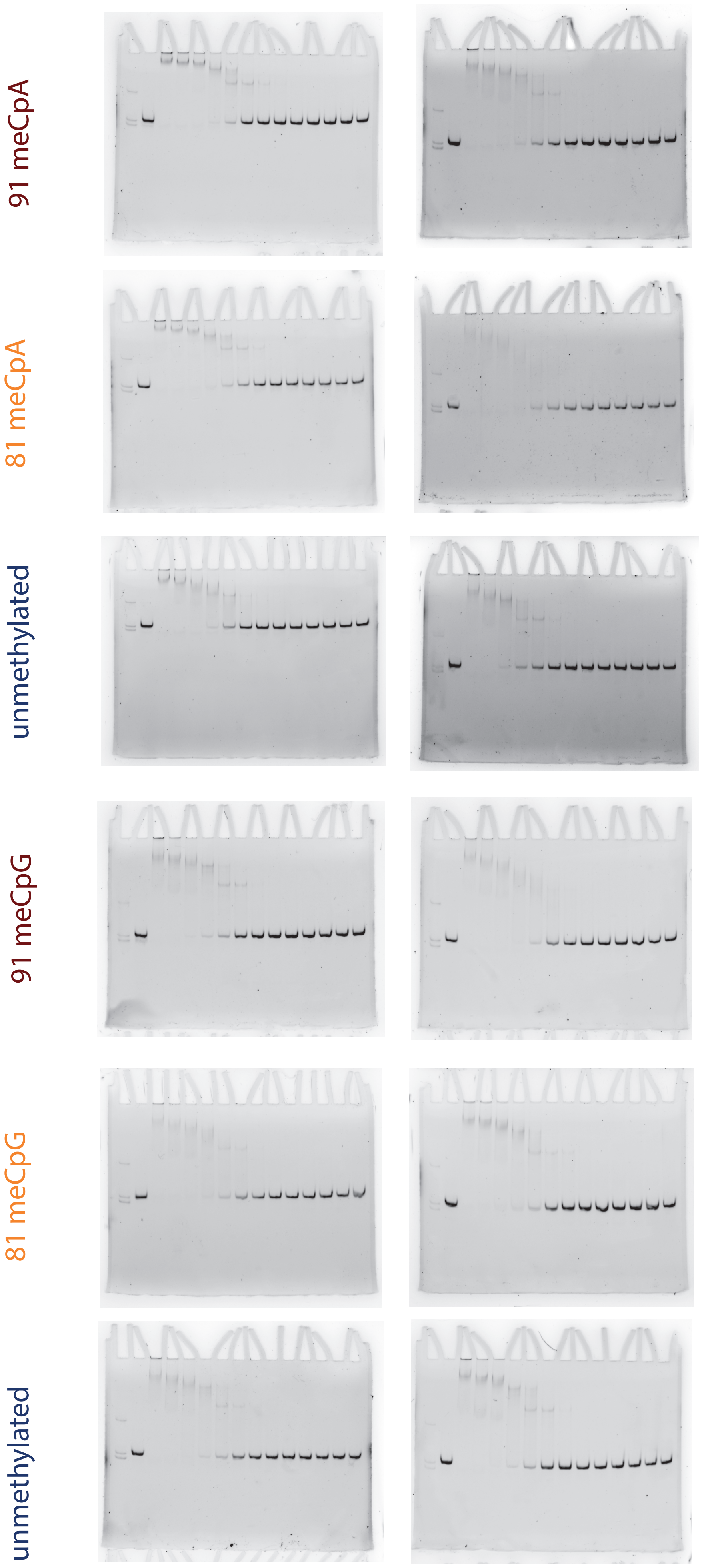

Supplementary Figure 5B

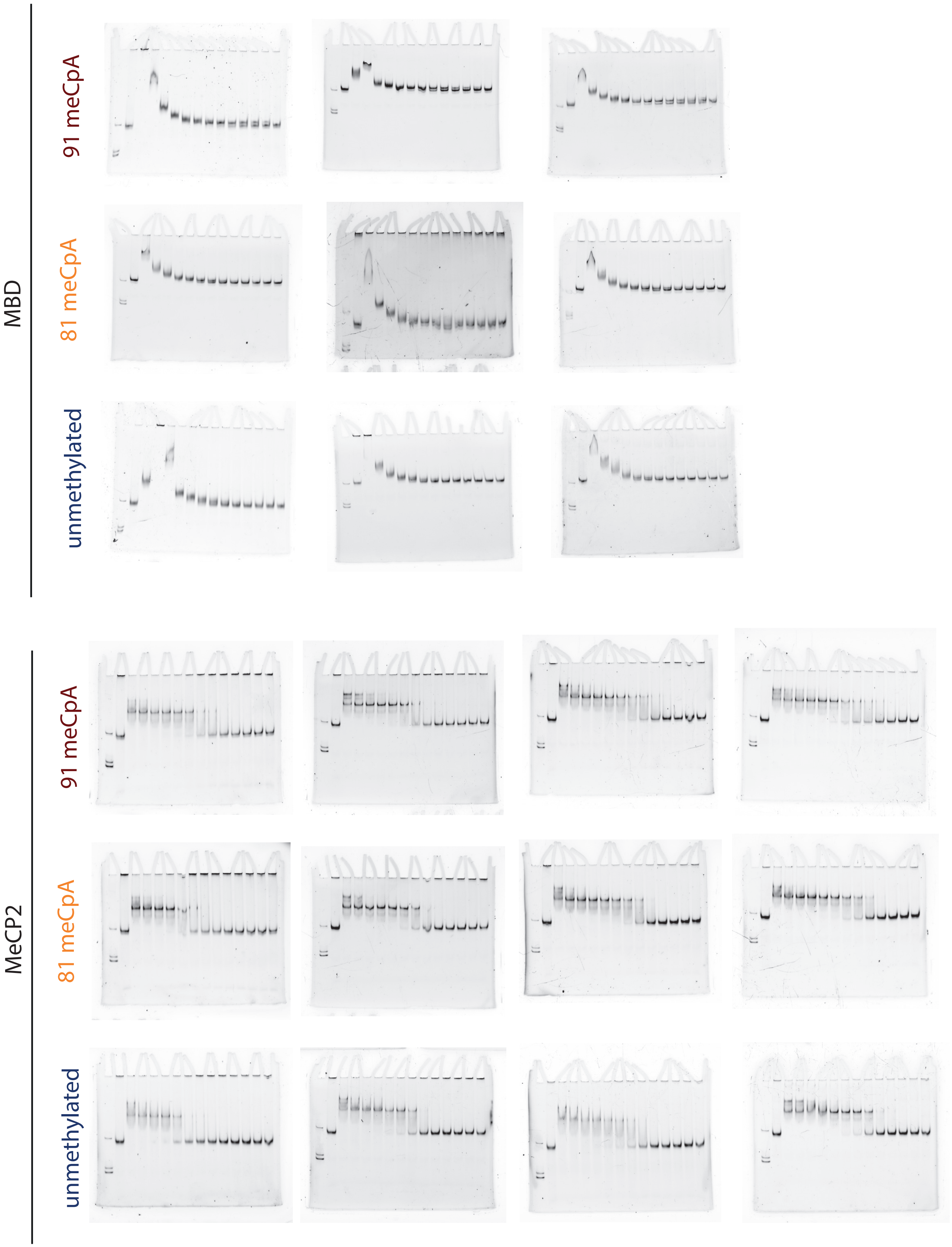

Supplementary Figure 5C

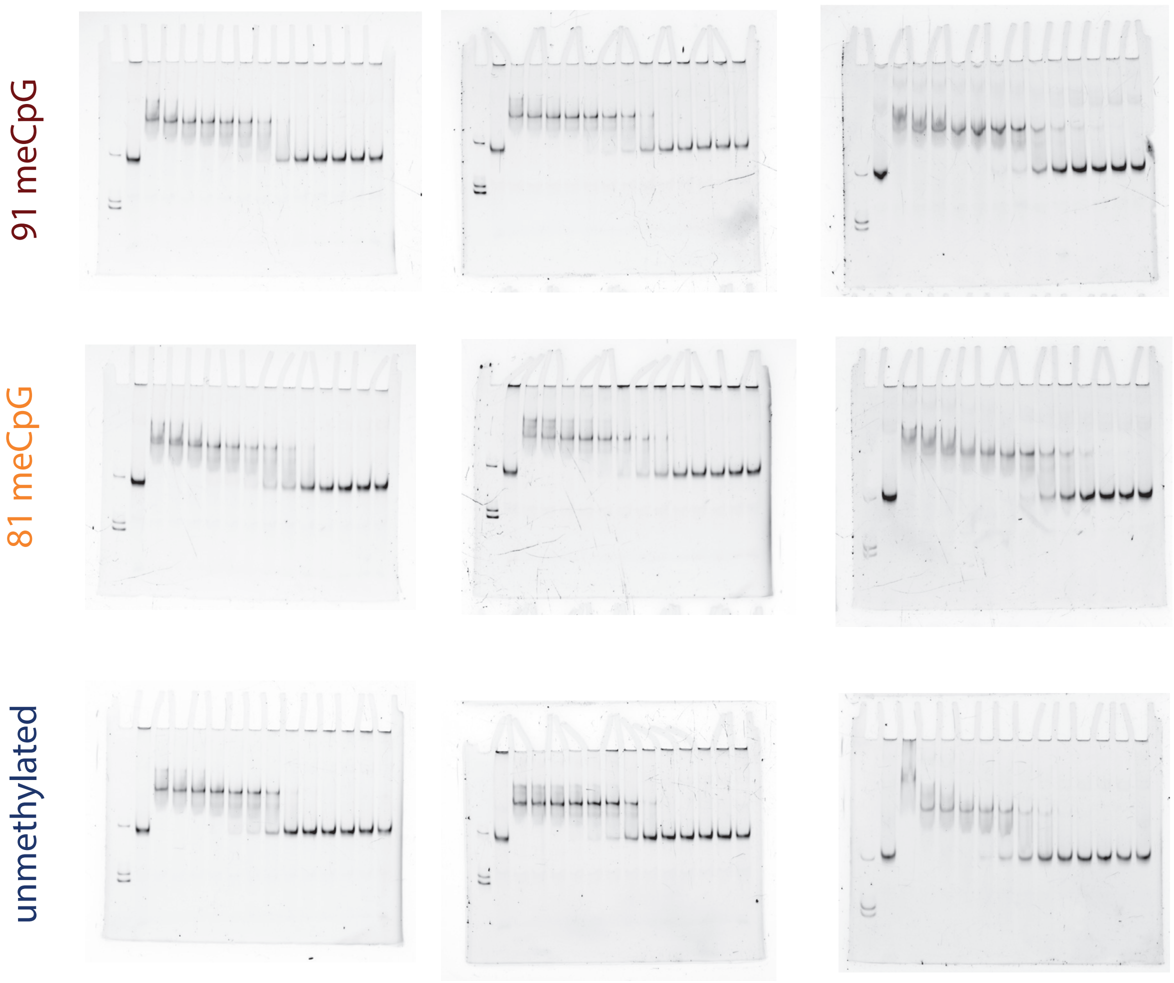

Supplementary Figure 6B

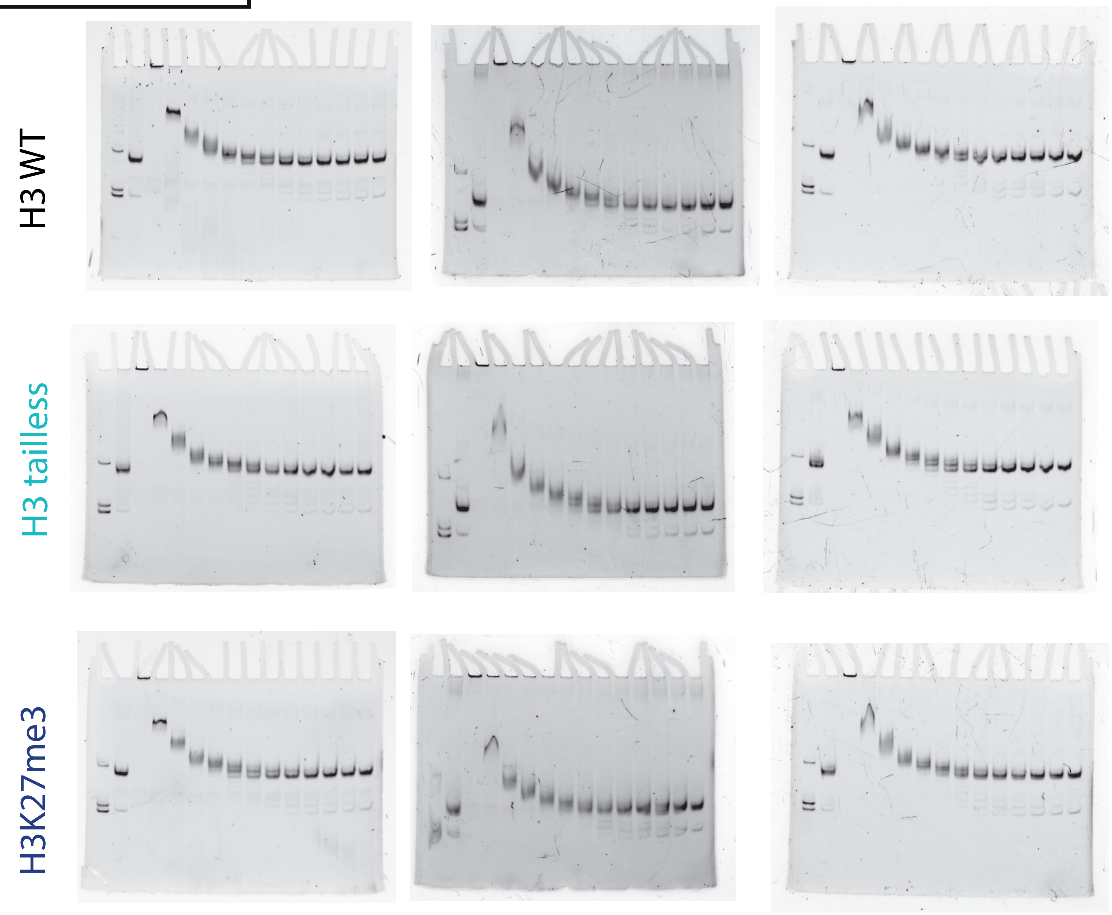

Supplementary Figure 6C

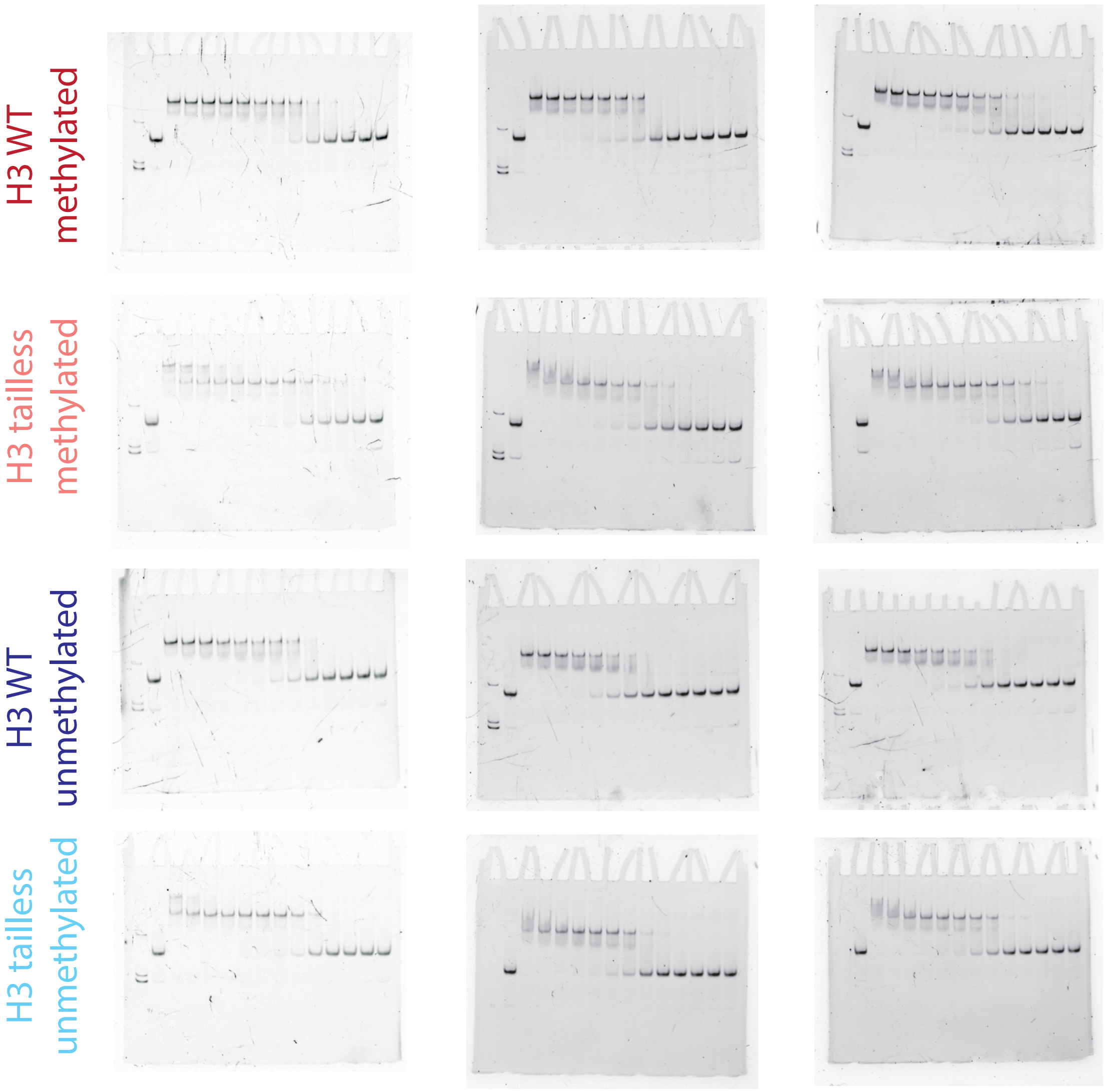

Figure 7A

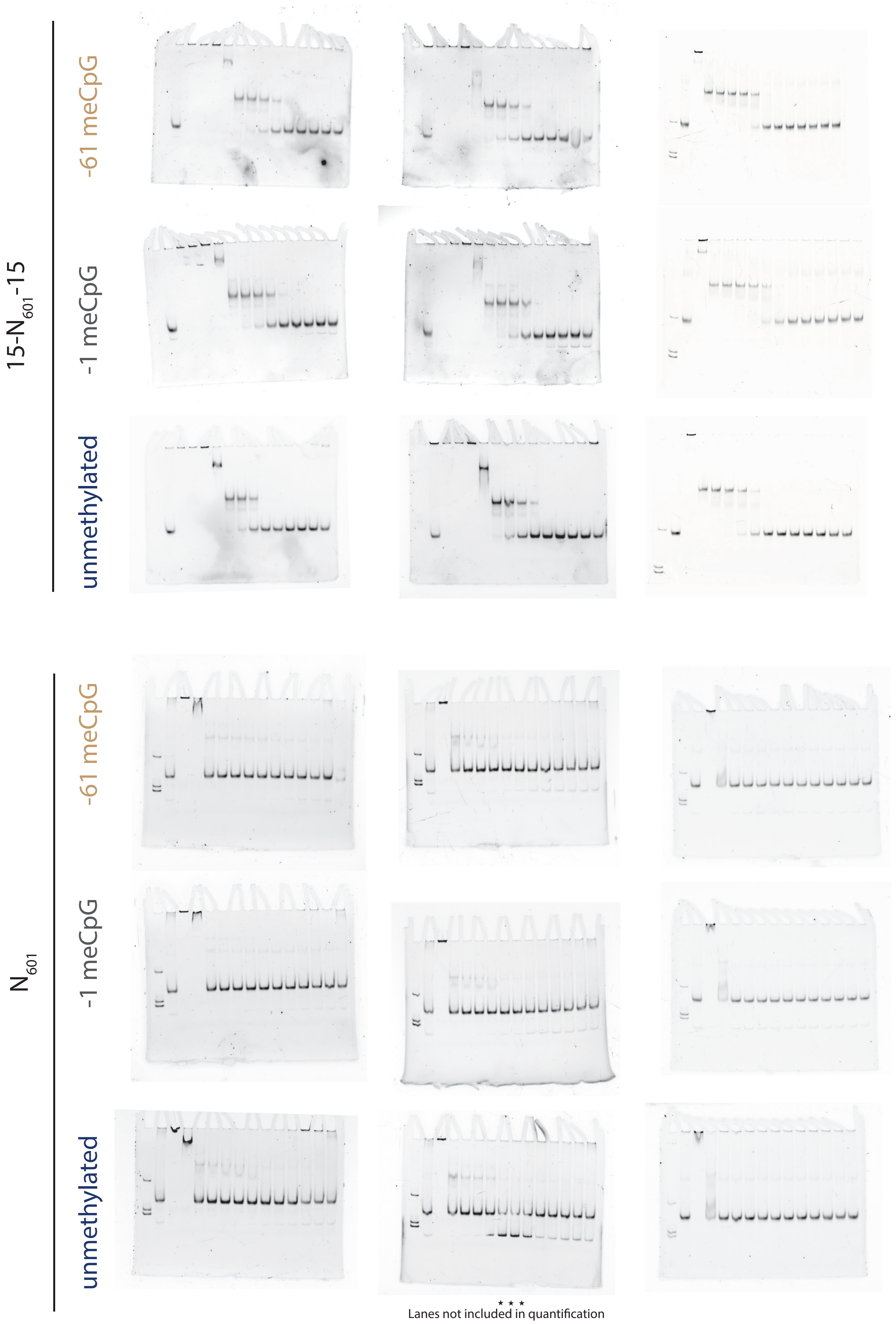
15-N_{601}-15
-61 meCpG
-1 meCpG
unmethylated
N_{601}
-61 meCpG
-1 meCpG
unmethylated
* * *
Lanes not included in quantification

Supplementary Figure 7B

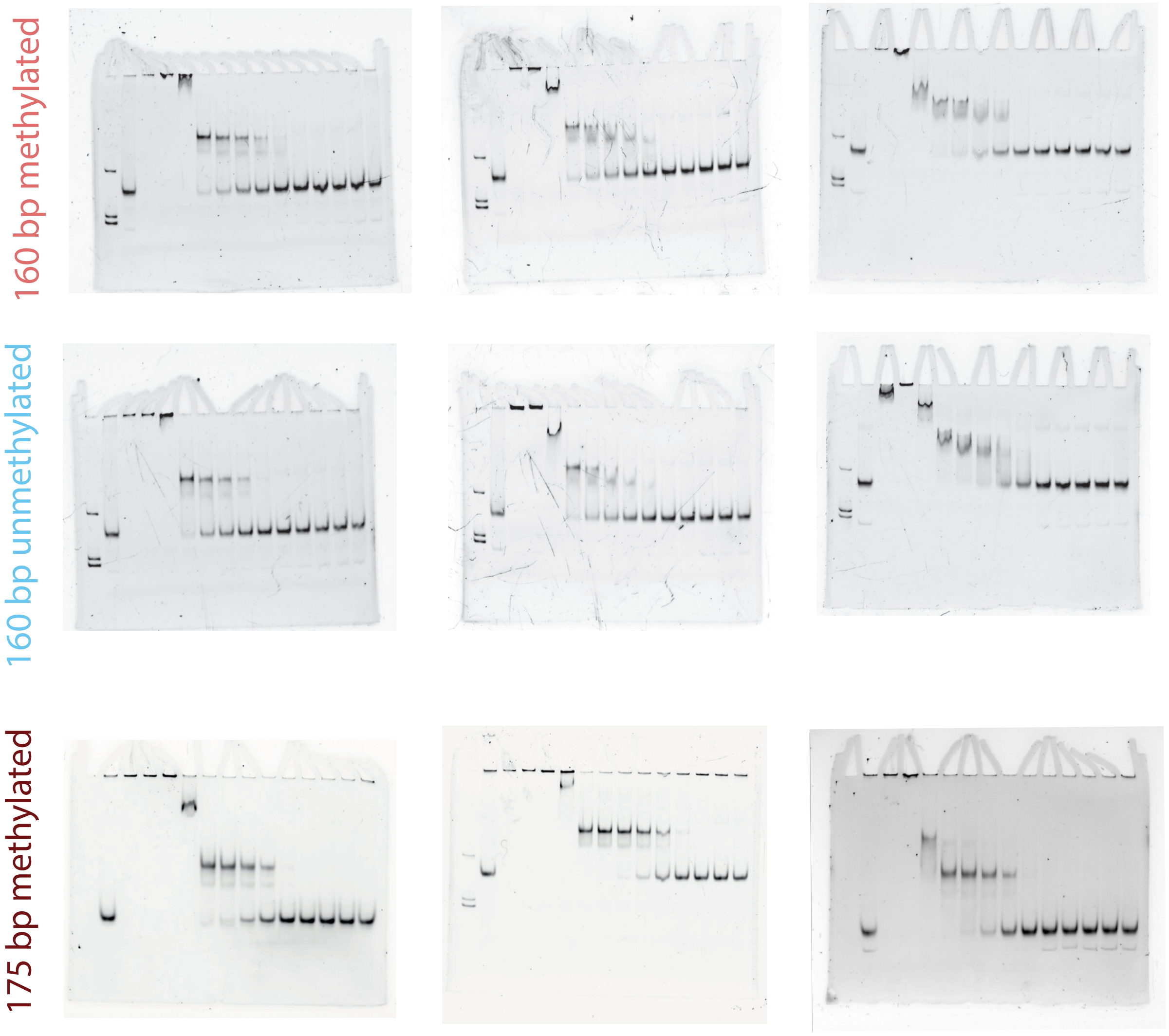

Supplementary Figure 7C

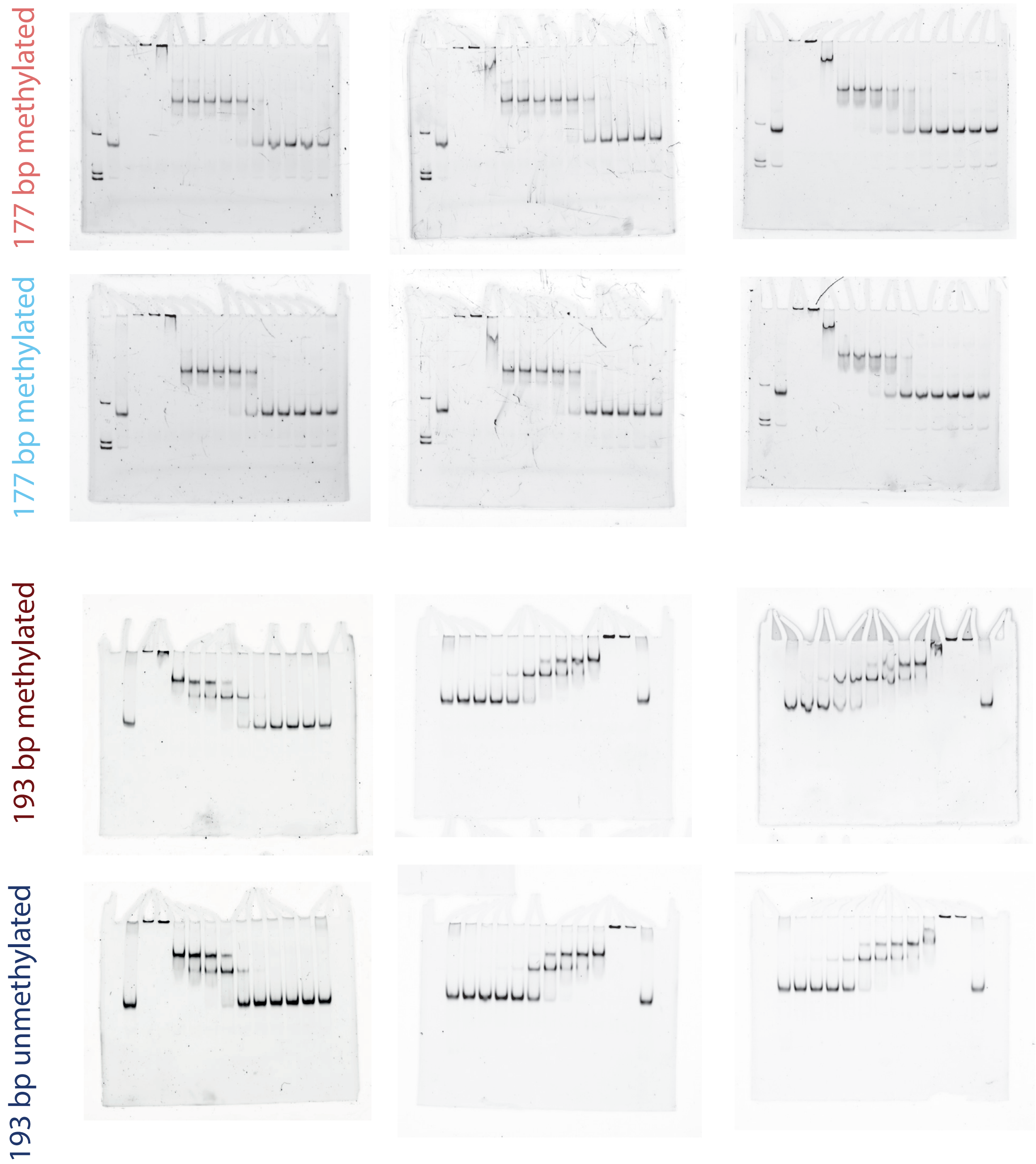

Supplementary Figure 8A

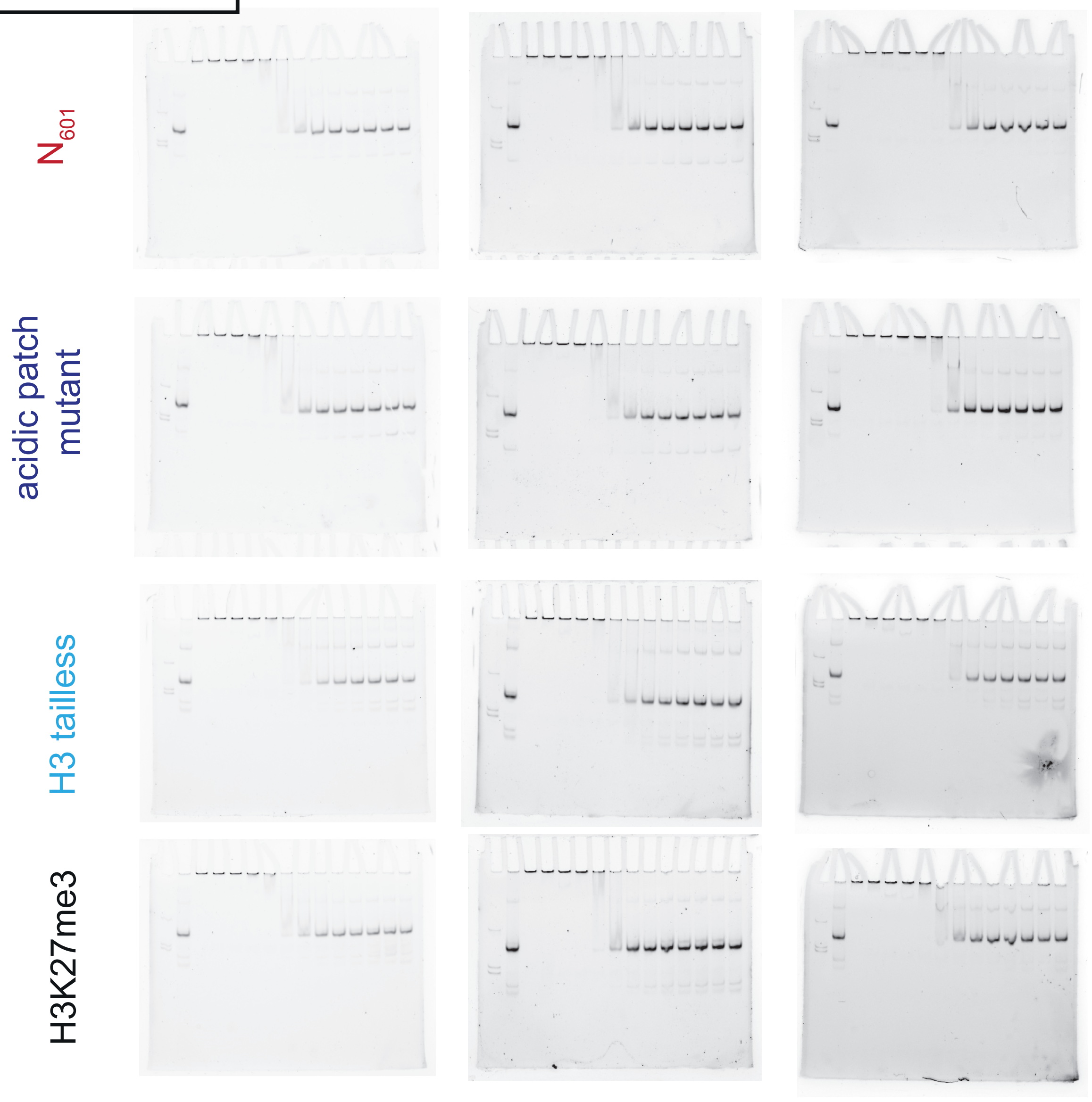

Supplementary Figure 8B

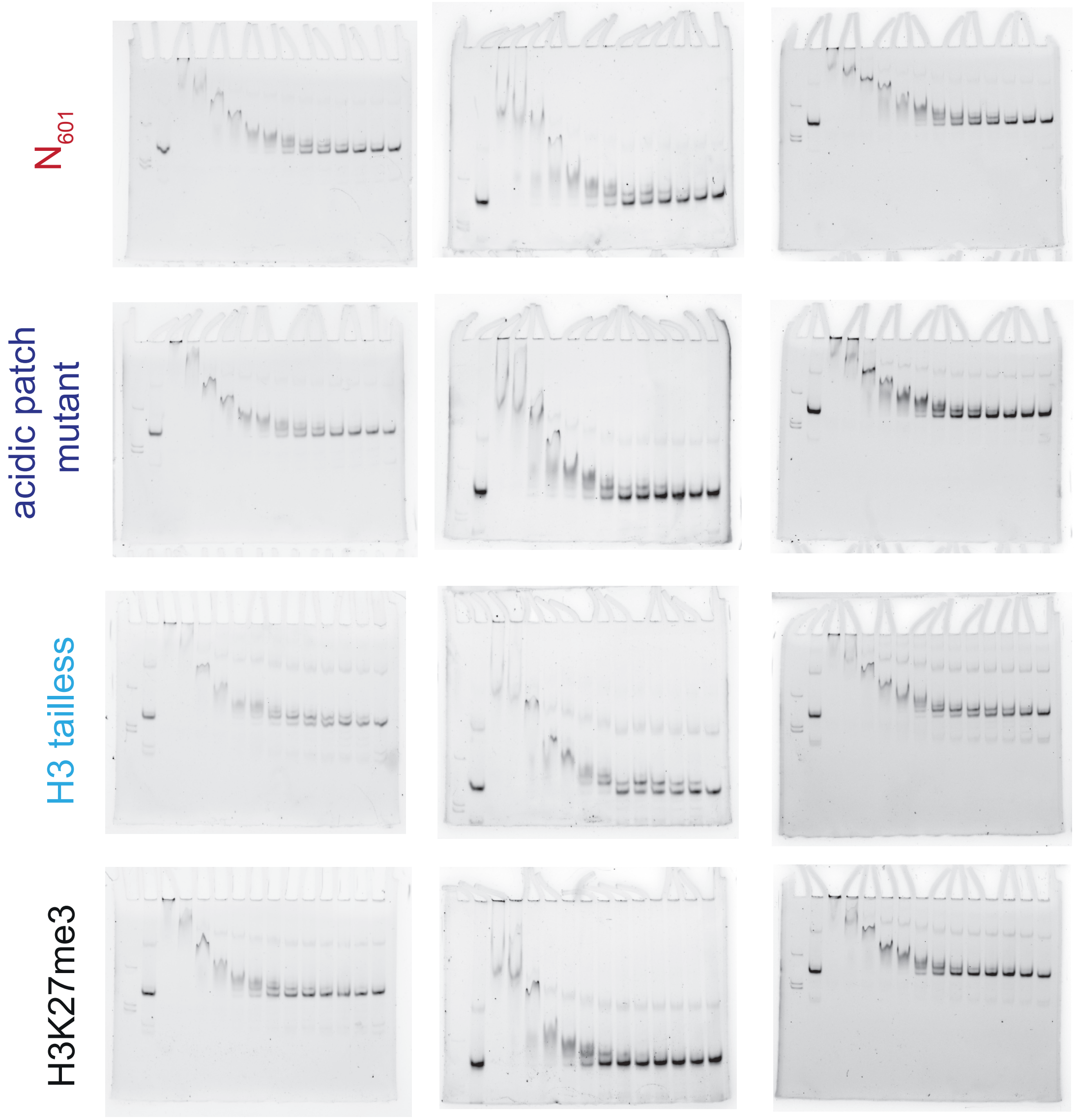

Supplementary Figure 8C

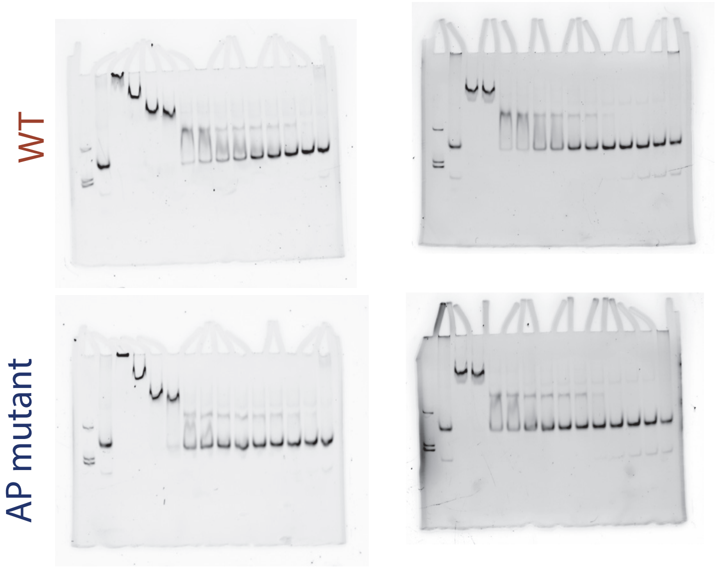

Supplementary Figure 8D

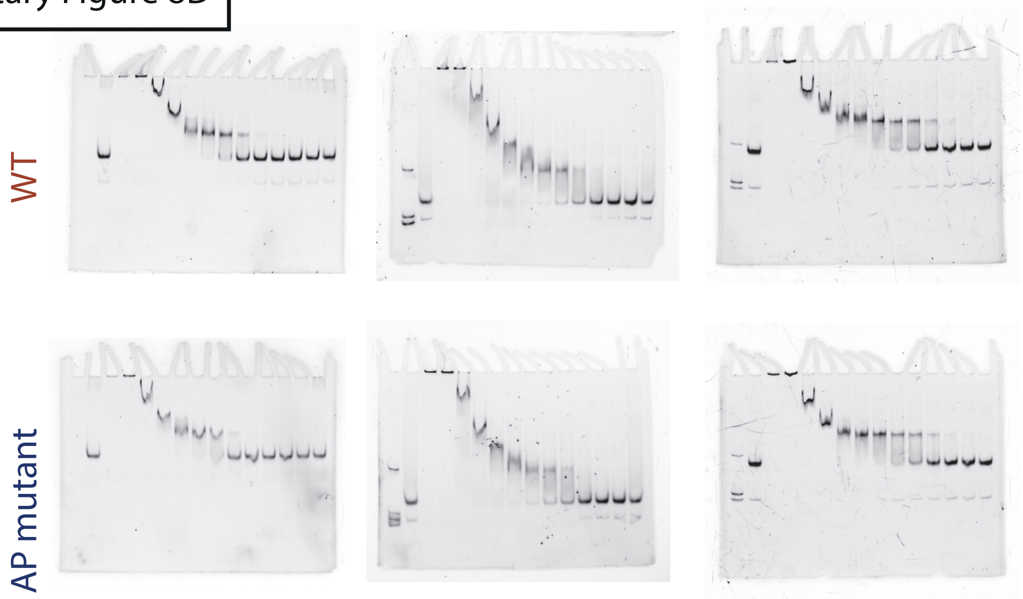

Supplementary Figure 8E

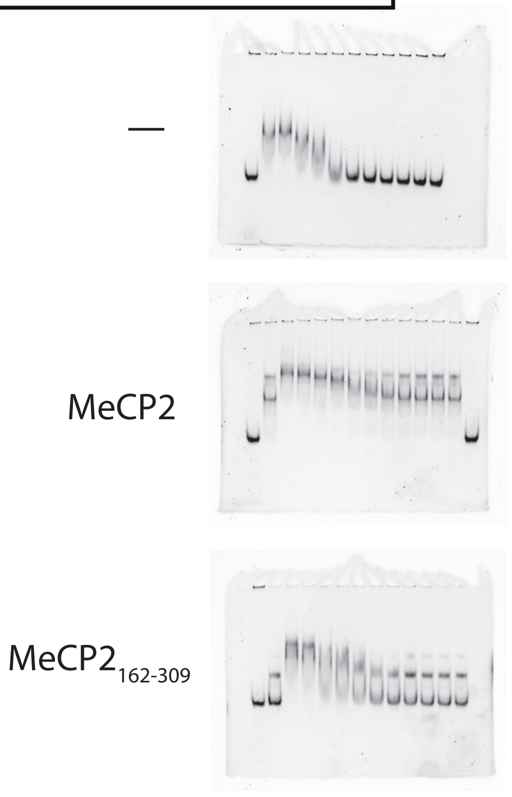

Supplementary Figure 9A

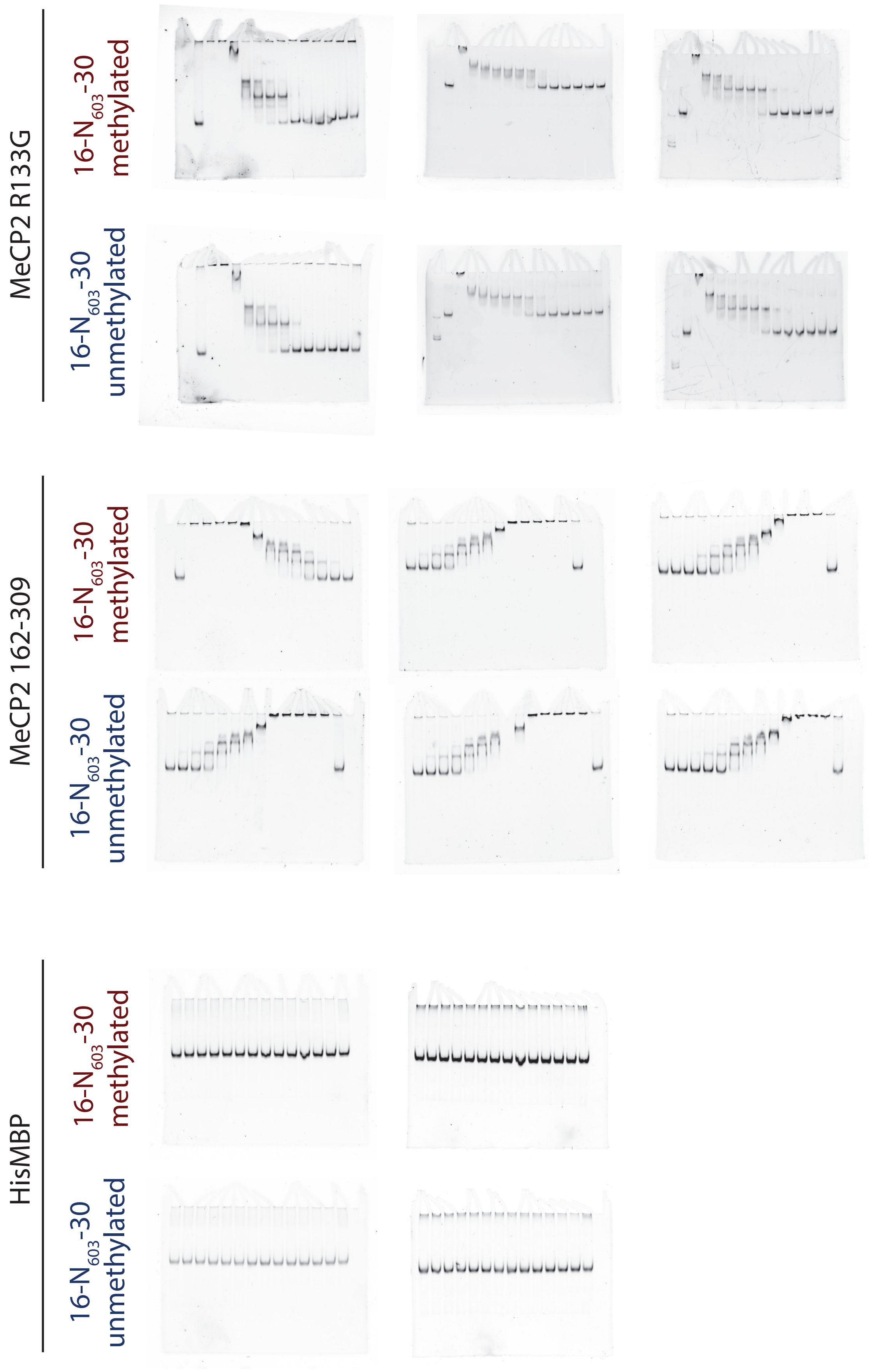

Supplementary Figure 9B

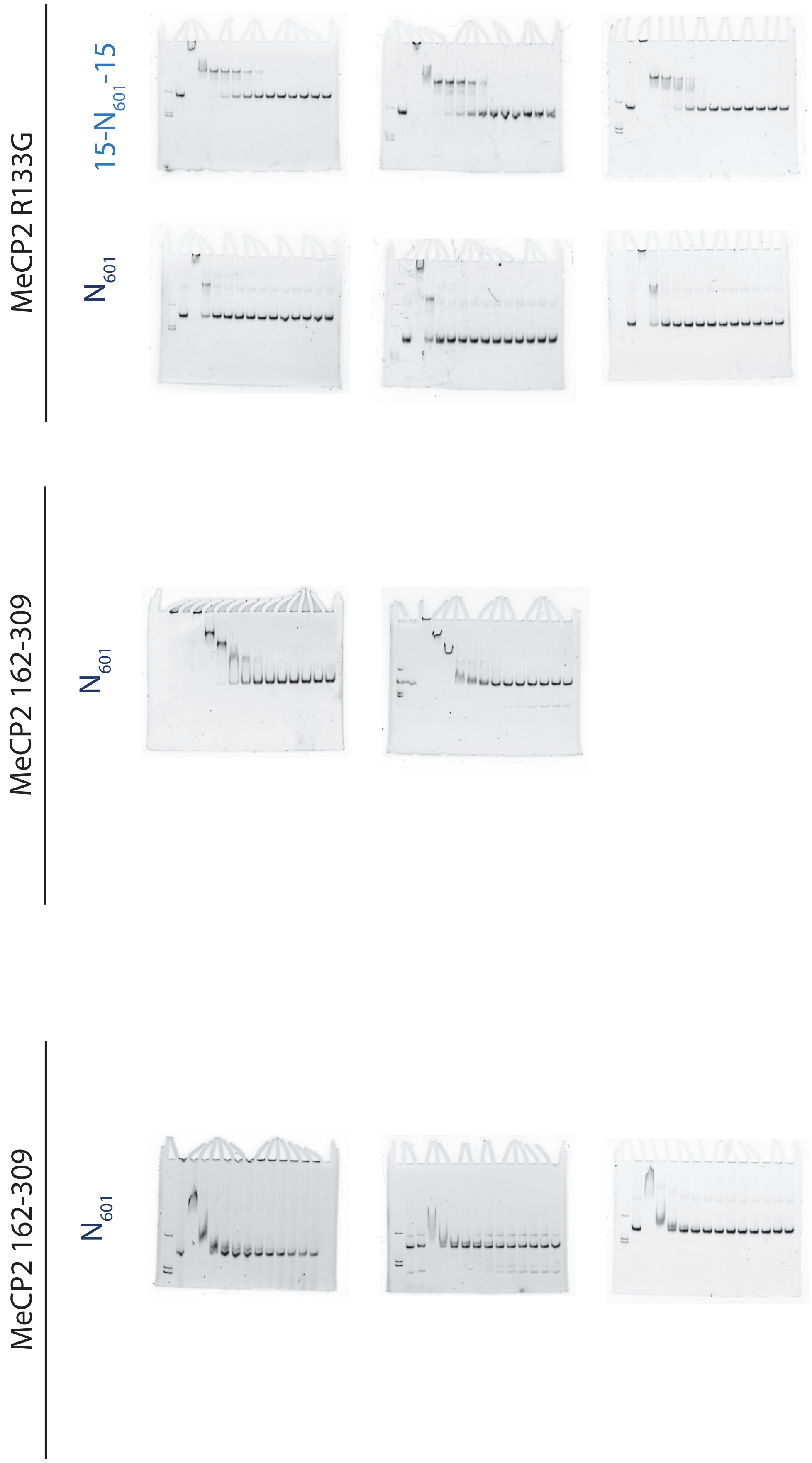

Supplementary Figure 9C

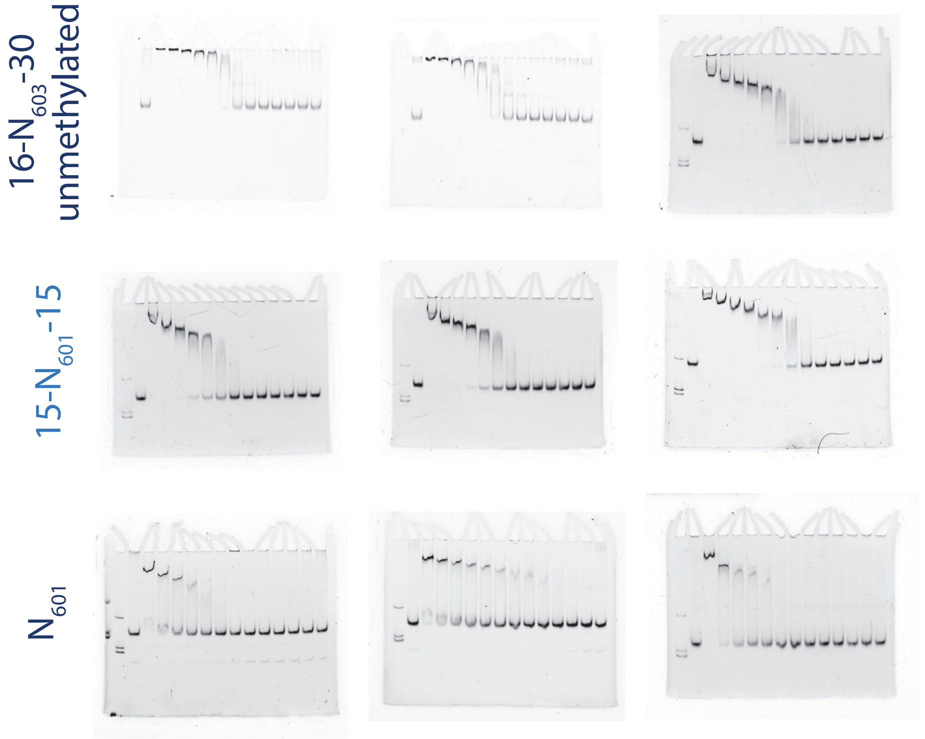

Supplementary Figure 9D

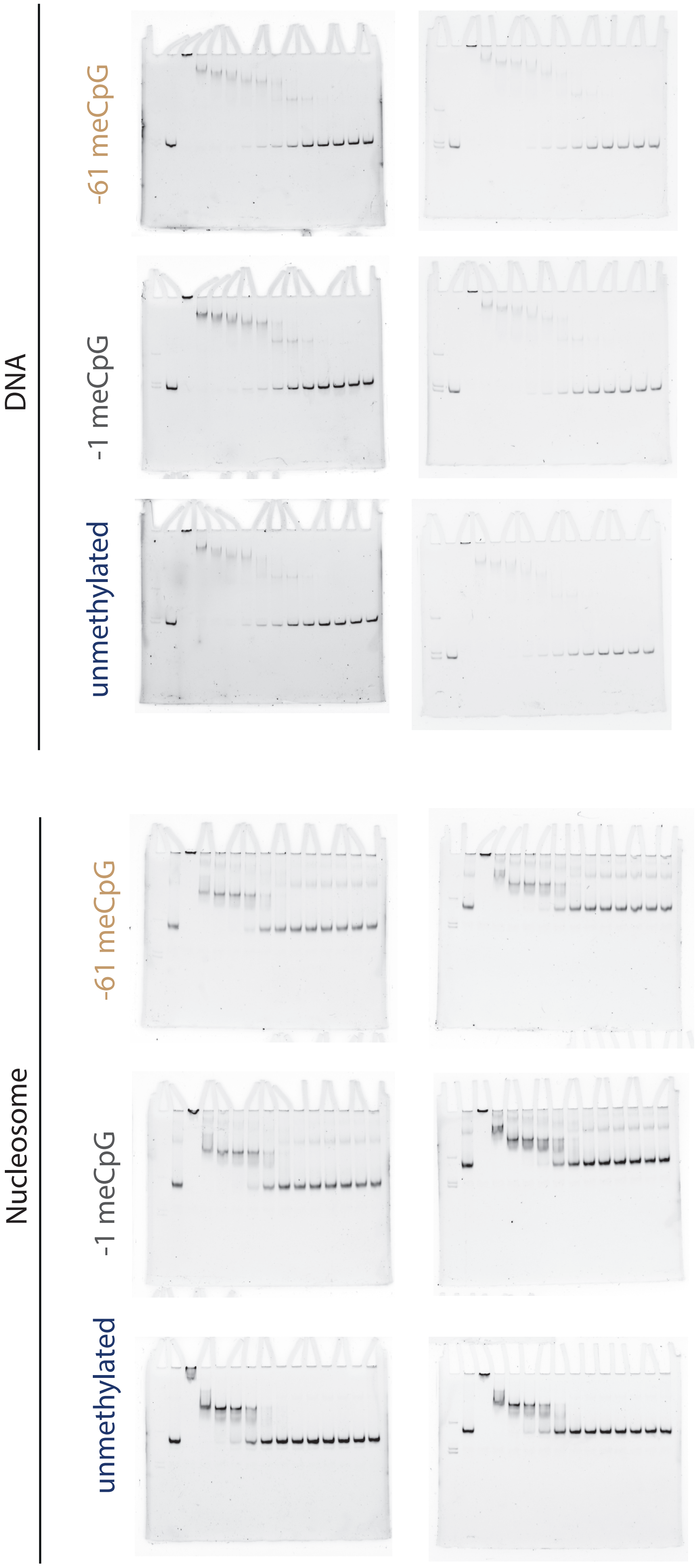

Supplementary Figure 10A

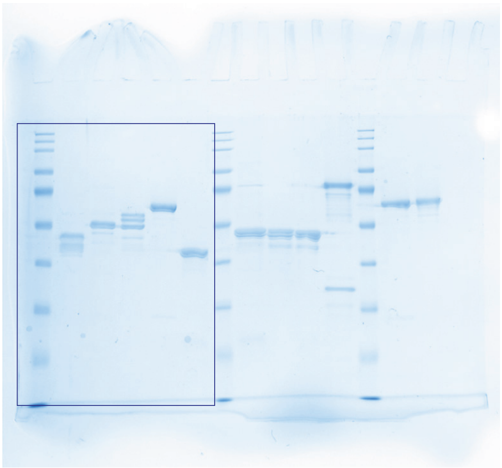

Supplementary Figure 10B

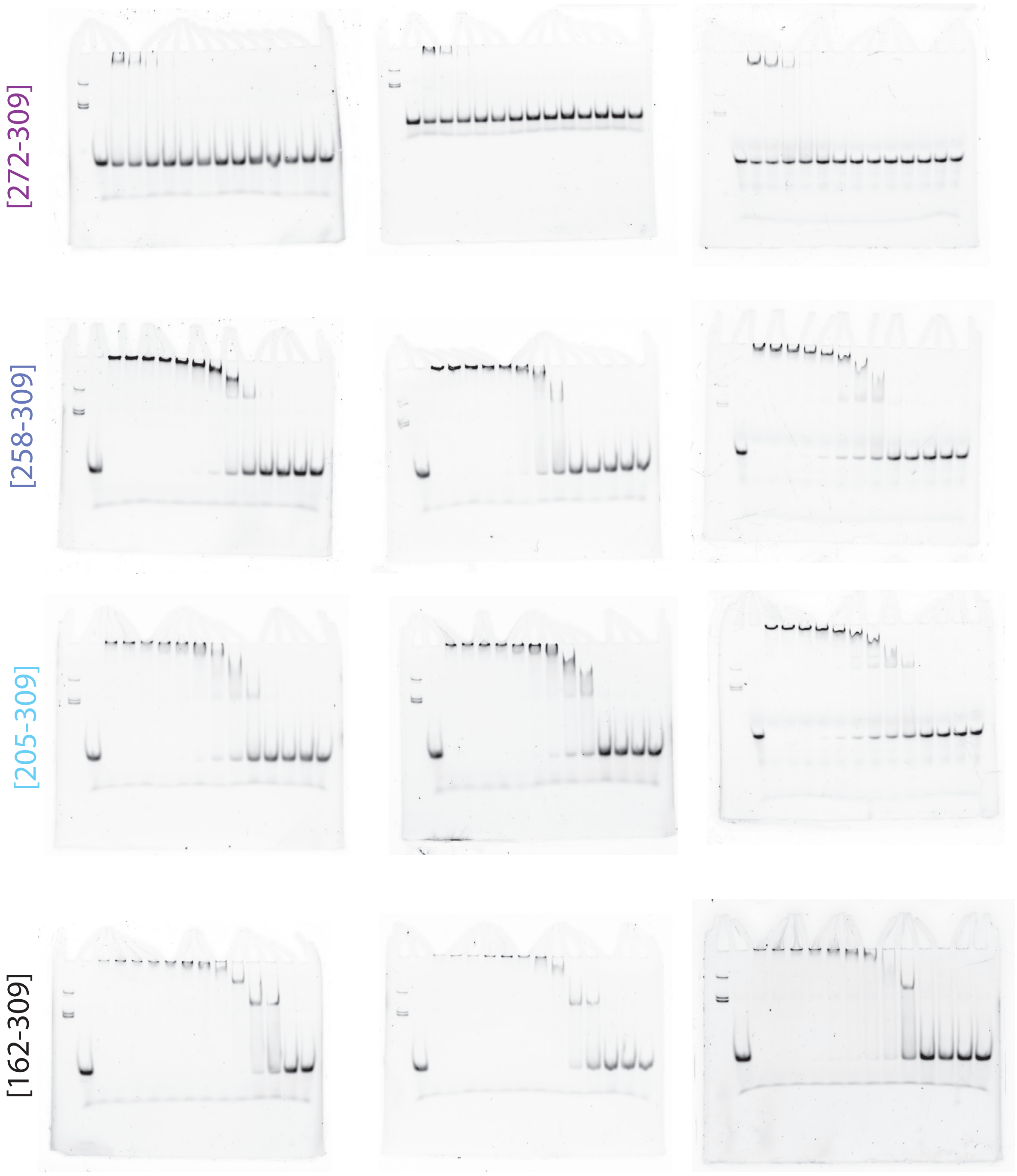

Supplementary Figure 10C

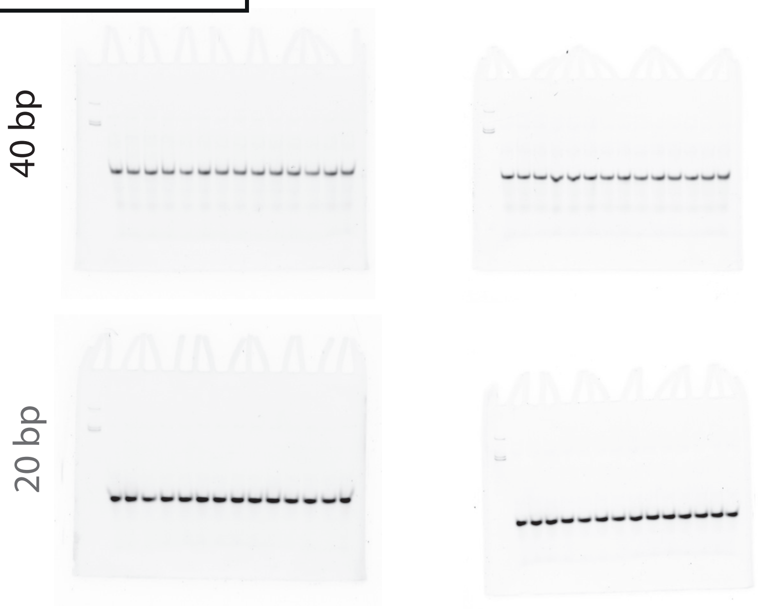

Supplementary Figure 10D

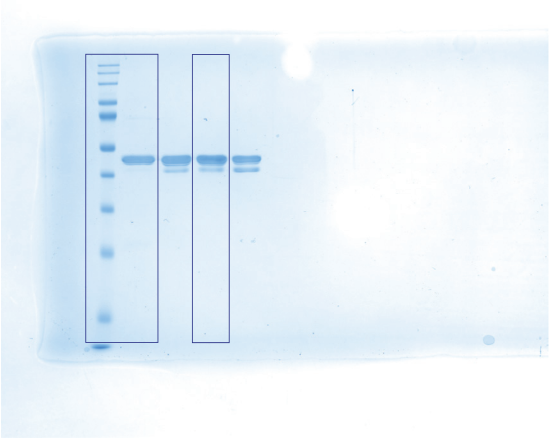

Supplementary Figure 10E

205-257

205-257 K254N/ K256N

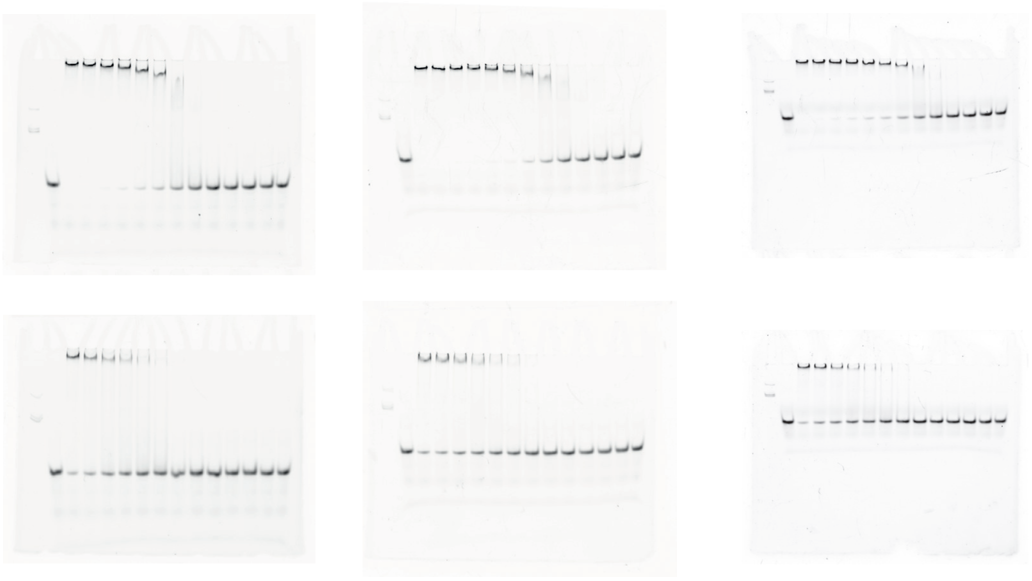

Supplementary Figure 10F

40 bp

20 bp

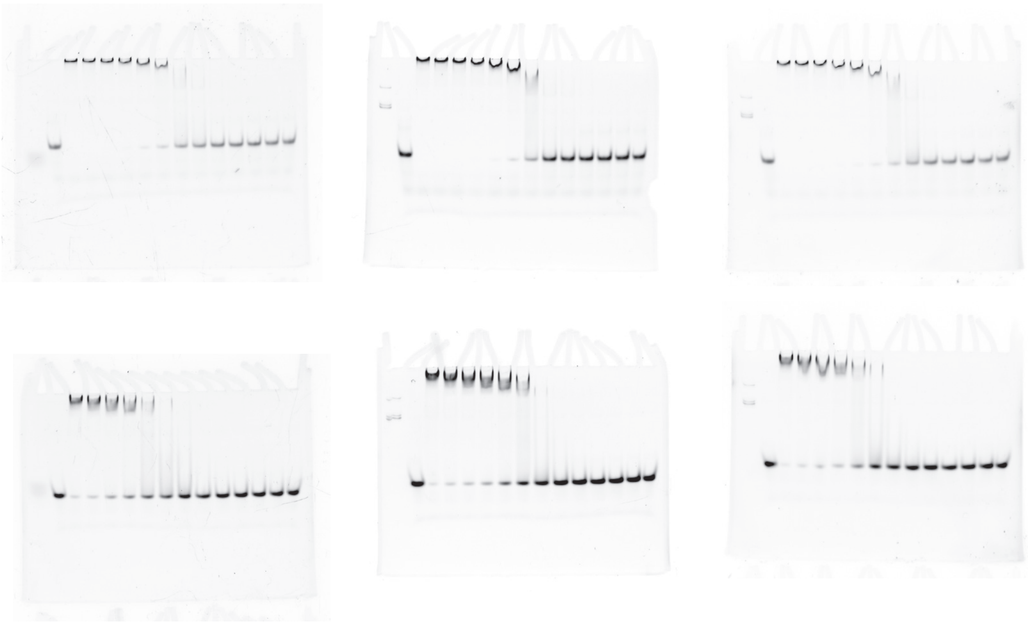

Supplementary Figure 11A

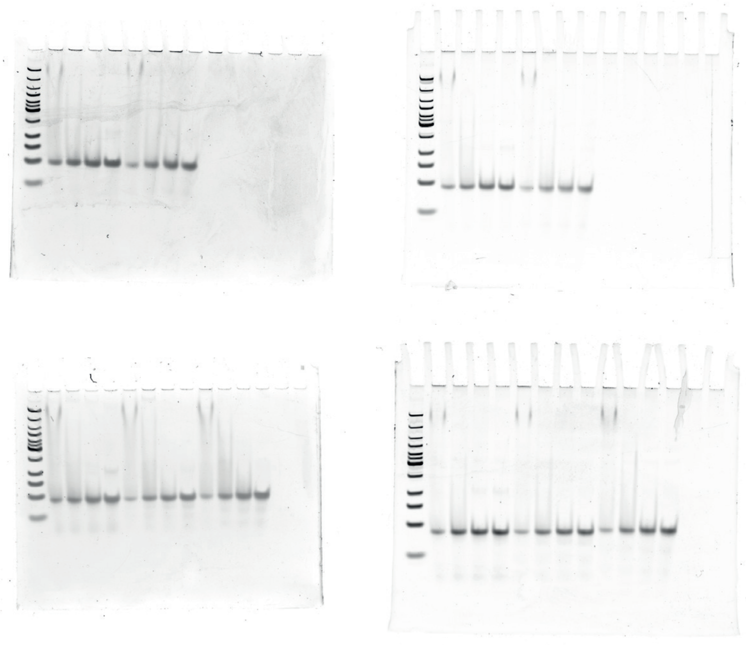

Supplementary Figure 11B

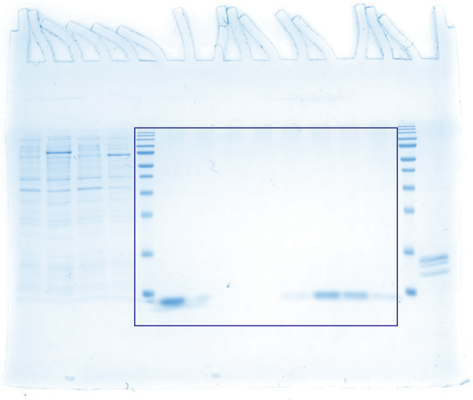

Supplementary Figure 11C

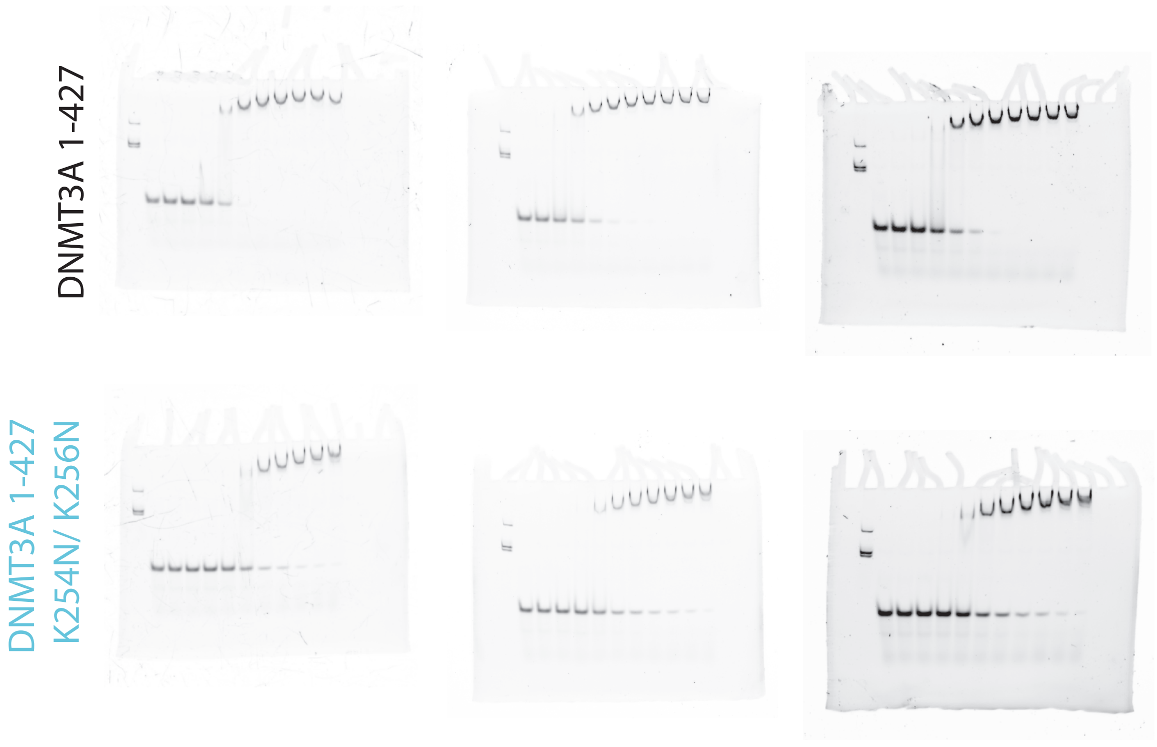

Supplementary Figure 12A

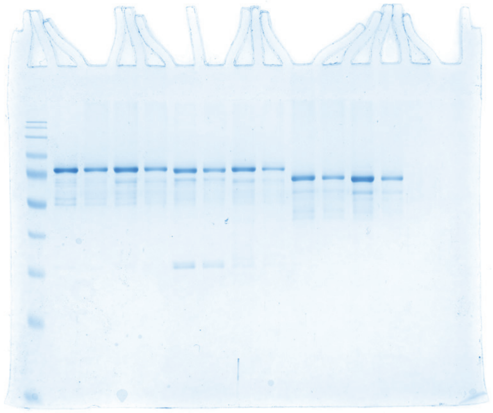

Supplementary Figure 12B

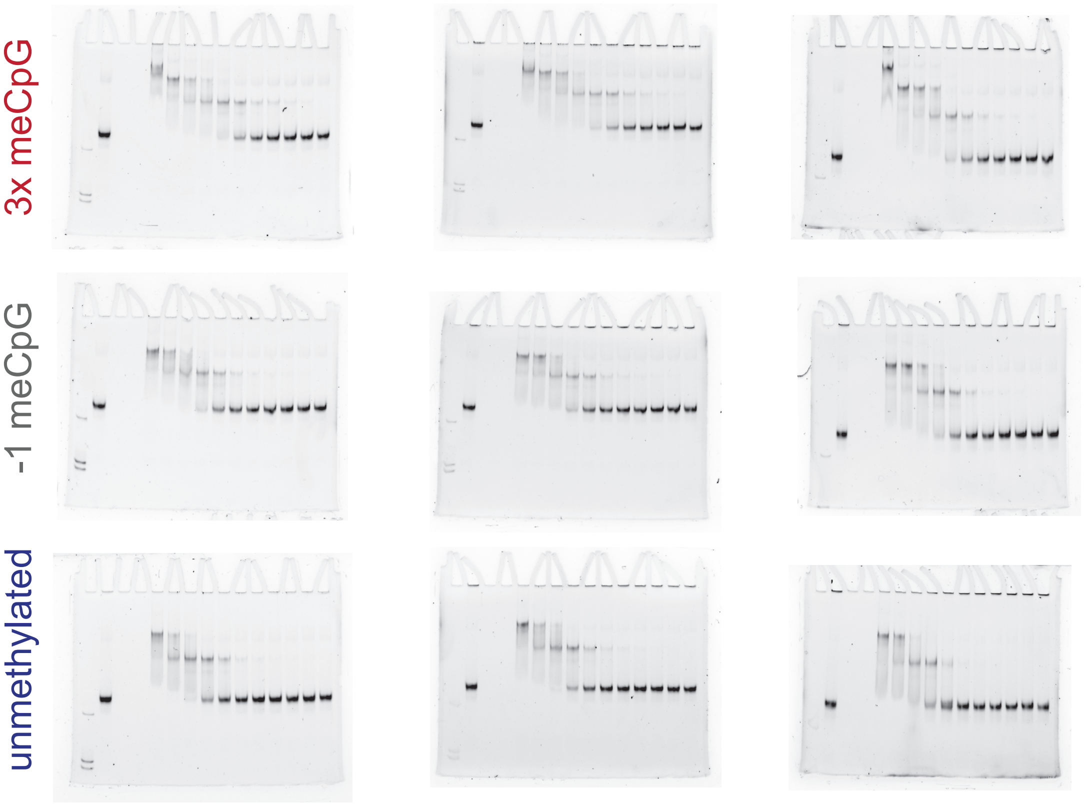

Supplementary Figure 13B

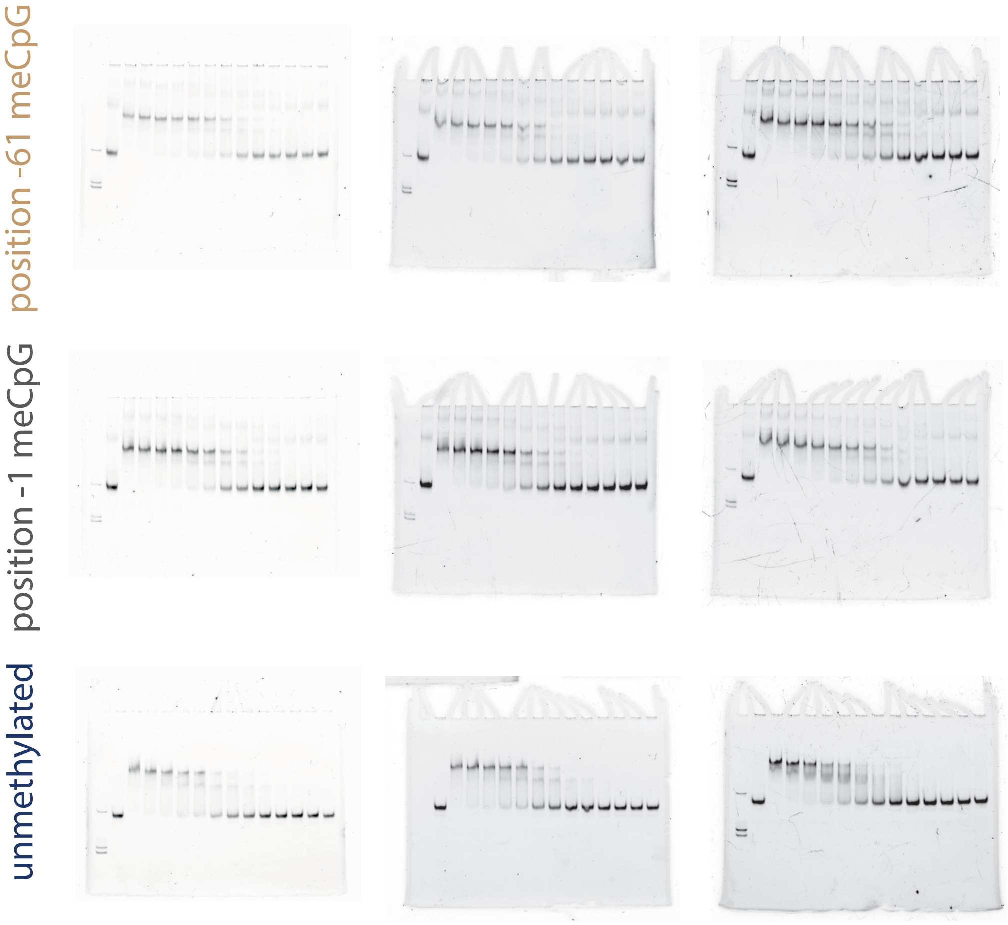

Supplementary Figure 13B

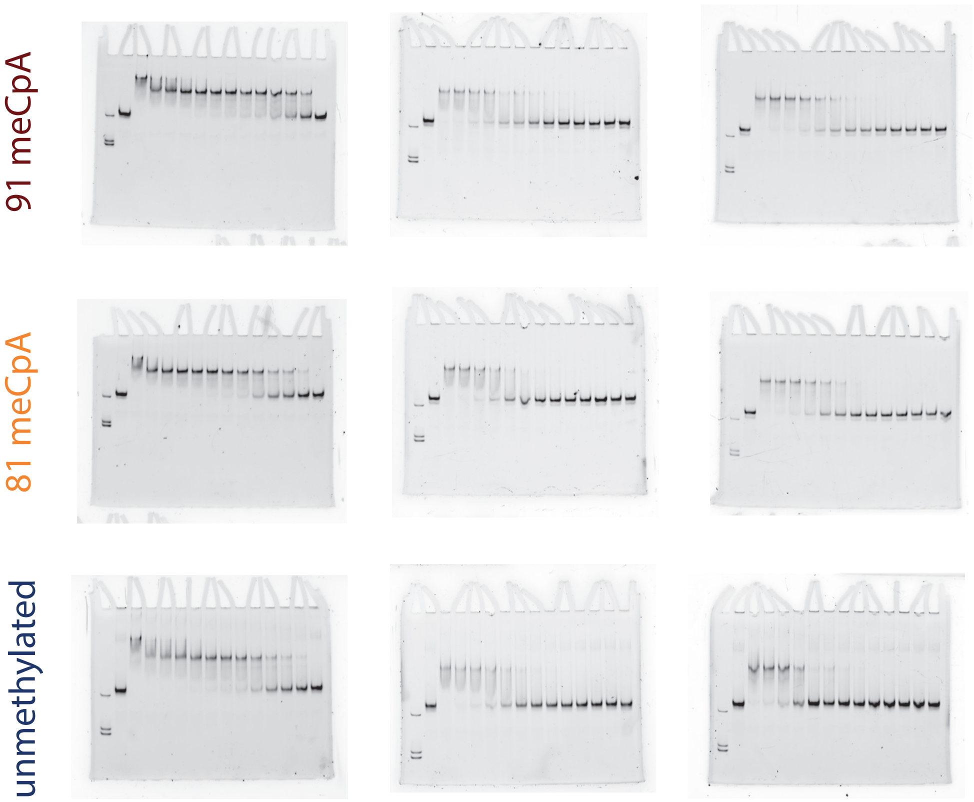

Supplementary Figure 15A

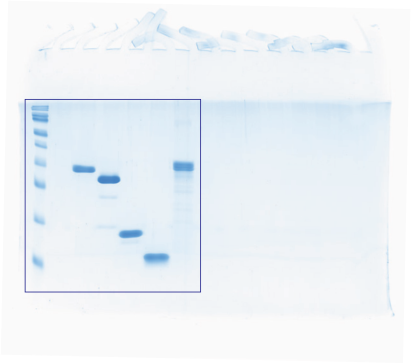

Supplementary Figure 15B

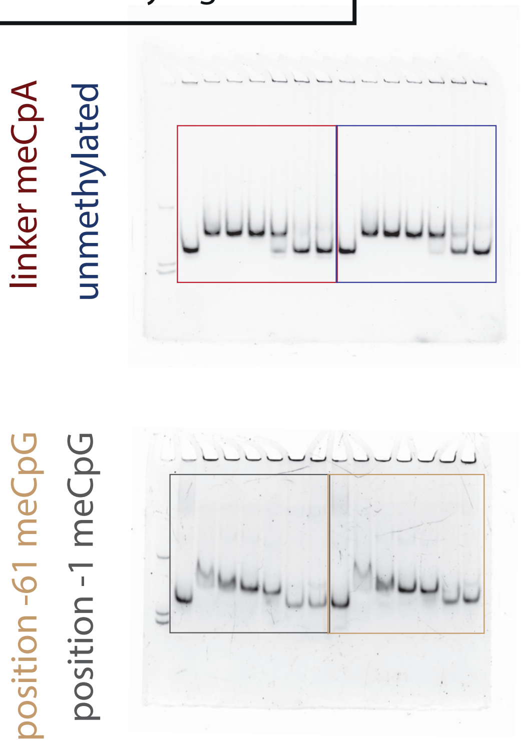

Supplementary Figure 15C

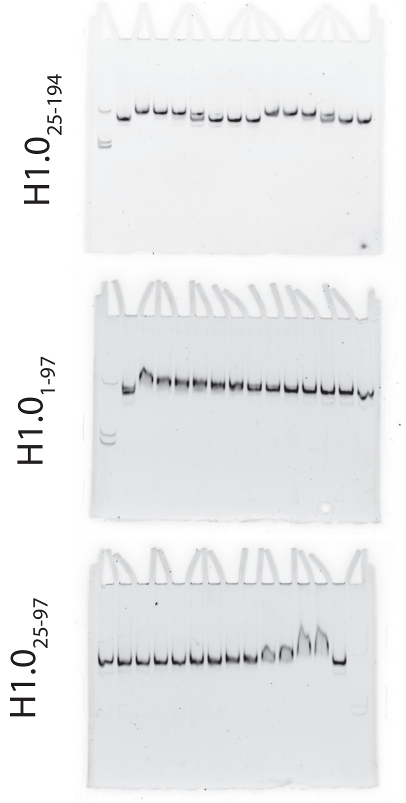

Supplementary Figure 15D

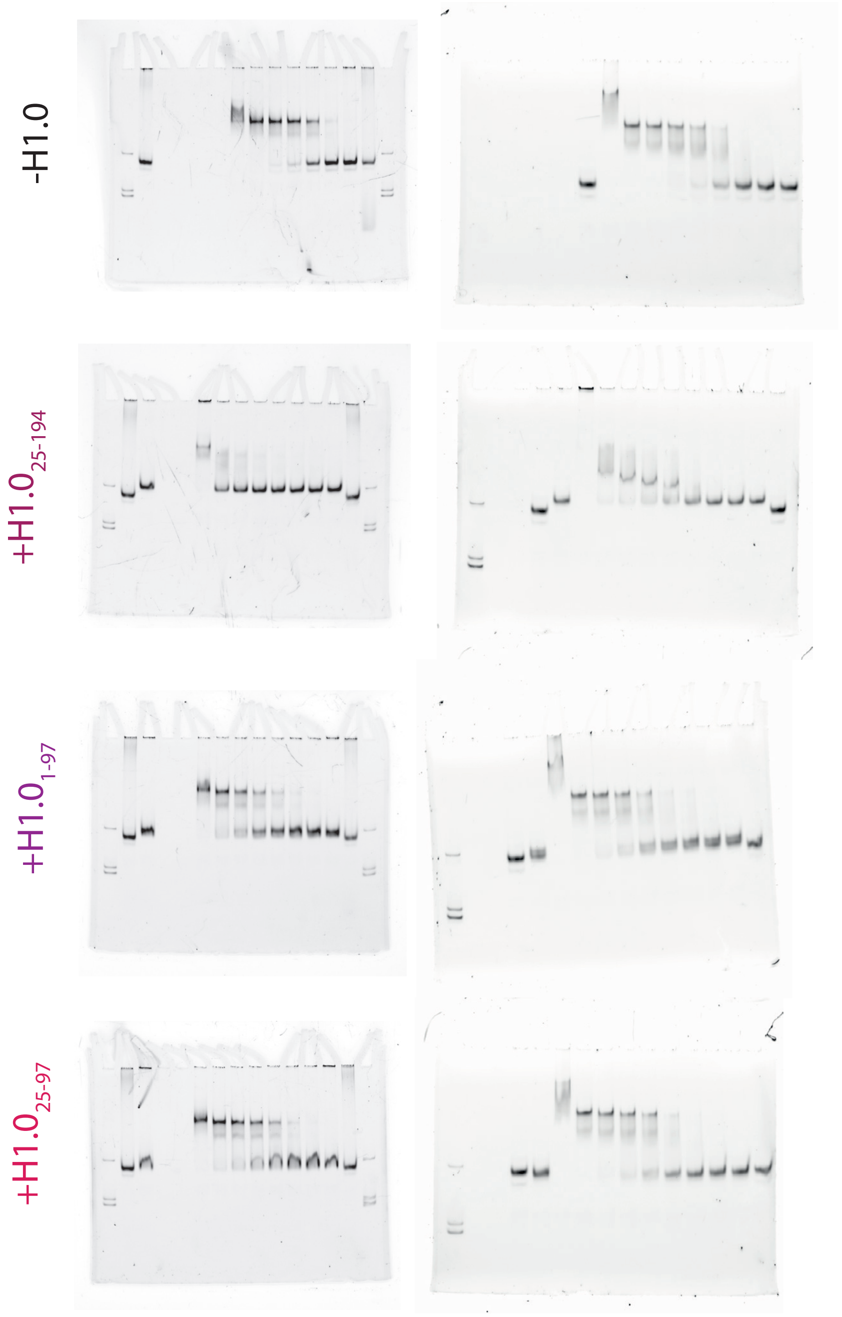

Supplementary Figure 15E

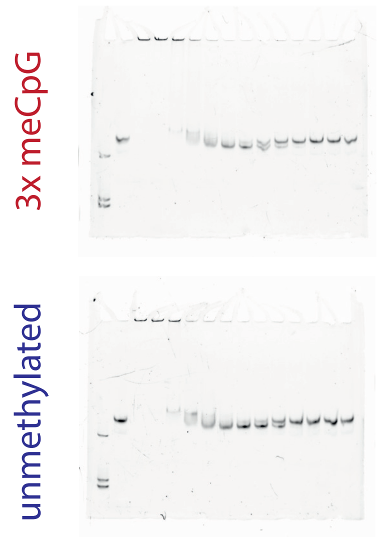

Supplementary Figure 15F

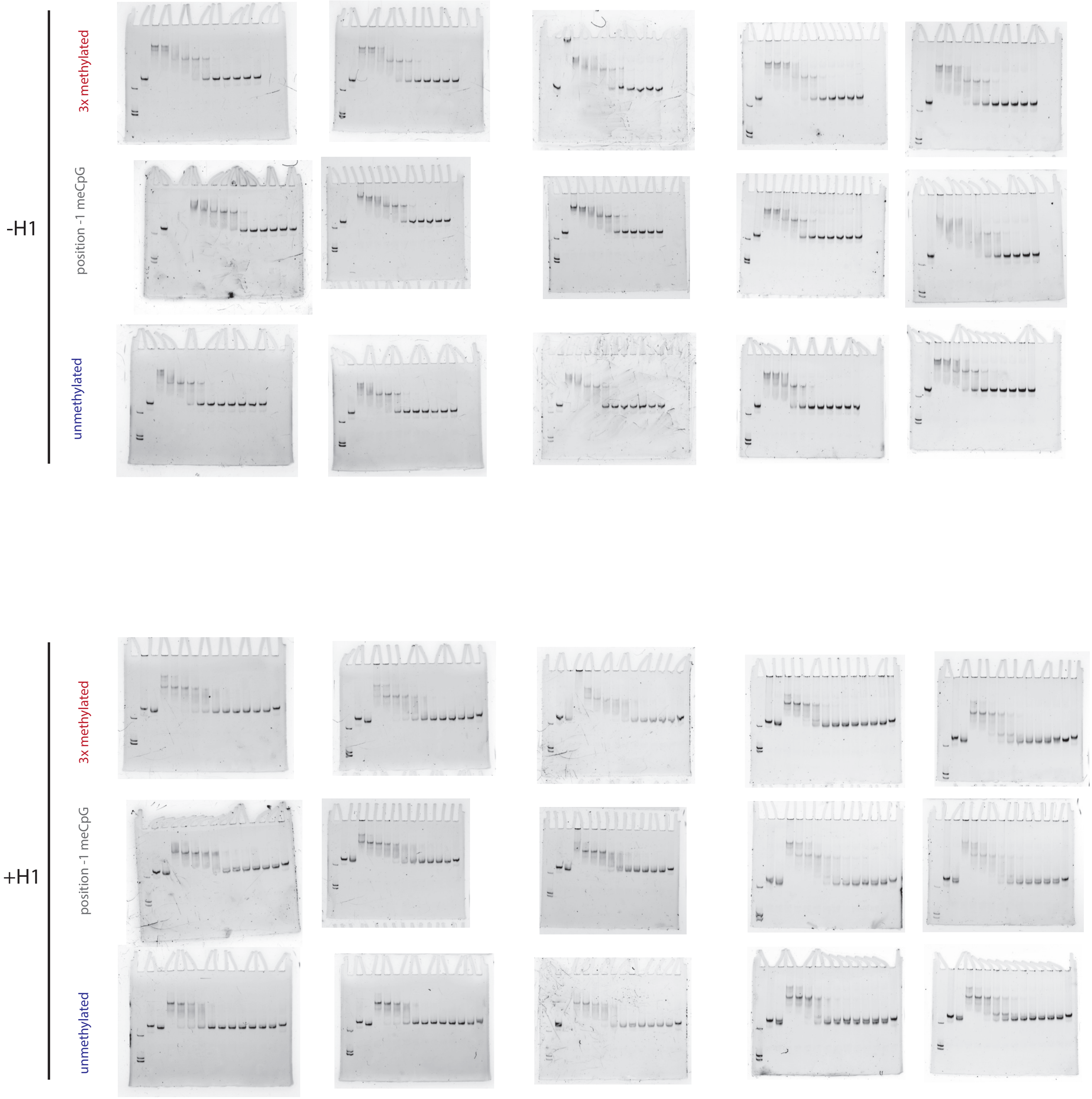

Supplementary Figure 15G

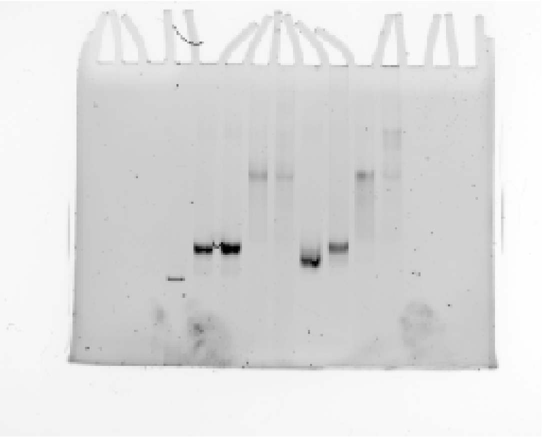

Supplementary Figure 16A

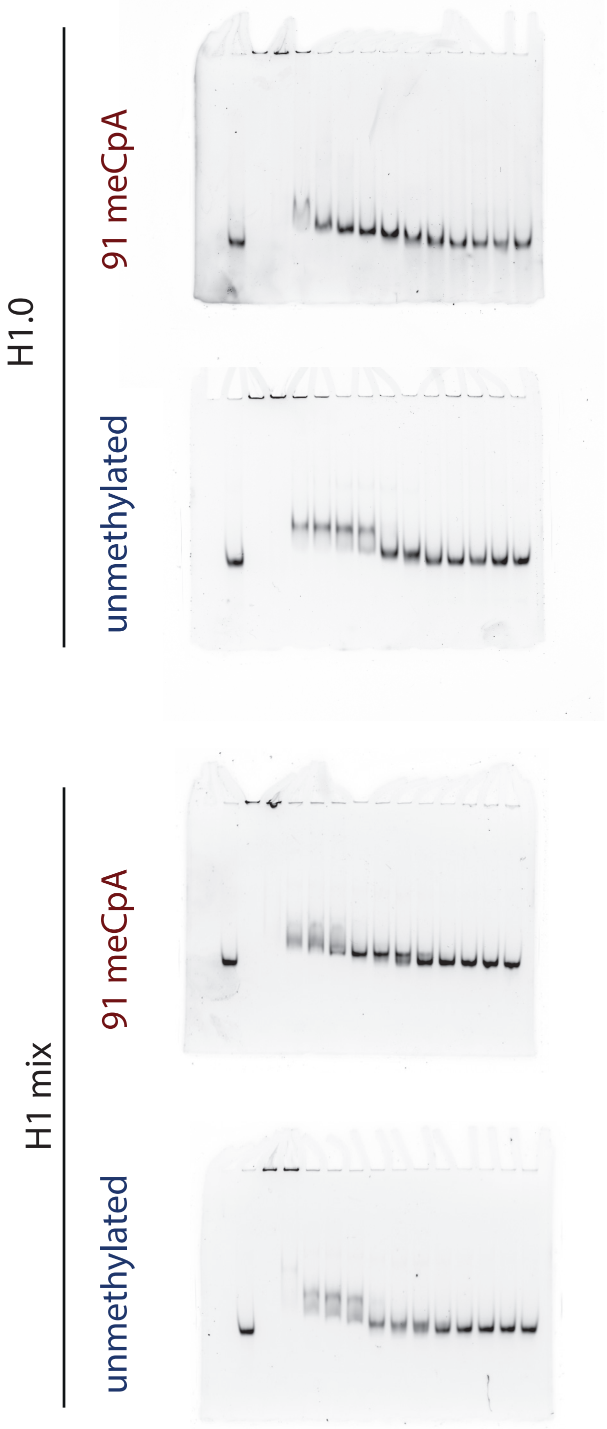

Supplementary Figure 16B

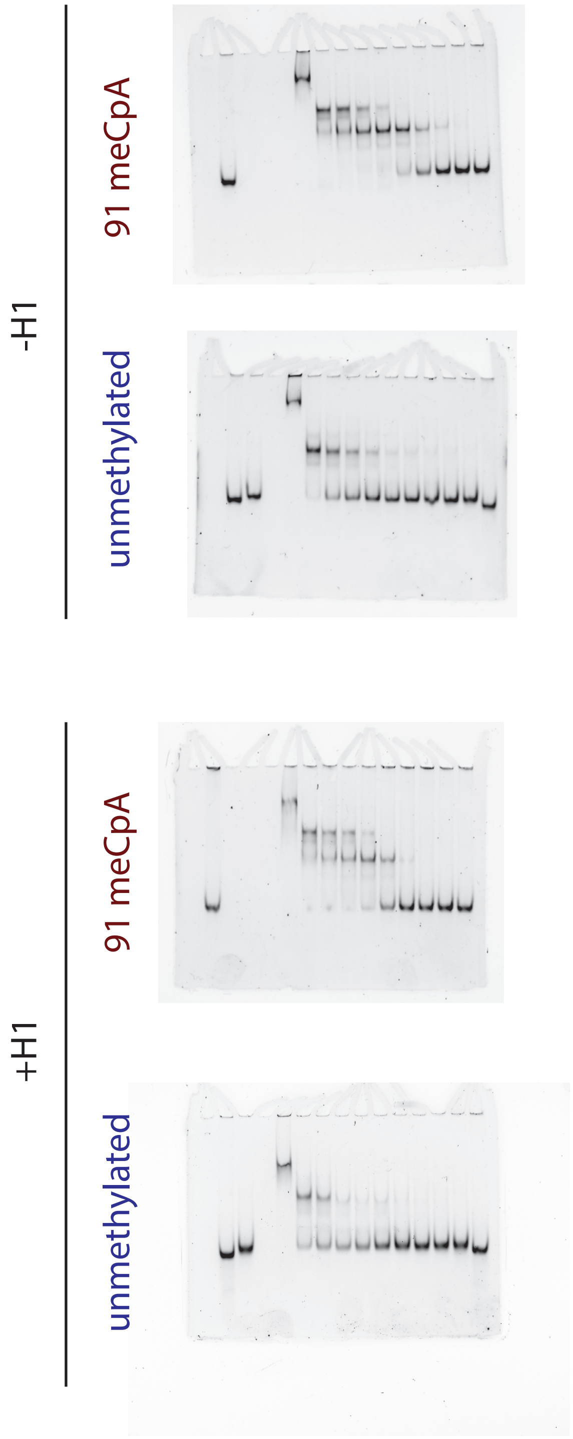

Supplementary Figure 16C

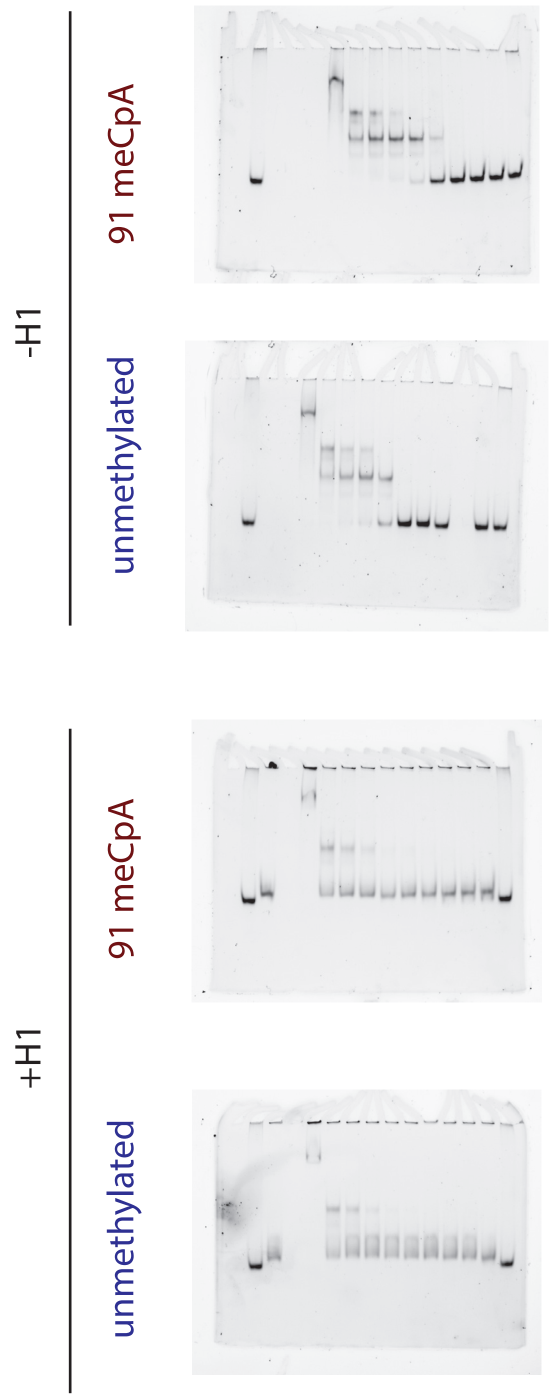

Supplementary Figure 16D

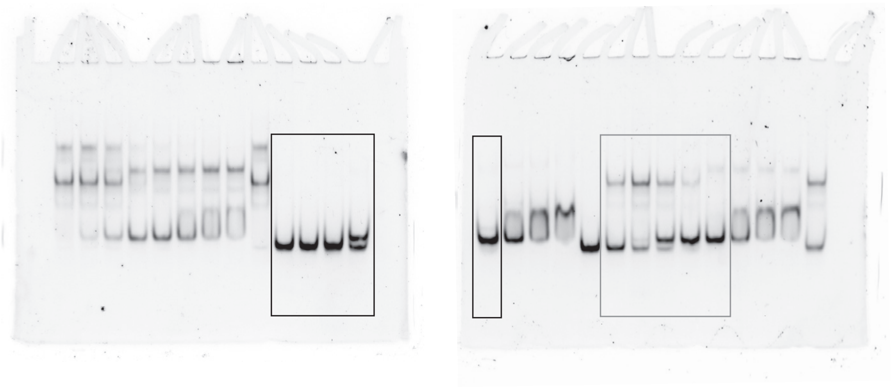

Supplement: Supplementary file 7 — Source Data File 4 [file 41467_2026_71741_MOESM7_ESM.pdf]
