# Supplementary material for: MeCP2 requires interactions with nucleosome linker DNA to read chromatin DNA methylation
Source: Nat Commun. 2026 Apr 17;17:5374. doi: 10.1038/s41467-026-71741-0 (PMC13276077; doi:10.1038/s41467-026-71741-0)

Example 1 - Fig. 1B

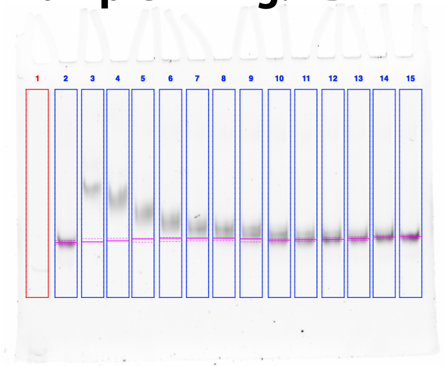

MBD binding to 37-N601-27 nucleosomes.

- Quantification of nucleosome bands close in size to complex bands.

Lane 15

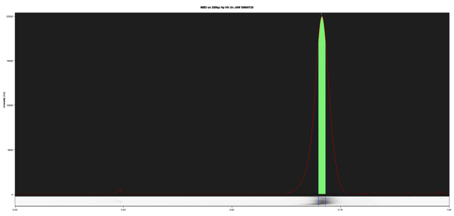

Lane 12

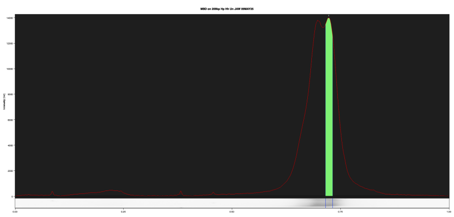

Example 2 - Fig. 1D

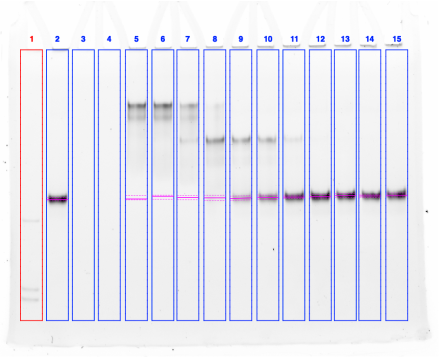

MeCP2 binding to 209bp nucleosomes.

- Quantification of standard MeCP2 binding to nucleosomes.

Lane 15

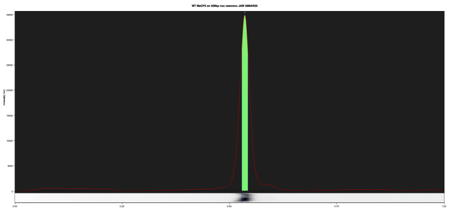

Lane 10

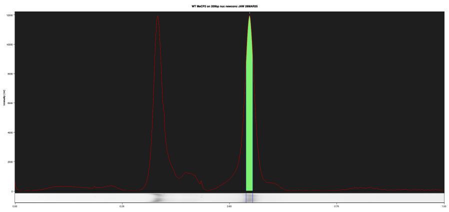

Example 3 - Fig. 5D

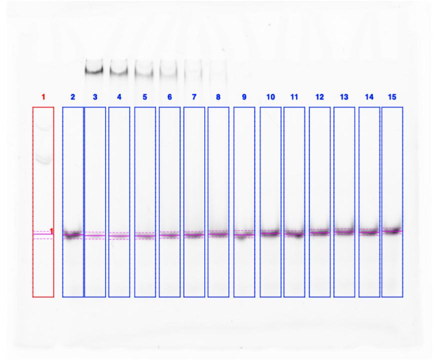

205-257 KKmut binding to 40bp DNA.

- Quantification where the highest protein concentration doesnt result in 100% fraction bound.
- In this case an additional empty lane was quantified.

Lane 15

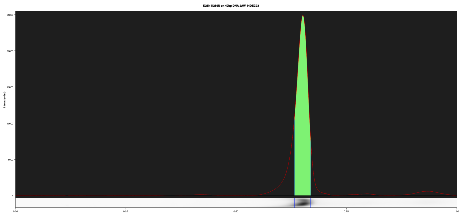

Lane 1

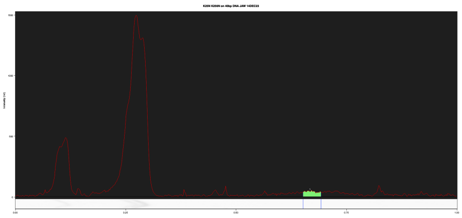

Example 4 - Fig. 7A

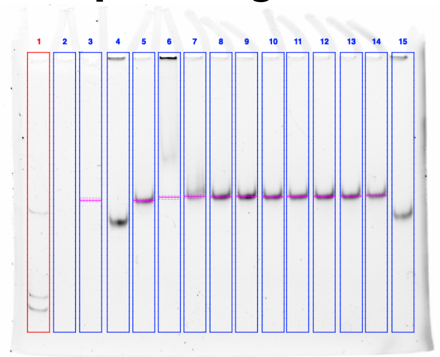

MeCP2 binding to 175bp chromatosomes.

- Quantification of chromatosome binding.
- Another example of binding not going to 100% and therefore an extra lane was quantified.

Lane 5

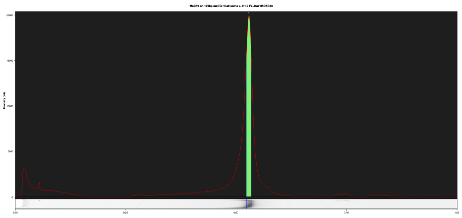

Lane 6

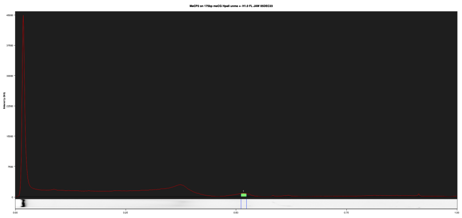

Example 5 - Sup.Fig. 8A

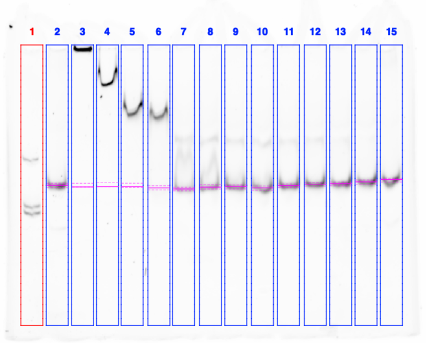

MeCP2 162-309 binding to 145bp AP nucleosomes

- Quantification where binding does not result in a clear complex band.
- Binding appears as a smear immediately above the nucleosome band.

Lane 2

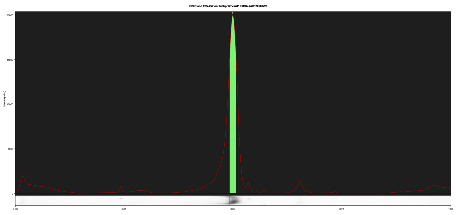

Lane 8

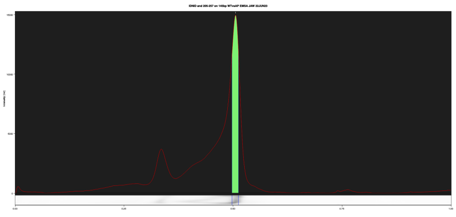

Supplement: Supplementary file 8 — Source Data File 5 [file 41467_2026_71741_MOESM8_ESM.pdf]
