# Supplementary material for: MeCP2 requires interactions with nucleosome linker DNA to read chromatin DNA methylation
Source: Nat Commun. 2026 Apr 17;17:5374. doi: 10.1038/s41467-026-71741-0 (PMC13276077; doi:10.1038/s41467-026-71741-0)
Supplement: Supplementary file 3 — Transparent Peer Review File [file 41467_2026_71741_MOESM3_ESM.pdf]

nature portfolio

Peer Review File

MeCP2 requires interactions with nucleosome linker DNA to read chromatin DNA methylation

Corresponding Author: Dr Marcus Wilson

This file contains all reviewer reports in order by version, followed by all author rebuttals in order by version.

Version 0:

Reviewer comments:

Reviewer #1

(Remarks to the Author)
MeCP2's function and regulation has received a lot of attention as it is the causal gene of RETT syndrome. This work has lead to many models, many inconclusive and mostly derived from descriptive genomic data and interaction proteomics. This manuscript is a notable exception as the study goes deep into the molecular mechanism of MeCP2 engagement providing quantitative interaction data to nucleosomal DNA with targeted DNA methylation.
Convincing evidence is provided that MeCP2 engages with individual methyl groups even if located in the nucleosomal core as long as a critical domain is contained that stabilizes binding to linker DNA which in turn replaces H1.
This adds a highly plausible mechanistic explanation how MeCP2 can engage with the genome even if methylated CpGs are sparse and challenges other models.
I find this to be a very good mechanistic study of high relevance.

Reviewer #2

(Remarks to the Author)
This manuscript from J.A. Watson and co-authors addresses important questions concerning the mechanism(s) by which MeCP2 exerts cell regulatory function, whether MeCP2 can stably bind to methylation sites located within a model nucleosome core particle. An extensive series of nucleosome constructs were created and the binding of MBD, MeCP2, and C-terminal domain deletion constructs of MeCP2 analyzed that highlight contribution of MeCP2's unstructured C-terminal domain to methylation recognition. A new C-terminal motif is identified. Additional experiments demonstrating competition of linker histone H1 and MeCP2 are presented. Several complementary in vitro and in vivo complementary studies are included that have the potential to provide important insight. However, there are deficiencies in presentation, conception and utilization of published literature, and experimental design and results interpretation that require review and revision. Below we summarize issues related to MeCP2 and MDB binding that reflect these problems but do not attempt to critique each and every of the many experiments summarized in the main text and extended results figures.

• Presentation - The manuscript is a challenge to follow by virtue of the abundance and complexity of the figures presented in both the main text and as extended data. Many of the results receive only a cursory description and are poorly documentd in many figure legends that lack essential information describing the data that is presented. The heart of the study is the binding isotherms scattered among the figures; the manuscript fails to bring these results together into consolidated figures that would allow readers to critically evaluate the authors' conclusions. Some of the data reported in Supplementary Table 3 (which includes statistics) should be organized and presented in the main text in support of the principal conclusions of these results.

It is strongly recommended that the authors remove most of the several dozen EMSA snapshots since the band patterns are independent of methylation state and the

concentration dependence of binding is documented in the isotherms. Doing so would make it easier to consolidate the critical information provided by the binding isotherms.

• Conception and utilization of published literature - Chromatin structure today is a complex, three-dimensional, well-studied system that needs to be carefully considered in interpreting the results of studies such as those presented in this manuscript. The presented results are interpreted essentially assuming that the nucleosome structure is invariant and that the linkers are linear DNA. However, the studied constructs are better considered as dynamic chromatosomes where the linker DNA of a complex depends on its sequence and/or bound proteins. The text does not consider the presented work in the context of relevant literature that in some cases supports and, in some cases, contradicts these results (e.g., https://doi.org/10.1038/s41594-024-01373-9, doi: 10.1016/j.molcel.2020.10.038, and DOI: 10.1016/j.bpj.2021.07.012).

The absence of specific consideration of the extensive literature on the DNA sequence determinants that contribute to MeCP2 binding to methylated DNA, three dimensional structures, and analysis of the MeCP2 binding thermodynamics is a serious lapse. For example, modeling bound MBD into the -1 and -61 methylation sites shown on PDB: 3LZ0 (Extended Figure 1A) would help illustrate the of the sites. Another example is that the electrostatic contribution of the C-terminal domain to MeCP2 binding has been demonstrated in many publications. In particular, page 6 states "Using full length MeCP2 increased overall affinity for meC nucleosomes, suggesting additional binding capability. However, MeCP2 also reduced the selectivity to meC compared to the MBD, suggesting this added affinity is not DNA methylation sensitive." implying that this is a property unique to nucleosome binding. It's not. Published studies demonstrated these properties with free DNA, independent of a nucleosome (e.g., DOI: 10.1021/acs.biochem.6b00451). While there are important nuances to the C-terminal mediated interactions, including the work presented in this article, the driving force behind them remains stolidly electrostatic.

• Experimental design and results interpretation Several overall aspects of the experimental design are problematic: 1) No controls are shown for the duplex DNA constructs without histones. No control with a single modification in one or the other linker is analyzed in the main text figures; 2) A systematic analysis of the helical phase dependence and nucleosome position dependence was not conducted; 3) The sizable Hill coefficients measured for MeCP2 (previously reported in the above referenced article) are not considered in the interpretation of the mechanism of MeCP2 binding; 4) The ionic strength ranges from 125 to 200mM as well as different excipients among the methods used in this study. These differences (especially ionic strength) are likely to profoundly impact the results of the experiments and undercut the validity of the integration of the orthogonal techniques; and 5) No direct evidence is presented that MeCP2 physically contacts the methylated or unmethylated sites. The rationale underlying these concerns is elaborated below:

1) The appropriate reference state for the presented binding studies is each DNA template in the absence of histones. Such measurements must have been done in the development of the project; it is perplexing why they are not shown. An additional informative control would be a single methylation site within the linker DNA. The +83 site seems suitable however its analysis together with -61 and -80 undercuts its use as a control (Figure 1D, annotated 3X). The leftward shift of the 3X construct is likely to be partly or mostly due to the tripling of the number of binding sites. The -61 construct is not analyzed in the 37-N601-27 context. Why?

2) The authors analyze just two methylation sites within the nucleosome core. The first is at the dyad (-1) and the second (-61) towards the end of the linear strand. Although the dinucleotides are colored in Extended Figure 1A, the solvent accessibilities of these sites have not been calculated. This calculation should consider modeling the published three-dimensional structure of MDB – mCpG complexes in order to provide a physically meaningful metric of accessibility. In addition, the manuscript does not explicitly state the DNA sequence of each site. If they are not identical than comparison of their binding affinity for MeCP2 cannot be fully ascribed to solvent accessibility. The ambiguity of the actual accessibility undercuts the statement made in the introduction (page 4) that "We find that MeCP2 can bind DNA methylation on the nucleosome surface, even when meC sites are facing the histone octamer, which would be predicted to block binding."
Stronger support for this assertion could be developed by conducting a systemic helical analysis of methylation sites preferably at multiple locations around the nucleosome. As can be seen in pictures shown in Extended Figure 1A, DNA wrapping around the histone octamer is not perfectly uniform. Characterization of the MeCP2

- nucleosome core interactions is key to understanding the C-terminal mediated interactions. Perhaps most importantly, the nucleosome is dynamic. The authors need to consider fluctuations in the histone octamer – DNA interactions in interpreting their results.

The proposed model (Figure 7) predicts that core methylation sites that are only within 'touching' distance of one of the two linkers will be productively bound. This aspect of the model is not tested by the presented data. Also, given that 'half' the wrapped DNA is fully exposed on the surface of the histone octamer and that the Cterminal interaction is dominated by electrostatics, it is surprising that core methylation sites that are solvent accessible cannot be detectably bound. This conundrum should at a minimum, be discussed. The nature of the C-terminal domain – DNA interaction should be more thoroughly discussed.
The hydroxyl radical footprinting analysis shown in Extended Figure 1B is a thoughtful and potentially insightful probe of changes in DNA structure as a function of methylation. However, these data are cursorily annotated and unanalyzed except for noting the 10 bp periodicity. The strand analyzed is not cited in the figure legend, the numbering is in bp while the constructs are annotated in relation to the dyad, and the exact position each binding site not clearly indicated on the traces. This housekeeping aside, it is very intriguing that periodicity is lost in both methylated constructs between nucleotides 100 -115. A simple difference calculation would clearly show this change; a rationalization for this result is that methylation alters winding of the DNA at a distance. This observation suggests that DNA methylation alone has 'action at a distance' on the structure of the nucleosome core particle.
3) The binding isotherm present in this paper are of outstanding quality. (The gels that were quantitated should be documented in the supplementary results including several examples of the boxes used to quantitate the band densities.) However, no consideration is given to the sizable and variable Hill coefficients that may reflect differences in the underlying MeCP2 binding mechanism(s) that are very likely more complex than the simple cartoons presented in the paper.

4) As noted above, a significant dependence of MeCP2 binding on salt concentration is documented in the literature. Thus, the differences in the excipients used for the different experimental techniques is of concern. While the authors are applauded for including a Mass Photometry analysis (Extended Figure 2C) it is of concern that it was conducted at 200 mM salt compared with 125 mM salt used in EMSA. It is likely that the mass distributions will be both more complex and larger at the lower salt concentrations of the binding assays based up the sizable Hill coefficients measured by EMSA. If so, the Mass Photometry analysis at 200 mM salt provides an inaccurate picture of the binding species present in the EMSA experiments.
In addition, the mass photometry analysis should include the controls noted above for the EMSA studies. The information provided in the legend is cursory. The expected and measured (with error analysis) molecular weights should be stated. Eyeballing the nucleosome core particle at 215 kDa with MeCP2 about 52 kDa adds up to 267 which is consistent with a single MeCP2 monomer binding to a symmetrically modified site. However, the cartoons in Figure 7 appears to show MeCP2 can bind as a dimer. (The legend to this figure is uninformative.) This apparent discrepancy needs to be clarified.

5) The conclusion that MeCP2 binds to methylated sites within the nucleosome core is inferred from the binding isotherms but not directly demonstrated. Given that this observation is the keystone of the whole study, the absence of direct evidence of protection of bound DNA by MeCP2 is a critical deficiency. Since the authors have conducted hydroxyl radical footprinting of the nucleosome, this technique would seem to be the easiest way to obtain this information. Alternatively, DNase 1 footprinting, which was used long ago to map the helical periodicity of DNA wrapper around the histone core, should also be able to provide this information. This analysis should be straightforward for the MBD domain. Mapping the C-terminal contacts is likely to be a more significant technical challenge but would provide valuable support for the manuscripts guiding hypothesis.

Reviewer #4

(Remarks to the Author)
This is a strong and well-executed manuscript that significantly contributes to our understanding of how MeCP2 recognizes methylated DNA in the context of chromatin. The authors build on earlier reports of a secondary DNA-binding region in the central domain of MeCP2 and convincingly show that this region plays a critical role in enabling methylation sensing within nucleosomes. By demonstrating that this mechanism relies on the presence and length of linker DNA, the study adds an important structural dimension to our understanding of MeCP2 function.

The findings are well aligned with recent work on chromatin-binding proteins, particularly those containing intrinsically disordered regions. Beyond MeCP2 itself, the study has broader implications for how disordered chromatin readers interpret epigenetic marks in compacted DNA, and how features like nucleosome positioning and linker accessibility can modulate that process.

Mechanistically, the data support a model in which multivalent interactions—mediated by both the canonical MBD and a second DNA-binding site—allow MeCP2 to access methylated CpG sites that are otherwise occluded within the nucleosomal core. This is a meaningful addition to our current understanding of MeCP2 function and helps explain why truncated variants lacking this central domain often fail to restore full activity in Rett syndrome models. It also opens the door to refining therapeutic strategies that aim to restore MeCP2 function.

The experimental approach is thorough and well controlled. The combination of in vitro assays (EMSA, MST, SPR) with live-cell imaging (FRAP) provides a solid foundation for the conclusions. Results are consistent across methodologies and clearly support the idea that both methylation and linker DNA are required for proper MeCP2–chromatin engagement.

That said, there are a few points that I believe would benefit from clarification or further discussion:
1. Direct histone interaction
Previous studies have shown that MeCP2 can interact directly with histones, including their tails. In this manuscript, it's not clear whether any controls were included using histone octamers in the absence of DNA. If such experiments were not performed, I would suggest acknowledging this as a limitation.
2. Binding to core particles
A central observation in the manuscript is that MeCP2 fails to bind nucleosomes lacking linker DNA. While this supports the proposed model where linker interactions are essential for methylation sensing, it raises an unresolved question: if MeCP2 is capable of binding both DNA and histones—as suggested by previous studies—why is there no detectable binding to core nucleosomes in the absence of linker DNA?. I encourage the authors to address this apparent discrepancy more directly, and to discuss whether it challenges or refines current models of MeCP2–chromatin association.
3. Interpretation of binding affinities
EMSA is widely used to quantify protein–DNA interactions, but its ability to distinguish true affinity from cooperative or multivalent effects is limited. Since MeCP2 has been described (here and elsewhere) to bind DNA through multiple motifs, it would be helpful to state whether the Kd values were calculated assuming a simple one-site model, or if multivalency was taken into account in any way, and discuss what would the consequences of overlooking this aspect be.
4. Reference for R133G
The manuscript mentions that the R133G mutation disrupts methylation recognition via the MBD. Please include a supporting reference for this or provide additional data in the supplement.

In summary, this is a well-written and impactful study that makes a clear and meaningful contribution to the field. Addressing the points above would strengthen the mechanistic model and improve interpretability, particularly around the limitations of the experimental design. I support publication pending minor revisions.

Reviewer #5

(Remarks to the Author)
I co-reviewed this manuscript with one of the reviewers who provided the listed reports. This is part of the Nature Communications initiative to facilitate training in peer review and to provide appropriate recognition for Early Career Researchers who co-review manuscripts.

Version 1:

Reviewer comments:

Reviewer #2

(Remarks to the Author)
We have no further comments to convey to the authors.

Reviewer #4

(Remarks to the Author)
I appreciate the authors' thoughtful and detailed responses to the concerns raised in my initial review. The additional data and clarifications they have provided strengthen the manuscript and help address several key points of interpretation and methodology.

Thank you for addressing the point regarding MeCP2–histone interactions. I understand the technical limitations in assaying octamer binding without DNA, and I find the expanded discussion in the revised manuscript appropriate. The idea that MeCP2 may recognize DNA and histone features individually, but that these are masked or altered in the nucleosomal context, is plausible. The proposed contribution of linker geometry to multivalent engagement adds useful mechanistic insight. I consider the issue adequately addressed given the current experimental scope.

On the topic of binding affinity measurements, I find the use of apparent Kd values and internal comparisons reasonable, and I appreciate that the authors have now described the use of the Hill equation more explicitly. The variability in Hill coefficients does suggest a complex and multivalent binding mode, which is consistent with the molecular features of MeCP2. These additions help improve the transparency and reproducibility of the data interpretation.

The inclusion of new binding data for the R133G mutation is helpful. Regarding the key role of the residue in methylaiton recognition, The mutation was likely to have the said effect, but having experimental data backing this up is highly necesary.
Keynote: There is a typo in page 14 line 394: It should be "MeCP2 can RECOGNISE...." instead of "recognises".

In summary, I believe the authors have addressed the main concerns raised during the first round of review with rigor and transpa I support acceptance of this revised version.rency. The additional experiments and revisions improve the manuscript both conceptually and technically.

Reviewer #5

(Remarks to the Author)
I co-reviewed this manuscript with one of the reviewers who provided the listed reports. This is part of the Nature Communications initiative to facilitate training in peer review and to provide appropriate recognition for Early Career Researchers who co-review manuscripts.

Open Access This Peer Review File is licensed under a Creative Commons Attribution 4.0 International License, which permits use, sharing, adaptation, distribution and reproduction in any medium or format, as long as you give appropriate credit to the original author(s) and the source, provide a link to the Creative Commons license, and indicate if changes were made.
In cases where reviewers are anonymous, credit should be given to 'Anonymous Referee' and the source.
The images or other third party material in this Peer Review File are included in the article’s Creative Commons license, unless indicated otherwise in a credit line to the material. If material is not included in the article’s Creative Commons license and your intended use is not permitted by statutory regulation or exceeds the permitted use, you will need to obtain permission directly from the copyright holder.
To view a copy of this license, visit https://creativecommons.org/licenses/by/4.0/

RESPONSE TO REVIEWER COMMENTS

Reviewer #1 (Remarks to the Author):

MeCP2's function and regulation has received a lot of attention as it is the causal gene of RETT syndrome. This work has lead to many models, many inconclusive and mostly derived from descriptive genomic data and interaction proteomics.
This manuscript is a notable exception as the study goes deep into the molecular mechanism of MeCP2 engagement providing quantitative interaction data to nucleosomal DNA with targeted DNA methylation.
Convincing evidence is provided that MeCP2 engages with individual methyl groups even if located in the nucleosomal core as long as a critical domain is contained that stabilizes binding to linker DNA which in turn replaces H1.
This adds a highly plausible mechanistic explanation how MeCP2 can engage with the genome even if methylated CpGs are sparse and challenges other models.
I find this to be a very good mechanistic study of high relevance.

We thank the reviewer for the positive appraisal and insightful comments.

Reviewer #2 (Remarks to the Author):

This manuscript from J.A. Watson and co-authors addresses important questions concerning the mechanism(s) by which MeCP2 exerts cell regulatory function, whether MeCP2 can stably bind to methylation sites located within a model nucleosome core particle. An extensive series of nucleosome constructs were created and the binding of MBD, MeCP2, and C-terminal domain deletion constructs of MeCP2 analyzed that highlight contribution of MeCP2's unstructured C-terminal domain to methylation recognition. A new C-terminal motif is identified. Additional experiments demonstrating competition of linker histone H1 and MeCP2 are presented. Several complementary in vitro and in vivo complementary studies are included that have the potential to provide important insight.

However, there are deficiencies in presentation, conception and utilization of published literature, and experimental design and results interpretation that require review and revision. Below we summarize issues related to MeCP2 and MDB binding that reflect these problems but do not attempt to critique each and every of the many experiments summarized in the main text and extended results figures.

We thank the reviewer for the highly detailed and extensive comments, as well as compliments on our data. Clearly there were some misconceptions based on the way we wrote the manuscript, and we are grateful to the reviewer for pointing these out, allowing us to reword the text. However, we highlight that several of the comments are at odds with the supportive statements of reviewer 1 and 4. In some cases it was difficult for us to understand the points in the review, sometimes due to typographical errors. Nevertheless this feedback also encouraged us to expand upon our results, by inclusion of new data, that has strengthened the manuscript.

A) Presentation - The manuscript is a challenge to follow by virtue of the abundance and complexity of the figures presented in both the main text and as extended data. Many of the results receive only a cursory description and are poorly documentd in many figure legends that lack essential information describing the data that is presented. The heart of the study is the binding isotherms scattered among the figures; the manuscript fails to bring these results together into consolidated figures that would allow readers to critically evaluate the authors' conclusions. Some of the data reported in Supplementary Table 3 (which includes statistics) should be organized and presented in the main text in support of the principal conclusions of these results.

It is strongly recommended that the authors remove most of the several dozen EMSA snapshots since the band patterns are independent of methylation state and the concentration dependence of binding is documented in the isotherms. Doing so would make it easier to consolidate the critical information provided by the binding isotherms.

As suggested, we have now moved relevant information from Supplementary Table 3 into the main text (Table 1), and have removed some of the EMSA gels from the main figures into the supplemental files. Please see the figure changes summary at the bottom of this document for full details of these changes. Figure legends have been expanded throughout.

There is information in the gel images beyond the bound fraction, with discrete bands that are important for the evaluation of the data, which we have left where appropriate.

B) Conception and utilization of published literature - Chromatin structure today is a complex, three-dimensional, well-studied system that needs to be carefully considered in interpreting the results of studies such as those presented in this manuscript. The

presented results are interpreted essentially assuming that the nucleosome structure is invariant and that the linkers are linear DNA. However, the studied constructs are better considered as dynamic chromatosomes where the linker DNA of a complex depends on its sequence and/or bound proteins. The text does not consider the presented work in the context of relevant literature that in some cases supports and, in some cases, contradicts these results (e.g., https://doi.org/10.1038/s41594-024-01373-9, doi:10.1016/j.molcel.2020.10.038, and DOI: 10.1016/j.bpj.2021.07.012). The absence of specific consideration of the extensive literature on the DNA sequence determinants that contribute to MeCP2 binding to methylated DNA, three dimensional structures, and analysis of the MeCP2 binding thermodynamics is a serious lapse. For example, modeling bound MBD into the -1 and -61 methylation sites shown on PDB: 3LZ0 (Extended Figure 1A) would help illustrate the of the sites.

The dynamic nature of DNA within chromatin and the nucleosome is an important point, especially considering the suggested modelling (included now in Supplementary Figure 2C, 4A), in which a static view of nucleosome could prevent the MBD-meC interaction. In combination with additional data, we have extended discussion of the role of dynamics and DNA sequence of MeCP2 on page 6, 7, 13 and page 15 of the revised manuscript.
The studies highlighted here were either considered extensively and discussed in relation to our findings (Chua et al., cited 8 times) or beyond the scope of this manuscript as they are concerning only H1 rather than MeCP2.

Another example is that the electrostatic contribution of the C-terminal domain to MeCP2 binding has been demonstrated in many publications. In particular, page 6 states "Using full length
MeCP2 increased overall affinity for meC nucleosomes, suggesting additional binding capability. However, MeCP2 also reduced the selectivity to meC compared to the MBD, suggesting this added affinity is not DNA methylation sensitive." implying that this is a property unique to nucleosome binding. It's not. Published studies demonstrated these properties with free DNA, independent of a nucleosome (e.g., DOI: 10.1021/acs.biochem.6b00451). While there are important nuances to the C-terminal mediated interactions, including the work presented in this article, the driving force behind them remains stolidly electrostatic.

We had not meant to imply this was a unique nucleosome feature and have reworded the section to help clarify this (page 6). The original text was written focusing on describing our results in this section. Indeed, the DNA binding of regions outside of the MBD was originally mentioned extensively with citations in the original manuscript in the Introduction page 4 line 19-23, again in results bottom page 7 and in the discussion on page 14.

We apologise for missing this relevant reference and have expanded to include this and further references throughout.

C) Experimental design and results interpretation Several overall aspects of the experimental design are problematic: 1) No controls are shown for the duplex DNA constructs without histones. No control with a single modification in one or the other linker is analyzed in the main text figures; 2) A systematic analysis of the helical phase dependence and nucleosome position dependence was not conducted; 3) The sizable Hill coefficients measured for MeCP2 (previously reported in the above referenced article) are not considered in the interpretation of the mechanism of MeCP2 binding; 4) The ionic strength ranges from 125 to 200mM as well as different excipients among the methods used in this study. These differences (especially ionic strength) are likely to profoundly impact the results of the experiments and undercut the validity of the

integration of the orthogonal techniques; and 5) No direct evidence is presented that MeCP2 physically contacts the methylated or unmethylated sites. The rationale underlying these concerns is elaborated below:

1) The appropriate reference state for the presented binding studies is each DNA template in the absence of histones. Such measurements must have been done in the development of the project; it is perplexing why they are not shown An additional informative control would be a single methylation site within the linker DNA.. The +83 site seems suitable however its analysis together with -61 and -80 undercuts its use as a control (Figure 1D, annotated 3X). The leftward shift of the 3X construct is likely to be partly or mostly due to the tripling of the number of binding sites. The -61 construct is not analyzed in the 37-N601-27 context. Why?

Data of MeCP2 DNA binding in the absence of nucleosomes has now been included in Supplementary Figure 2E and 5A.

Data showing a single methylation site within the linker region was presented in Extended data Figure 2A&B, and has now been made more prominent in the new main Figure 2C&D.

We agree the additional binding was due to the presence of multiple methylated cytosines in this construct and commented on this in the discussion on page 13. Site-specific DNA methylation was generated either through direct synthesis in DNA oligos used for PCR amplification (meCA sites), or by HhaI or HpaII CpG methyltransferases with specific recognition sequences. The 37-N601-27 construct contains 3 such HpaII recognition sites including the -61 position. We further probed the effect of this exact site of modification in isolation in the short 15-N601-15 construct.

2) The authors analyze just two methylation sites within the nucleosome core. The first is at the dyad (-1) and the second (-61) towards the end of the linear strand. Although the dinucleotides are colored in Extended Figure 1A, the solvent accessibilities of these sites have not been calculated. This calculation should consider modeling the published three-dimensional structure of MDB – mCpG complexes in order to provide a physically meaningful metric of accessibility. In addition, the manuscript does not explicitly state the DNA sequence of each site. If they are not identical than comparison of their binding affinity for MeCP2 cannot be fully ascribed to solvent accessibility. The ambiguity of the actual accessibility undercuts the statement made in the introduction (page 4) that “We find that MeCP2 can bind DNA methylation on the nucleosome surface, even when meC sites are facing the histone octamer, which would be predicted to block binding.” Stronger support for this assertion could be developed by conducting a systemic helical analysis of methylation sites preferably at multiple locations around the nucleosome. As can be seen in pictures shown in Extended Figure 1A, DNA wrapping around the histone octamer is not perfectly uniform. Characterization of the MeCP2 - nucleosome core interactions is key to understanding the C-terminal mediated interactions. Perhaps most importantly, the nucleosome is dynamic. The authors need to consider fluctuations in the histone octamer – DNA interactions in interpreting their results.

The proposed model (Figure 7) predicts that core methylation sites that are only within ‘touching’ distance of one of the two linkers will be productively bound. This aspect of the model is not tested by the presented data. Also, given that ‘half’ the wrapped DNA is fully exposed on the surface of the histone octamer and that the Cterminal interaction is dominated by electrostatics, it is surprising that core methylation sites that are solvent accessible cannot be detectably bound. This

conundrum should at a minimum, be discussed. The nature of the C-terminal domain – DNA interaction should be more thoroughly discussed.
The hydroxyl radical footprinting analysis shown in Extended Figure 1B is a thoughtful and potentially insightful probe of changes in DNA structure as a function of methylation. However, these data are cursorily annotated and unanalyzed except for noting the 10 bp periodicity. The strand analyzed is not cited in the figure legend, the numbering is in bp while the constructs are annotated in relation to the dyad, and the exact position each binding site not clearly indicated on the traces. This housekeeping aside, it is very intriguing that periodicity is lost in both methylated constructs between nucleotides 100 -115. A simple difference calculation would clearly show this change; a rationalization for this result is that methylation alters winding of the DNA at a distance. This observation suggests that DNA methylation alone has ‘action at a distance’ on the structure of the nucleosome core particle.

These are important points and ones we had considered, but have now added additional data and discussion about in the revised manuscript. The two core methylation sites are expected to be blocked sterically with poor solvent accessibility (now included modelling and accessibility scores in Supplementary Figure 2C&D).

As suggested, we have further annotated and discussed the hydroxyl-radical footprinting data in Supplementary Figure 1D. The additional protection observed at 100-115 (15-25 bp from the dyad) is intriguing but does not appear to be reproducible (Reviewer Figure 1) nor present on longer constructs with newer sequencing strategy for the footprinting assay that we have now mapped (Supplementary Figure 1D). On these longer constructs the position of fluorescent-readout is further away from nucleosome core, reducing information loss of protection from any excess uncleaved signal.

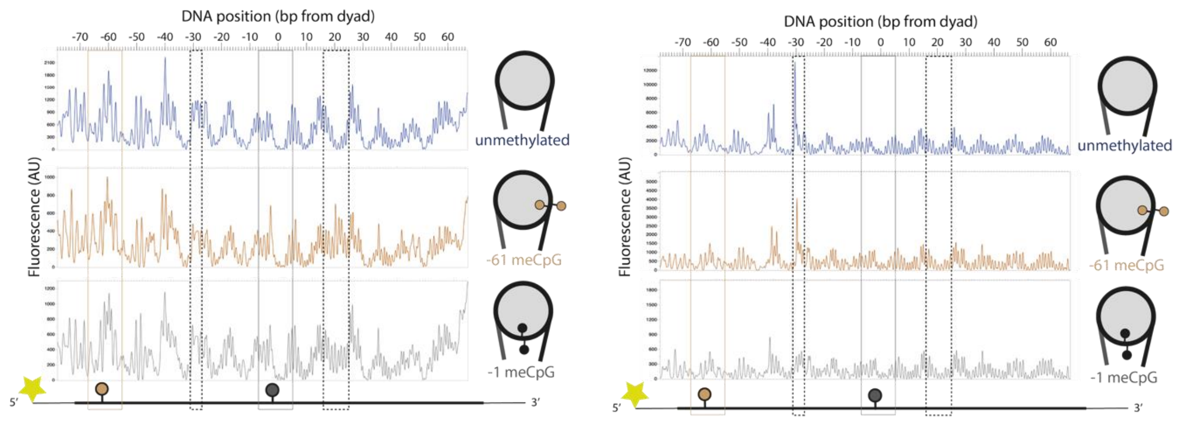

Reviewer Figure 1: Repeat (right) of the hydroxyl-radical footprinting originally shown in Extended Data 1B (left). We note a decrease in protection 15-25 bp from the dyad in the original experiment (black dotted line) in two nucleosome samples, which is not seen in the repeat. Similarly a large peak is observed ~ -30 bp from the dyad in both methylated and unmethylated data, which is not seen in the original (black dotted line). We believe this to be experimental, possibly due to bleed through from previous degradation of the molecular weight standards used to calibrate the capillary assay and not observed from more recent optimised data.

We have added further discussion of the DNA interaction of the identified MeCP2 central region on page 15. Background binding to NCPs lacking linker regions can be observed but this is removed by adding competitor dsDNA. As suggested the addition of short linker regions (30 bp worth of charge) would not be expected to outcompete roughly half of the charge accessible from 145bp of DNA wrapped around the nucleosome, suggesting there is a degree of specificity for dsDNA linkers. These findings suggest MeCP2 binding is not totally driven by electrostatic interaction to negative charge.

We have included experiments testing 4 further nucleosome core methylation sites (New Figure 2A & B), with differing local sequence, distance to the linker regions and proposed accessibility based on modelling. MeCP2 retains similar methylation preference on these nucleosomes, albeit with altered overall affinities likely due to DNA sequence differences as suggested by the reviewer. As mentioned previously the nature of local sequence around methylated cytosine locations is experimentally driven by the DNA methyltransferase recognition sites, we present the local sequence contexts in Supplementary Figure 1A, 4B&C, and 5A, in addition to the full sequence used (Supplementary Table 2). We have now elaborated on these experiments and their findings in the result (page 6) and discussion (page 13) sections.

Motivated by these findings we are excited to follow up this work with a full systematic analysis of meC sites within the nucleosome core. Here we are reporting the unexpected phenomena that methylated cytosines can be read within the nucleosome core, we will follow this up with a full analysis but this would require ~150 constructs to be made, enzymatically methylated, wrapped in nucleosomes, quality controlled and then tested. This is simply beyond the scope of the current manuscript.

3) The binding isotherm present in this paper are of outstanding quality. (The gels that were quantitated should be documented in the supplementary results including several examples of the boxes used to quantitate the band densities.) However, no consideration is given to the sizable and variable Hill coefficients that may reflect differences in the underlying MeCP2 binding mechanism(s) that are very likely more complex than the simple cartoons presented in the paper.

Thank you for the compliment on the quality of the data. Gels have now been included as source data files 2 and 4 as suggested, as well as examples of how the band densities were quantified in source data file 5.

Regarding Hill coefficients, we have added in discussion of this where appropriate throughout the results (pages 6, 7, 8 and 10) and discussion (page 14) sections, which has helped to clarify the predicted multivalent co-operative nature of binding. As mentioned by reviewer 3 the binding is multivalent and highly complex. This is in part to integrate multiple possibly co-operative binding modes, and the effect of competitor DNA interaction used throughout in assays. All experiments were performed with appropriate controls to allow direct comparison (see reviewer 2 point B). We were therefore reticent to over-interpret the data given these caveats (i.e not reporting an absolute Kd but instead an apparent Kd in internally controlled assays). The final model is of course an oversimplification, but we think of value to sediment the key findings of our study. We have now expanded the discussion to include this point and with reference to MeCP2 co-operativity.

4) As noted above, a significant dependence of MeCP2 binding on salt concentration is documented in the literature. Thus, the differences in the excipients used for the different experimental techniques is of concern. While the authors are applauded for including a Mass Photometry analysis (Extended Figure 2C) it is of concern that it was conducted at 200 mM salt compared with 125 mM salt used in EMSA. It is likely that the mass distributions will be both more complex and larger at the lower salt concentrations of the binding assays based up the sizable Hill coefficients measured by EMSA. If so, the Mass Photometry analysis at 200 mM salt provides an inaccurate picture of the binding species present in the EMSA experiments.
In addition, the mass photometry analysis should include the controls noted above for the EMSA studies. The information provided in the legend is cursory. The expected and measured (with error analysis) molecular weights should be stated. Eyeballing the nucleosome core particle at 215 kDa with MeCP2 about 52 kDa adds up to 267 which is consistent with a single MeCP2 monomer binding to a symmetrically modified site. However, the cartoons in Figure 7 appears to show MeCP2 can bind as a dimer. (The legend to this figure is uninformative.) This apparent discrepancy needs to be clarified.

We agree entirely and were keenly aware of the salt dependence of binding. This is a misunderstanding, all binding experiments reported in the study (EMSA, Mass photometry, SPR, MST, hydroxyl-radical footprinting) were performed at 200mM salt. We have updated the methodology to make this more explicit and expanded the mass photometry and model figure legends as suggested.

5) The conclusion that MeCP2 binds to methylated sites within the nucleosome core is inferred from the binding isotherms but not directly demonstrated. Given that this

observation is the keystone of the whole study, the absence of direct evidence of protection of bound DNA by MeCP2 is a critical deficiency. Since the authors have conducted hydroxyl radical footprinting of the nucleosome, this technique would seem to be the easiest way to obtain this information. Alternatively, DNase 1 footprinting, which was used long ago to map the helical periodicity of DNA wrapper around the histone core, should also be able to provide this information. This analysis should be straightforward for the MBD domain. Mapping the C-terminal contacts is likely to be a more significant technical challenge but would provide valuable support for the manuscripts guiding hypothesis.

We see that mutation in the MBD, that ablates methylated cytosine reading, removes methyl dependency within a nucleosome, strongly suggesting this is a direct interaction (Figure 4B and new Supplementary Figure 9). The binding of MeCP2 to methylated sites is based on the wealth of data we present using 22 different constructs on the nucleosome and built on over 30 years of research that confirms the MeCP2 interaction both *in vitro* and *in vivo*.

MeCP2 footprinting has not been possible in our hands using a hydroxyl-radical approach. We believe this is a technical limitation due to the water mediated nature of MBD-methyl cytosine interaction, and the generation of free radicals disrupting this. Preliminary DNaseI footprinting shows increased protection around site of dyad methylation (reviewer Figure 2 A&B). Furthermore, our preliminary cryo-EM structure of MeCP2 and nucleosome shows direct engagement of the MBD when methylation was placed at a restrictive site (81 meCpA, reviewer Figure 2C).

We are excited to expand on this in future work using multiple methylation sites in connection with exploring how methylated cytosines can be read throughout the nucleosomes and structural rearrangements and nucleosome dynamics that can occur to allow this.

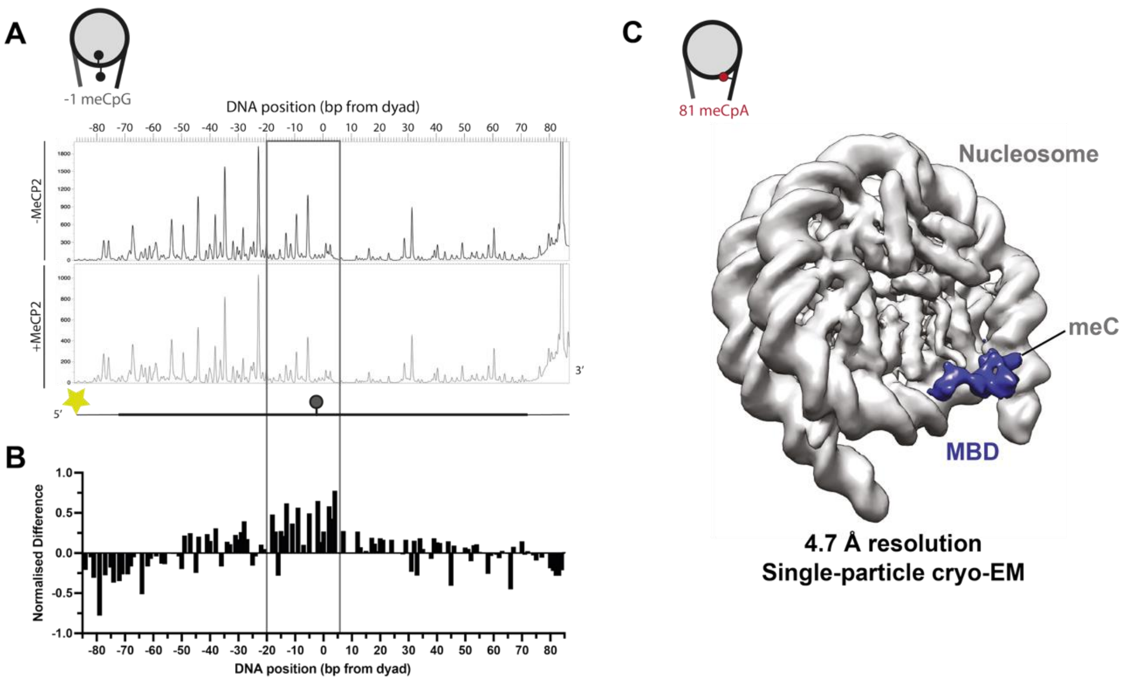

Reviewer Figure 2: Preliminary structural data for MeCP2 binding nucleosomal DNA methylation.

A. DNaseI digest of 5' 6-FAM labelled 15-N_{601}-15 nucleosomes with meCpG at position -1 (grey). Reactions were set up with 50 ng/µl of nucleosome alone (- MeCP2) or pre-incubated with MeCP2 in a 1:2.5 molar ratio for 30 mins at room temperature (+ MeCP2). 2.1 ng/µl DNaseI was added, incubated for 30 seconds and quenched with 227 mM EDTA. Reactions were purified and analysed as described for hydroxyl-radical footprinting. The location of methylated DNA on the analysed strand and footprinting traces is indicated below.

B. Comparison analysis of -/+ MeCP2 DNaseI footprinting traces. Peak heights and size were called using ThermoFisher Connect Microsatellite analysis software. Size in base pair was rounded to the nearest whole integer and converted to position from dyad, whilst height was normalised to the average signal across all called peaks to account for sample loss during ethanol precipitation. Normalised difference was then calculated for each position by $\frac{(-MeCP2)-(+MeCP2)}{(+MeCP2)}$ and plotted in Prism 9 (GraphPad). The plot is aligned to **A** and peaks with the largest positive difference are highlighted by a grey box.

C. Single particle cryo-EM density of an MeCP2 bound 15-N_{601}-15 nucleosome with meCpA at position 81 (grey). Low-resolution density attributed to the MBD of MeCP2 is highlighted in blue. The MBD and linker DNA are shown at a higher threshold due to linker flexibility.

Reviewer #4 (Remarks to the Author):

This is a strong and well-executed manuscript that significantly contributes to our understanding of how MeCP2 recognizes methylated DNA in the context of chromatin. The authors build on earlier reports of a secondary DNA-binding region in the central domain of MeCP2 and convincingly show that this region plays a critical role in enabling methylation sensing within nucleosomes. By demonstrating that this mechanism relies on the presence and length of linker DNA, the study adds an important structural dimension to our understanding of MeCP2 function.

The findings are well aligned with recent work on chromatin-binding proteins, particularly those containing intrinsically disordered regions. Beyond MeCP2 itself, the study has broader implications for how disordered chromatin readers interpret epigenetic marks in compacted DNA, and how features like nucleosome positioning and linker accessibility can modulate that process.

Mechanistically, the data support a model in which multivalent interactions—mediated by both the canonical MBD and a second DNA-binding site—allow MeCP2 to access methylated CpG sites that are otherwise occluded within the nucleosomal core. This is a meaningful addition to our current understanding of MeCP2 function and helps explain why truncated variants lacking this central domain often fail to restore full activity in Rett syndrome models. It also opens the door to refining therapeutic strategies that aim to restore MeCP2 function.

The experimental approach is thorough and well controlled. The combination of in vitro assays (EMSA, MST, SPR) with live-cell imaging (FRAP) provides a solid foundation for the conclusions. Results are consistent across methodologies and clearly support the idea that both methylation and linker DNA are required for proper MeCP2–chromatin engagement.

We thank the reviewer for their time and positive consideration of our work.

That said, there are a few points that I believe would benefit from clarification or further discussion:

1. Direct histone interaction
Previous studies have shown that MeCP2 can interact directly with histones, including their tails. In this manuscript, it's not clear whether any controls were included using histone octamers in the absence of DNA. If such experiments were not performed, I would suggest acknowledging this as a limitation.

No experiments were conducted using histones or octamers in isolation. This is mostly a technical limitation as, in our hands, octamers are insoluble at low physiological salt concentrations in the absence of a chaperones, under the conditions of these assays. We have extended discussion on histone interactions on page 14, and added recognition of this limitation to the manuscript, also on page 14.

2. Binding to core particles
A central observation in the manuscript is that MeCP2 fails to bind nucleosomes lacking linker DNA. While this supports the proposed model where linker interactions are essential for methylation sensing, it raises an unresolved question: if MeCP2 is capable of binding both DNA and histones—as suggested by previous studies—why is there no detectable binding to core nucleosomes in the absence of linker DNA?. I encourage the authors to

address this apparent discrepancy more directly, and to discuss whether it challenges or refines current models of MeCP2–chromatin association.

This raises an interesting point, we showed that MeCP2 fragments can bind to features of histones in the octamer and this can be reduced by histone mutation (previous Extended Data 3A & 5A&B, now Supplementary Figure 6A, 8C&D). However, providing additional linkers negated this effect.

Motivated by this comment, in the absence of competitor DNA we do observe binding of MeCP2 to nucleosome core particles lacking linker DNA (new supplementary figure 8A&B), but no methylation specificity. Furthermore, no distinct complex is resolved on a gel, suggesting instead that aggregation predominates likely through nonspecific electrostatic interactions.

We suggest that MeCP2 recognises features of DNA and histones in isolation that are possibly masked when wrapped into a nucleosome. Indeed, for the DNA interaction we think this is an important feature; MeCP2 preferentially engages linear linker-like DNA. In the absence of linear DNA, electrostatic interactions to bent histone-bound DNA are possible but not as preferred.

It is likely that we are missing elements from our system by using mono-nucleosomes. For example, it has been previously shown that the C-terminal region of MeCP2 is implicated in interactions with nucleosome arrays, but dispensable otherwise (Ghosh *et al.,* 2010 *Biochemistry*). We are excited to carry on this work in longer arrays and further clarify the role of histone and DNA interaction of MeCP2.

3. Interpretation of binding affinities
EMSA is widely used to quantify protein–DNA interactions, but its ability to distinguish true affinity from cooperative or multivalent effects is limited. Since MeCP2 has been described (here and elsewhere) to bind DNA through multiple motifs, it would be helpful to state whether the Kd values were calculated assuming a simple one-site model, or if multivalency was taken into account in any way, and discuss what would the consequences of overlooking this aspect be.

This is a good point, the interaction of MeCP2 with nucleosomes in both the EMSA and orthologous MST and SPR assays is clearly complex, involving multiple interaction interfaces and an added role of unlabelled competitor DNA. We purposely reported apparent Kds rather than absolute Kds and instead focus on internal comparisons within controls run at the same time.

The data was fitted using the Hill equation assuming specific binding. The Hill coefficients did show a large degree of variation suggesting extensive multivalency. We have made more explicit how the data was fitted in the methods and main text and discussed the complex binding mode on page 14.

4. Reference for R133G
The manuscript mentions that the R133G mutation disrupts methylation recognition via the MBD. Please include a supporting reference for this or provide additional data in the supplement.

We have now included binding data for the R133G mutation, which does remove methyl binding specificity of the MBD (Supplementary Figure 9D).

This was originally a cloning error; our intention was to make the R133C Rett-syndrome mutation. However we note that multiple mutations of Arg-133 have been described in ClinVar, including R133G.
R133C has been previously well characterised to disrupt methyl cytosine recognition. By removing this key arginine we predict that that the R133G mutation would behave similarly.

In summary, this is a well-written and impactful study that makes a clear and meaningful contribution to the field. Addressing the points above would strengthen the mechanistic model and improve interpretability, particularly around the limitations of the experimental design. I support publication pending minor revisions.

Reviewer #5 (Remarks to the Author):

I co-reviewed this manuscript with one of the reviewers who provided the listed reports. This is part of the Nature Communications initiative to facilitate training in peer review and to provide appropriate recognition for Early Career Researchers who co-review manuscripts.

We are grateful to the reviewer for their time and consideration. Please see points above

FIGURE CHANGES AND NEW DATA SUMMARY

Main figures

- Figure 2
  - Panel A-B New data – EMSAs of MeCP2 binding to 37-N601-27 containing 4 new sites of meCG. Gel images moved to Supplementary Figure 4.
  - Panel C-D – moved from Ex.Data 2A-B. Gel images moved to Supplementary Figure 5.

- Table 1
  - Values from Supplementary Table 3, relevant to Figure1-2, now summarised in this table.
  -

- Figure 3 (previously Figure 2)
  - Panel A and B – gel images moved to Supplementary Figure 7.
  - Panel C – moved from Ex.Data 4A.

- Figure 4 (previously Figure 3)

- Figure 5 (previously Figure 4)
  - Panel B, D - gel images moved to Supplementary Figure 10.
  - Panel E – gel images moved to Supplementary Figure 9.

- Figure 6 (previously Figure 5)
  - Panel A – gel images moved to Supplementary Figure 12.

- Figure 7 (previously Figure 6)
  - Panel B-C – gel images moved to Supplementary Figure 15.

- Figure 8 (previously Figure 7)

Supplementary Figures

Previous 'Ex.Data' and 'Supp fig' now merged

- Supplementary Figure 1
  - Panel A – moved from supp fig 1C
  - Panel B – moved from supp fig 1B
  - Panel C – moved from supp fig 1D
  - Panel D New data – Hydroxyl radical footprinting of modified Widom601 sequences to check wrapping.

- Supplementary Figure 2
  - Panel A – moved from supp fig 1A
  - Panel B - moved from supp fig 1E
  - Panel C New data – MeCP2 MBD modelled into nucleosome structures at sites of meCG.
  - Panel D New data – solvent accessibility plots for both DNA strands around a nucleosome. Sites of meCG again highlighted.
  - Panel E – Panel B New data – EMSA of MeCP2 binding to 15-N601-15 DNA. Local DNA sequence around meCG sites shown.

- Supplementary Figure 3

- Panel A - Mass photometry panel from Ex.Data 2C. Mass values and statistics added, cartoons and description modified for clarity.

- Supplementary Figure 4
    - Panel A New data – MeCP2 MBD modelled into 4 new sites of meCG on the nucleosome.
    - Panel B-C New data – EMSA gel images of MeCP2 binding to 37-N601-27 containing 4 new sites of meCG. Local DNA sequence around meCG sites shown.

- Supplementary Figure 5
    - Panel A New data – EMSA of MeCP2 binding to 16-N603-30 DNA.
    - Panel B-C – moved from Ex.Data 2A-B.

- Supplementary Figure 6 (previously Ex.Data 3)

- Supplementary Figure 7
    - Panel A – gel images moved from Figure 2A
    - Panel B-D – moved from Ex.Data 4B-D.

- Supplementary Figure 8
    - Panel A&B New data – EMSAs of MeCP2 and MBD binding to N601 nucleosome core particles, without competitor DNA, with various histone variants used throughout the paper.
    - Panel C-E – moved from Ex.Data 5A-C

- Supplementary Figure 9
    - Panel A-B – moved from Ex.Data 5D-E
    - Panel C – gel images moved from Figure 4E
    - Panel D New data – EMSAs of MeCP2 R133G binding to 15-N601-15 nucleosomes and DNA.

- Supplementary Figure 10
    - Panel A – moved from supp fig 2C
    - Panel B – gel images moved from Figure 4B
    - Panel C – moved from Ex.data 6A
    - Panel D – moved from supp fig 2D
    - Panel E – gel images moved from Figure 4D
    - Panel F – moved from Ex.data 6B

- Supplementary Figure 11
    - Panel A-C – moved from Ex.data 6C-E

- Supplementary Figure 12
    - Panel A – moved from supp fig 2E
    - Panel B – moved from Figure 5A
    - Panel C – moved from supp fig 3

- Supplementary Figure 13 (previously Ex.data 7)

- Supplementary Figure 14 (previously Ex.data 8)

- Supplementary Figure 15
    - Panel A-C – moved from Ex.Data 9A-C

    - Panel D – gel images moved from Figure 6B
    - Panel E – moved from Ex.Data 9D
    - Panel F – gel images moved from Figure 6C
    - Panel G – moved from Ex.Data 9E

- Supplementary Figure 16 (previously Ex.data 10)

Source Data Files

- Source data file 1 – values used to generate each panel in Main Figures.
- Source data file 2 – raw images of each gel (including repeats) used in Main Figures.
- Source data file 3 – values used to generate each panel in Supplementary Figures.
- Source data file 4 – raw images of each gel (including repeats) used in Supplementary Figures.
- Source data file 5 – Examples of EMSA band quantifications

MeCP2 requires interactions with nucleosome linker DNA to read chromatin DNA methylation

REVIEWERS' COMMENTS

Reviewer #2 (Remarks to the Author):

We have no further comments to convey to the authors.

We thank the reviewer for their insight throughout this process.

Reviewer #4 (Remarks to the Author):

I appreciate the authors' thoughtful and detailed responses to the concerns raised in my initial review. The additional data and clarifications they have provided strengthen the manuscript and help address several key points of interpretation and methodology.

Thank you for addressing the point regarding MeCP2–histone interactions. I understand the technical limitations in assaying octamer binding without DNA, and I find the expanded discussion in the revised manuscript appropriate. The idea that MeCP2 may recognize DNA and histone features individually, but that these are masked or altered in the nucleosomal context, is plausible. The proposed contribution of linker geometry to multivalent engagement adds useful mechanistic insight. I consider the issue adequately addressed given the current experimental scope.

On the topic of binding affinity measurements, I find the use of apparent Kd values and internal comparisons reasonable, and I appreciate that the authors have now described the use of the Hill equation more explicitly. The variability in Hill coefficients does suggest a complex and multivalent binding mode, which is consistent with the molecular features of MeCP2. These additions help improve the transparency and reproducibility of the data interpretation.

The inclusion of new binding data for the R133G mutation is helpful. Regarding the key role of the residue in methylaiton recognition, The mutation was likely to have the said effect, but having experimental data backing this up is highly necesary. Keynote: There is a typo in page 14 line 394: It should be "MeCP2 can RECOGNISE...." instead of "recognises".

In summary, I believe the authors have addressed the main concerns raised during the first round of review with rigor and transpa I support acceptance of this revised version.rency. The additional experiments and revisions improve the manuscript both conceptually and technically.

We thank the reviewer for the positive appraisal and insightful comments. Thank you for highlighting the typo, we have modified this in the manuscript.

Reviewer #5 (Remarks to the Author):

I co-reviewed this manuscript with one of the reviewers who provided the listed reports. This is part of the Nature Communications initiative to facilitate training in peer review and to provide appropriate recognition for Early Career Researchers who co-review manuscripts.
